# Supplementary figures and images for: Depletion or cleavage of cohesin during anaphase differentially affects chromatin structure and segregation
Source: eLife. 2022 Oct 5;11:e80147. doi: 10.7554/eLife.80147 (PMC9586560; doi:10.7554/eLife.80147)

$\alpha$ MYC

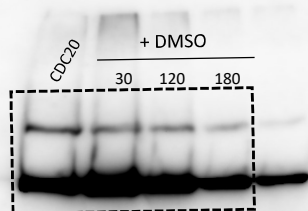

$\alpha$ PGK1

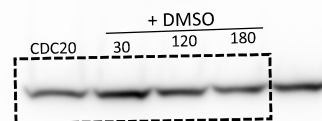

$\alpha$ MYC

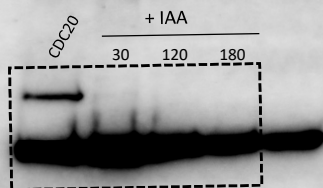

$\alpha$ PGK1

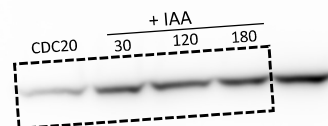

Supplement: Figure 1—source data 1. [file elife-80147-fig1-data1.zip › Figure 1- Source data 1/Figure_1-_Source_data_1.pdf]

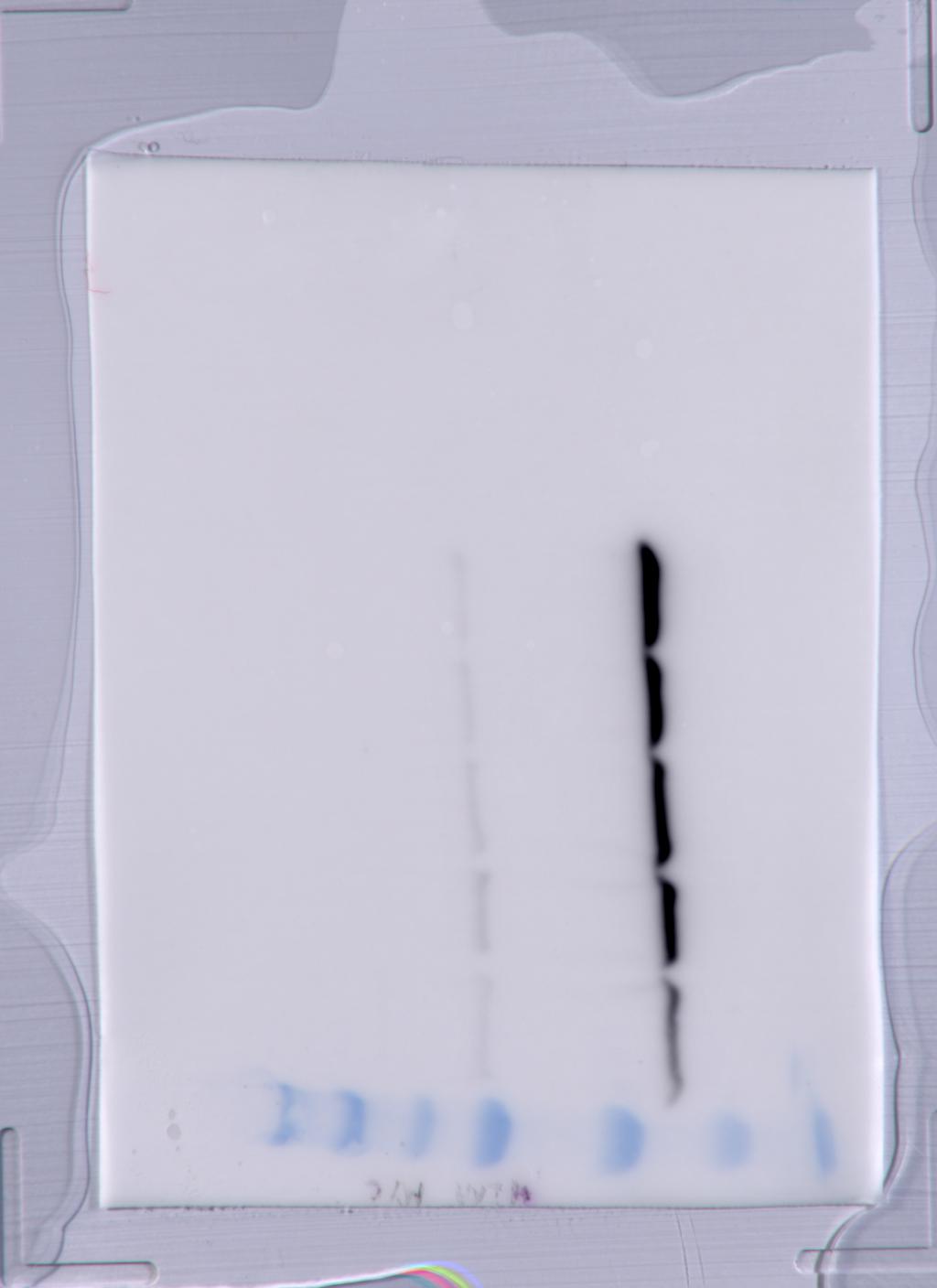

Supplement: Figure 1—source data 1. [file elife-80147-fig1-data1.zip › Figure 1- Source data 1/aPgk1_IAA/teto 469 iaa 2018.09.01_20.20.26_Ch+Marker.jpg]

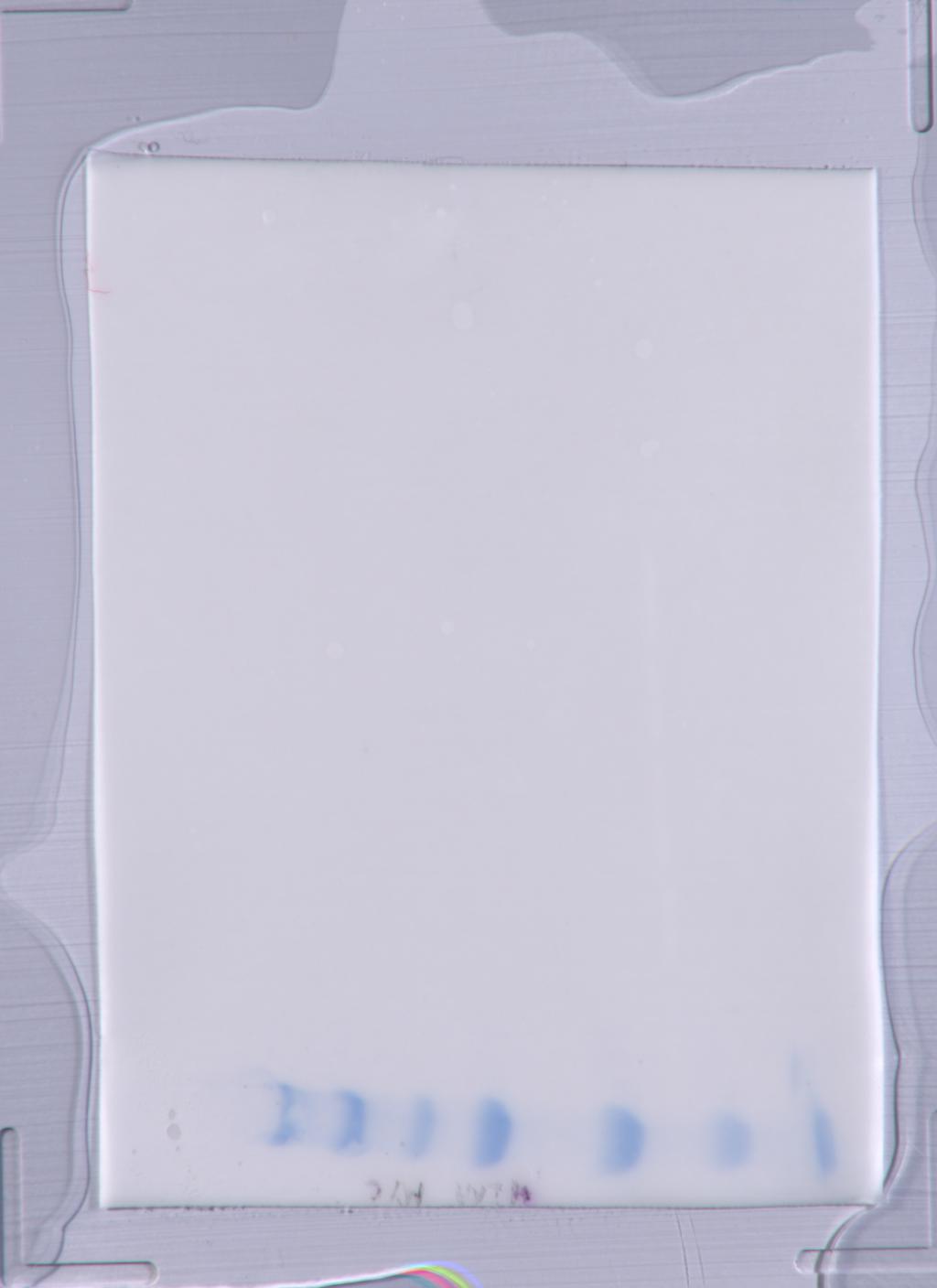

Supplement: Figure 1—source data 1. [file elife-80147-fig1-data1.zip › Figure 1- Source data 1/aPgk1_IAA/teto 469 iaa 2018.09.01_20.20.26_Ch-Marker.jpg]

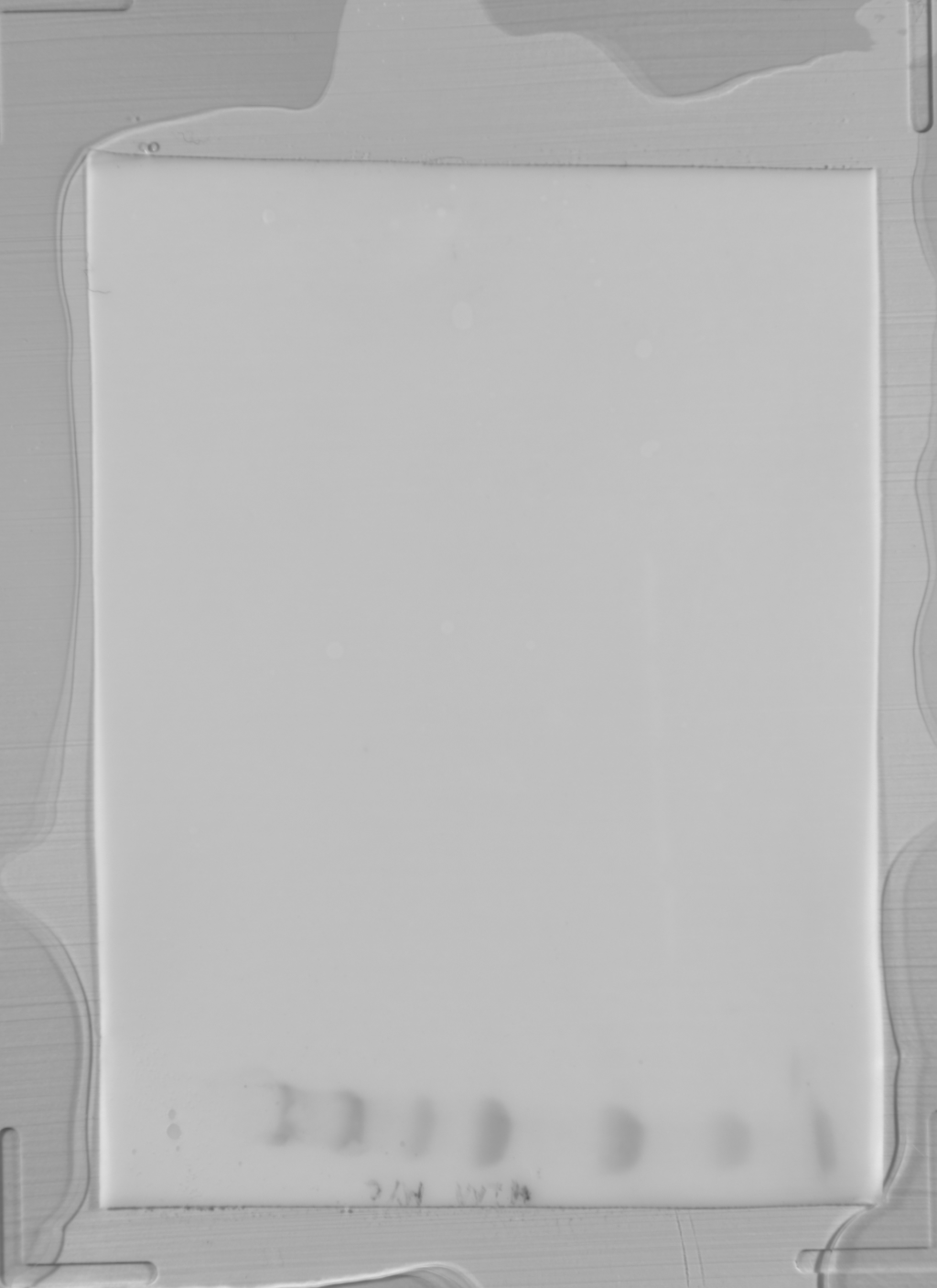

Supplement: Figure 1—source data 1. [file elife-80147-fig1-data1.zip › Figure 1- Source data 1/aPgk1_IAA/teto 469 iaa 2018.09.01_20.20.26_Ch-Marker.tif]

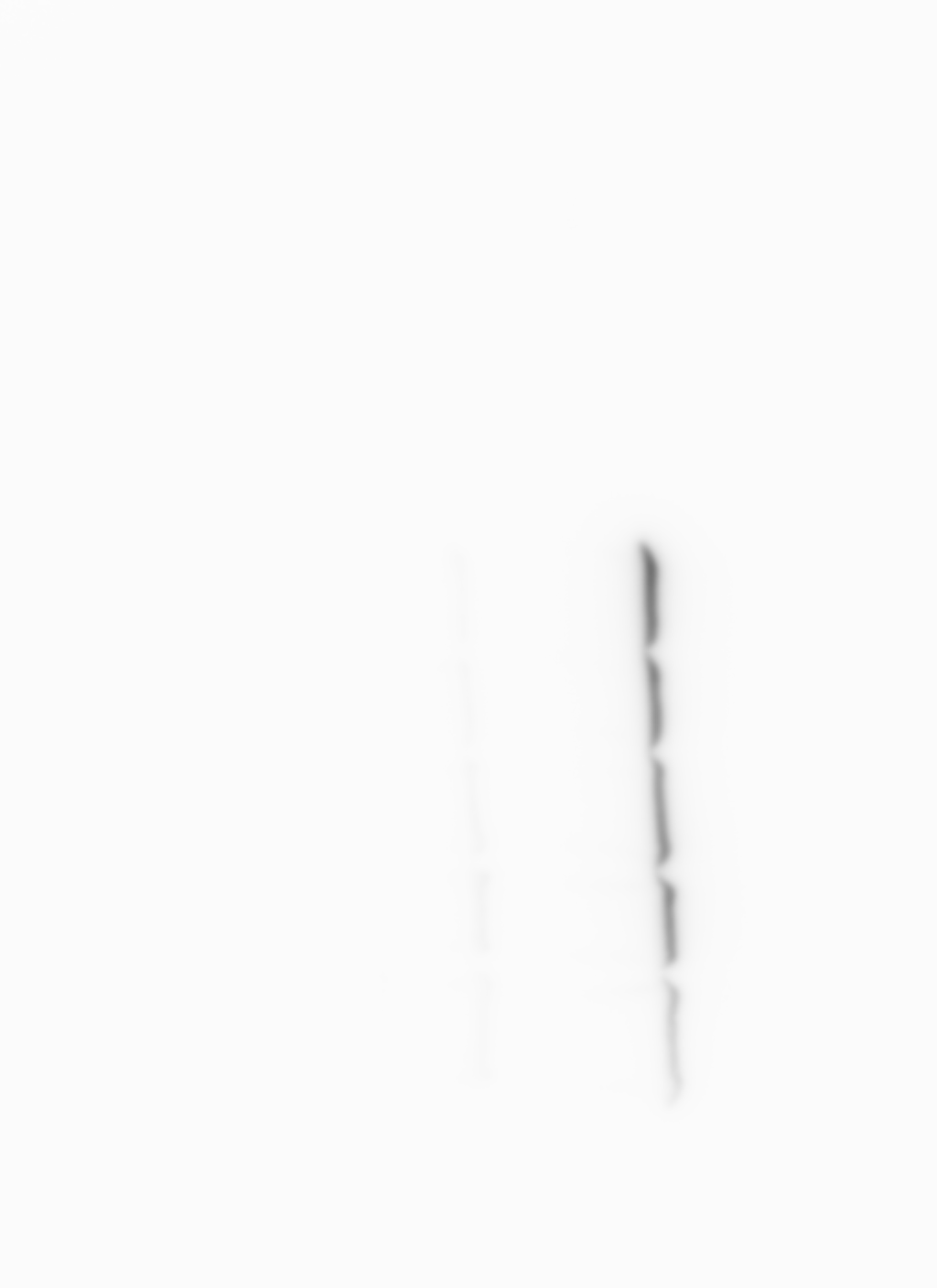

Supplement: Figure 1—source data 1. [file elife-80147-fig1-data1.zip › Figure 1- Source data 1/aPgk1_IAA/teto 469 iaa 2018.09.01_20.20.26_Ch.tif]

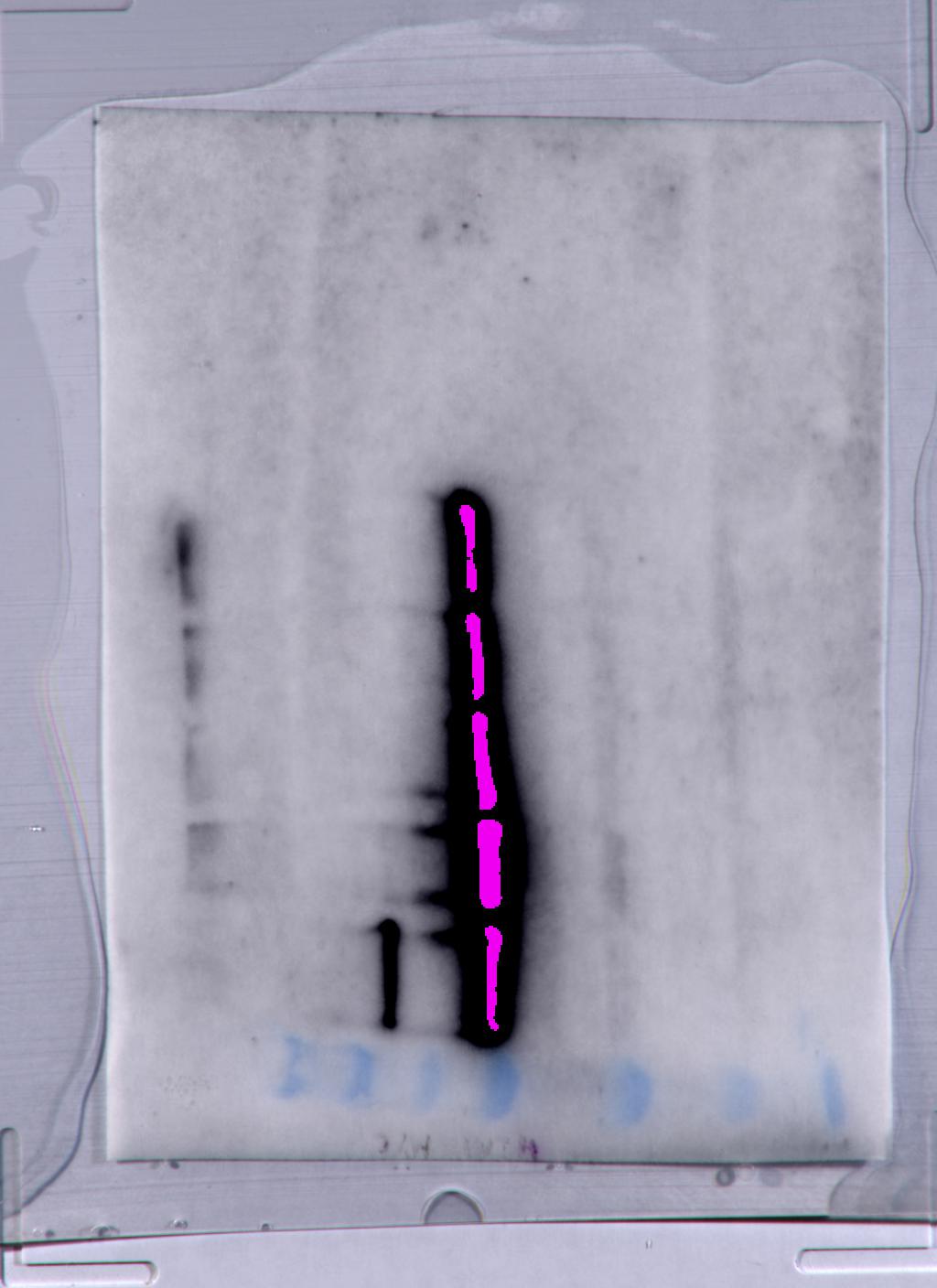

Supplement: Figure 1—source data 1. [file elife-80147-fig1-data1.zip › Figure 1- Source data 1/aMYC_Mcd1&OsTir_IAA/scc1aid iaa 2018.08.31_14.29.41-23_Ch+Marker.jpg]

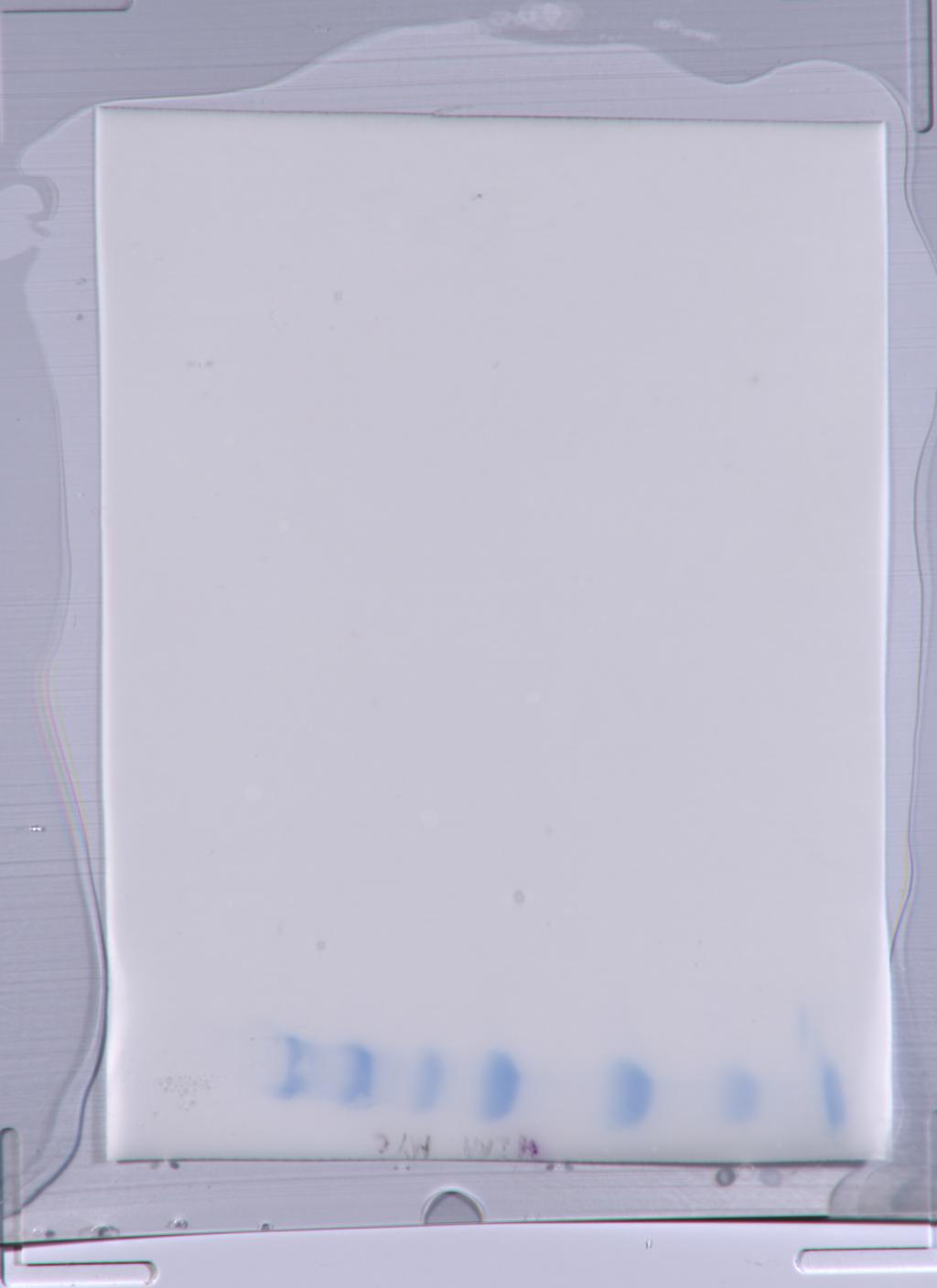

Supplement: Figure 1—source data 1. [file elife-80147-fig1-data1.zip › Figure 1- Source data 1/aMYC_Mcd1&OsTir_IAA/scc1aid iaa 2018.08.31_14.29.41-23_Ch-Marker.jpg]

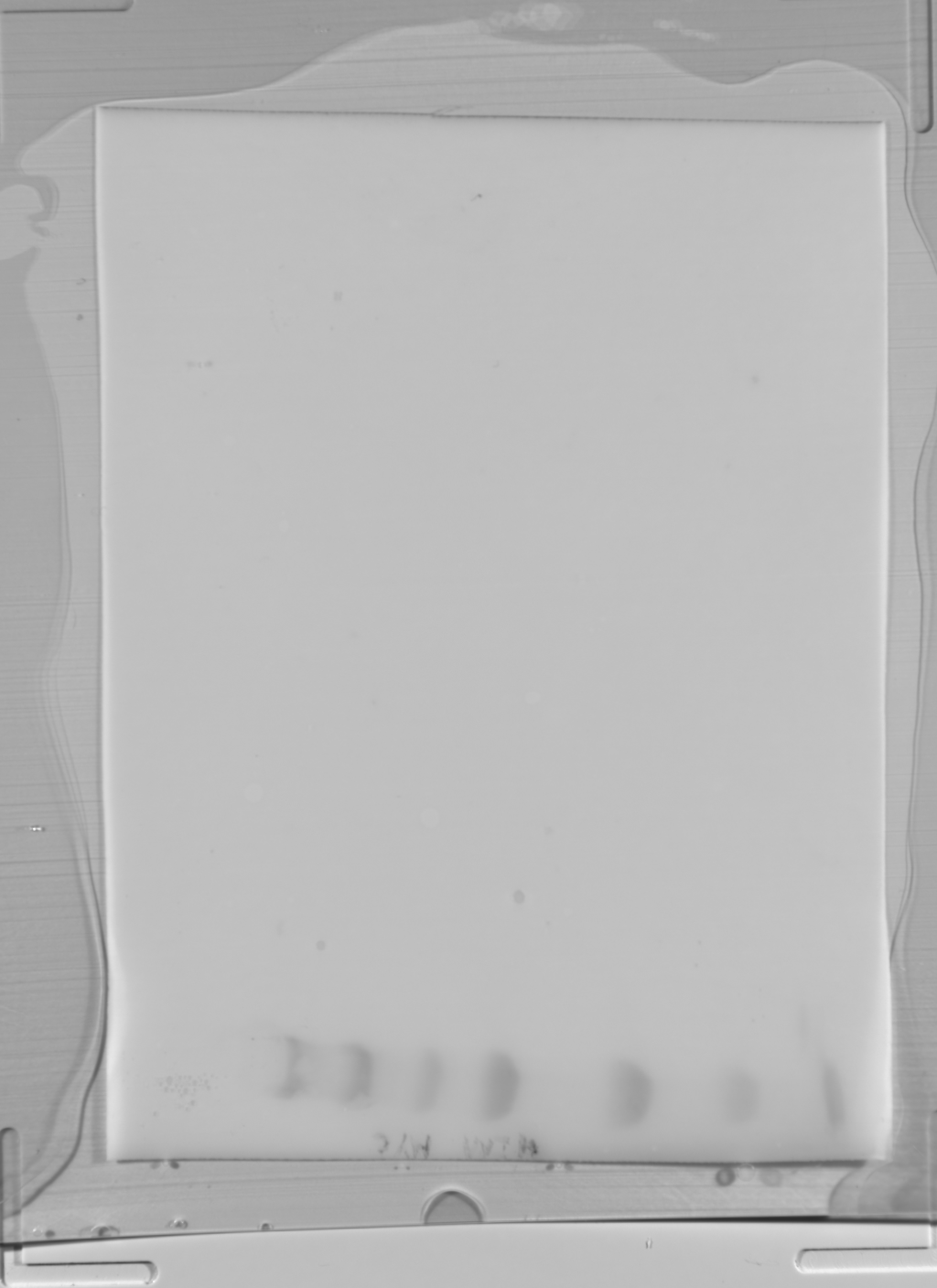

Supplement: Figure 1—source data 1. [file elife-80147-fig1-data1.zip › Figure 1- Source data 1/aMYC_Mcd1&OsTir_IAA/scc1aid iaa 2018.08.31_14.29.41-23_Ch-Marker.tif]

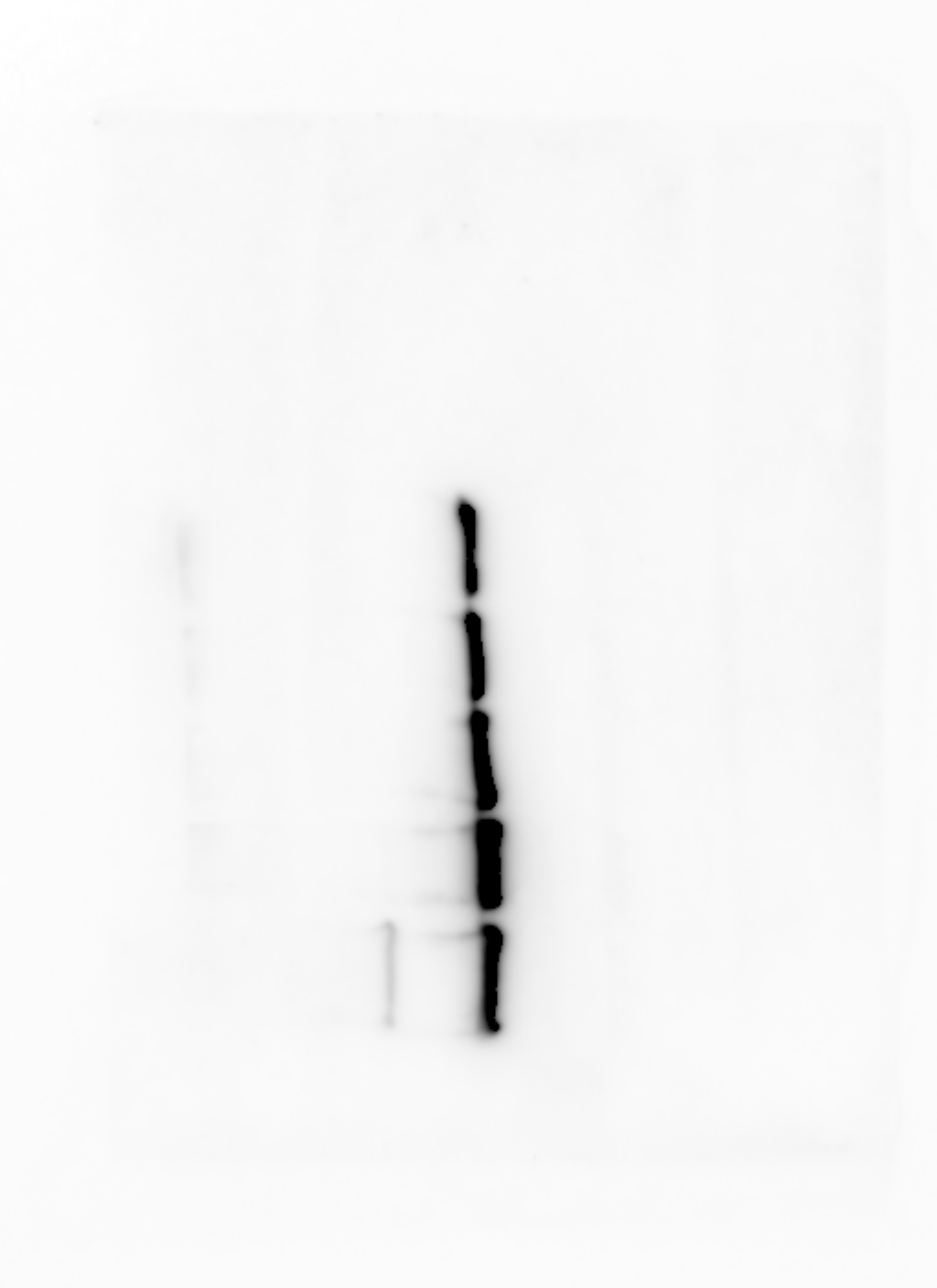

Supplement: Figure 1—source data 1. [file elife-80147-fig1-data1.zip › Figure 1- Source data 1/aMYC_Mcd1&OsTir_IAA/scc1aid iaa 2018.08.31_14.29.41-23_Ch.tif]

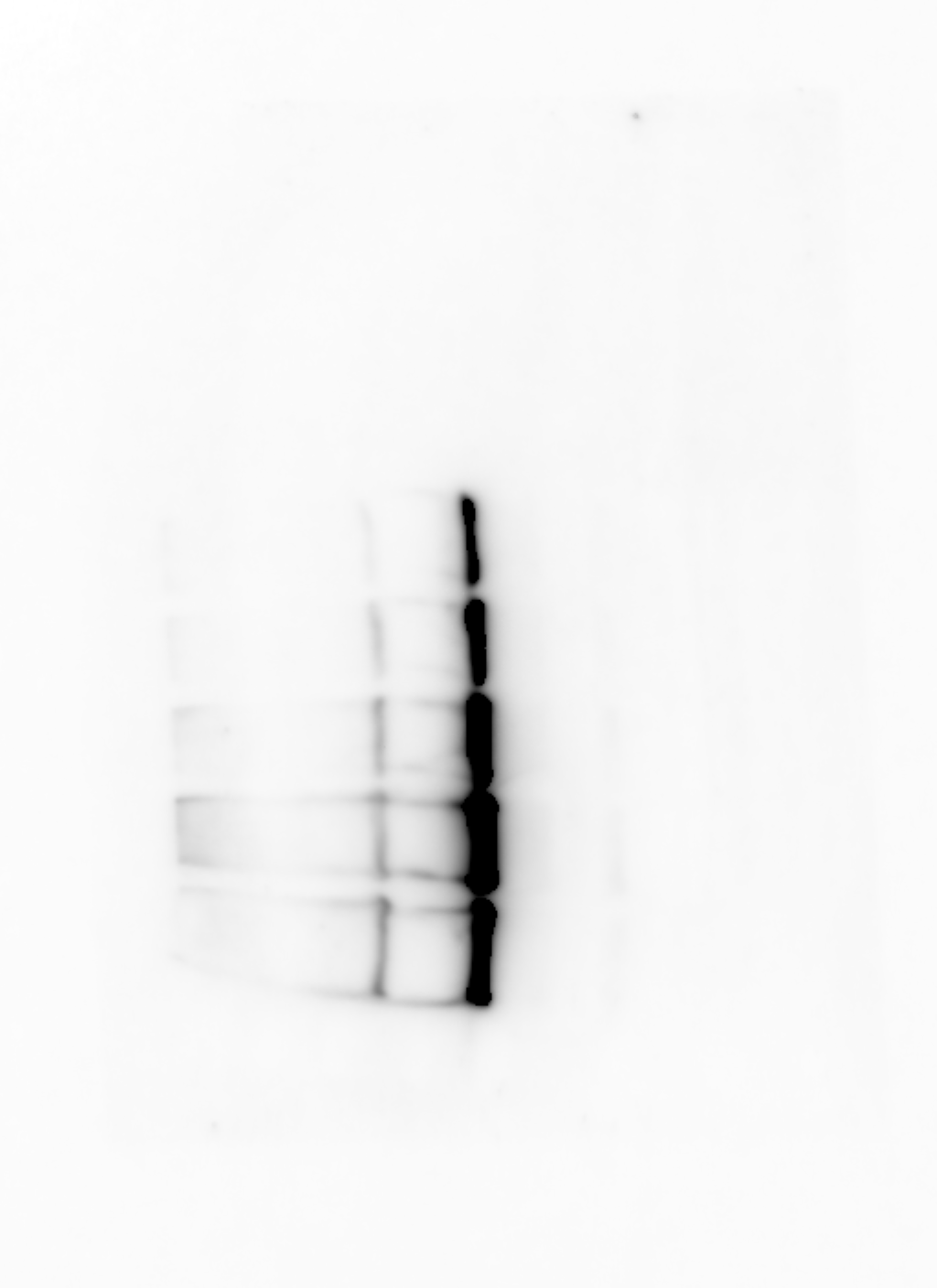

Supplement: Figure 1—source data 1. [file elife-80147-fig1-data1.zip › Figure 1- Source data 1/aMYC_Mcd1&OsTir_DMSO/scc1aid etoh 2018.08.31_14.36.52-23_Ch.tif]

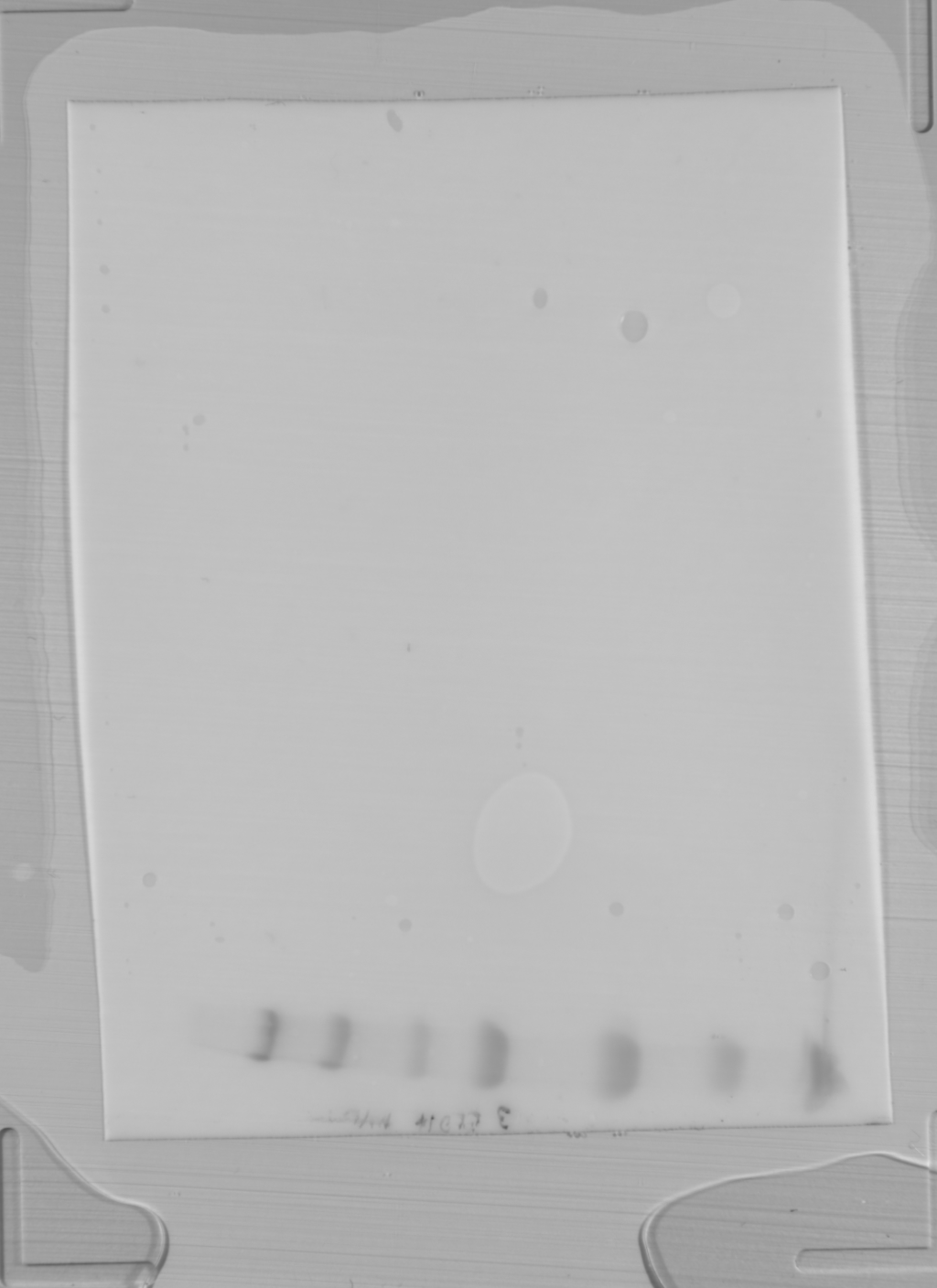

Supplement: Figure 1—source data 1. [file elife-80147-fig1-data1.zip › Figure 1- Source data 1/aMYC_Mcd1&OsTir_DMSO/scc1aid etoh 2018.08.31_14.36.52-23_Ch-Marker.tif]

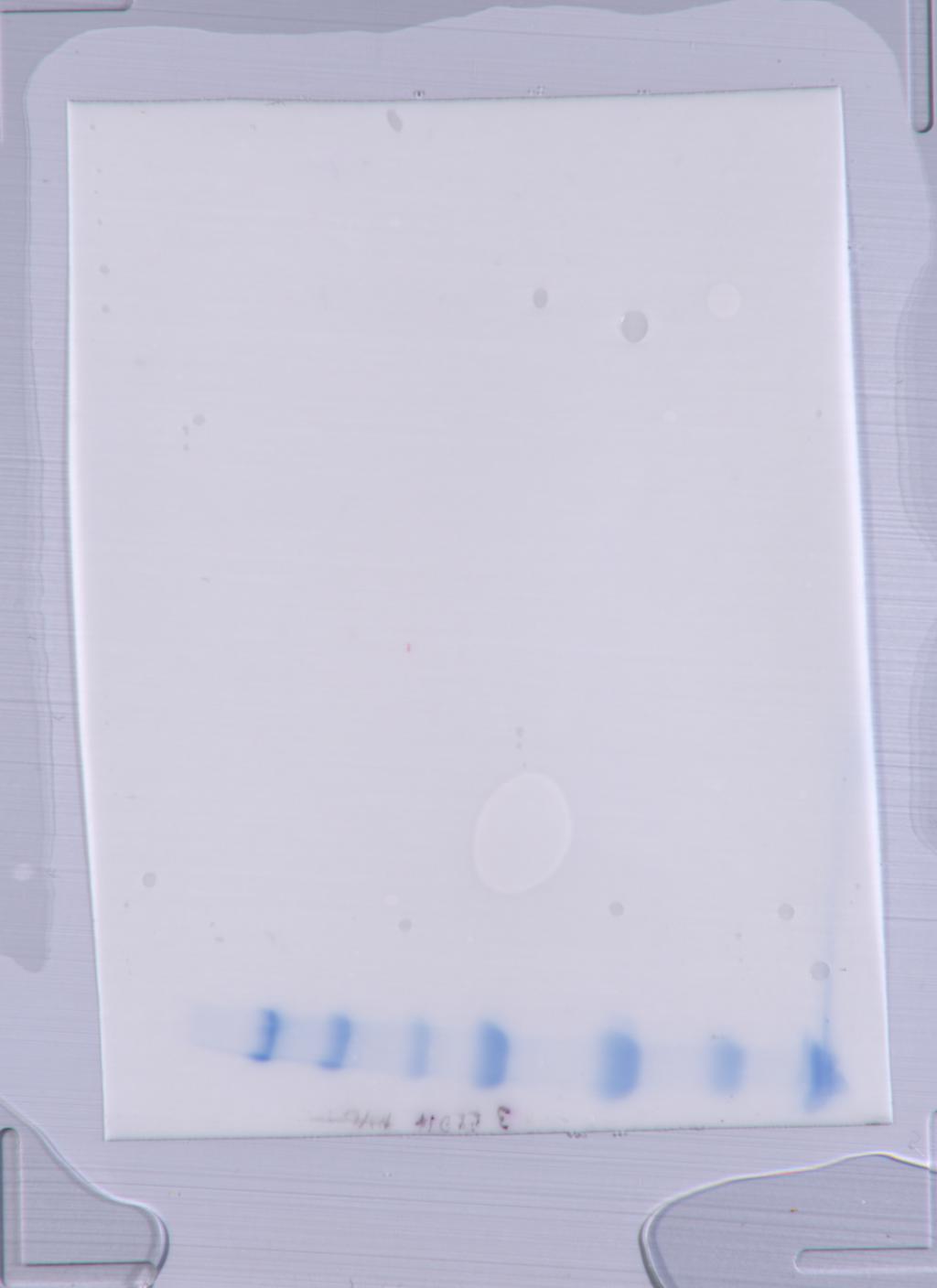

Supplement: Figure 1—source data 1. [file elife-80147-fig1-data1.zip › Figure 1- Source data 1/aMYC_Mcd1&OsTir_DMSO/scc1aid etoh 2018.08.31_14.36.52-23_Ch-Marker.jpg]

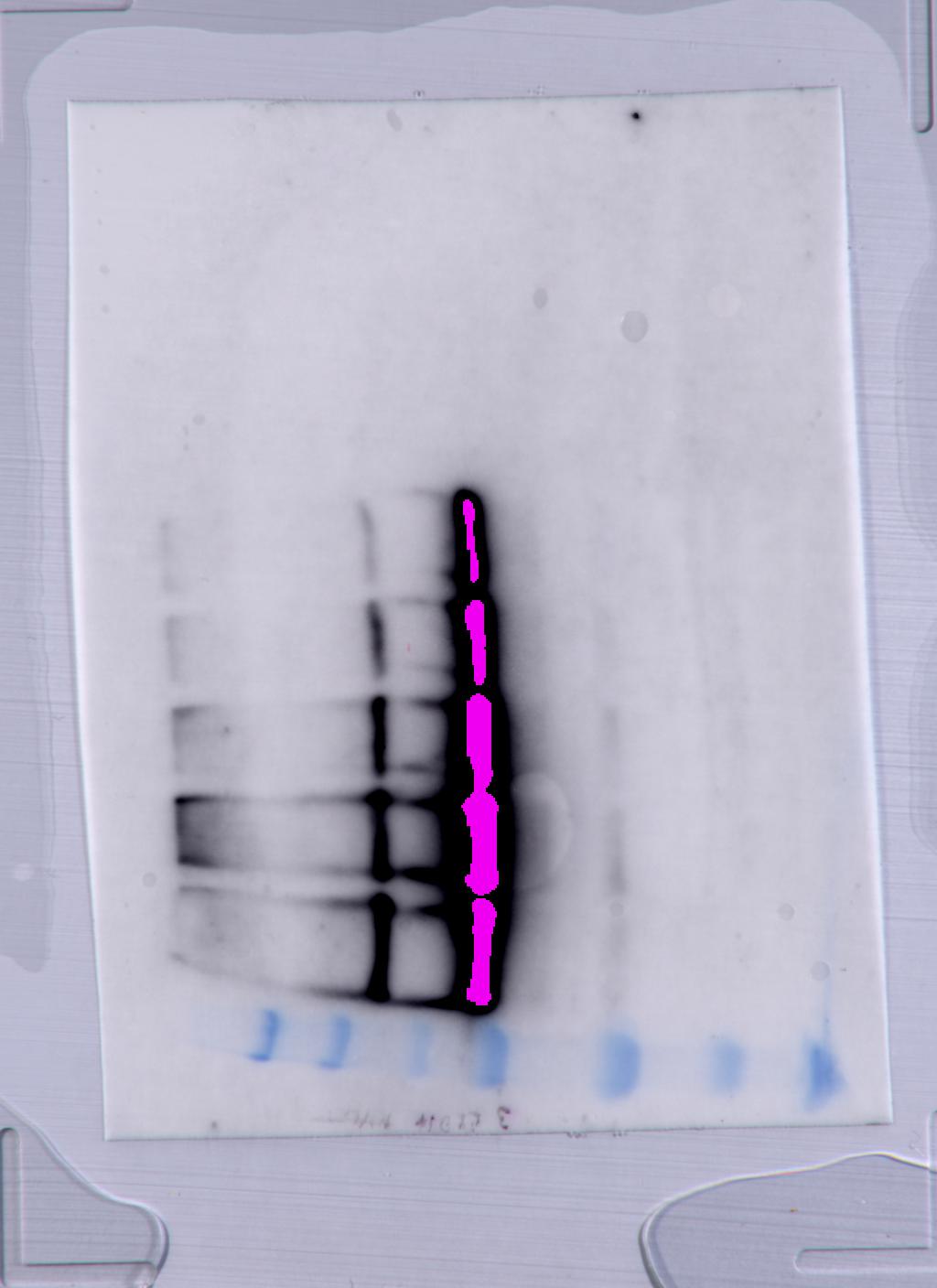

Supplement: Figure 1—source data 1. [file elife-80147-fig1-data1.zip › Figure 1- Source data 1/aMYC_Mcd1&OsTir_DMSO/scc1aid etoh 2018.08.31_14.36.52-23_Ch+Marker.jpg]

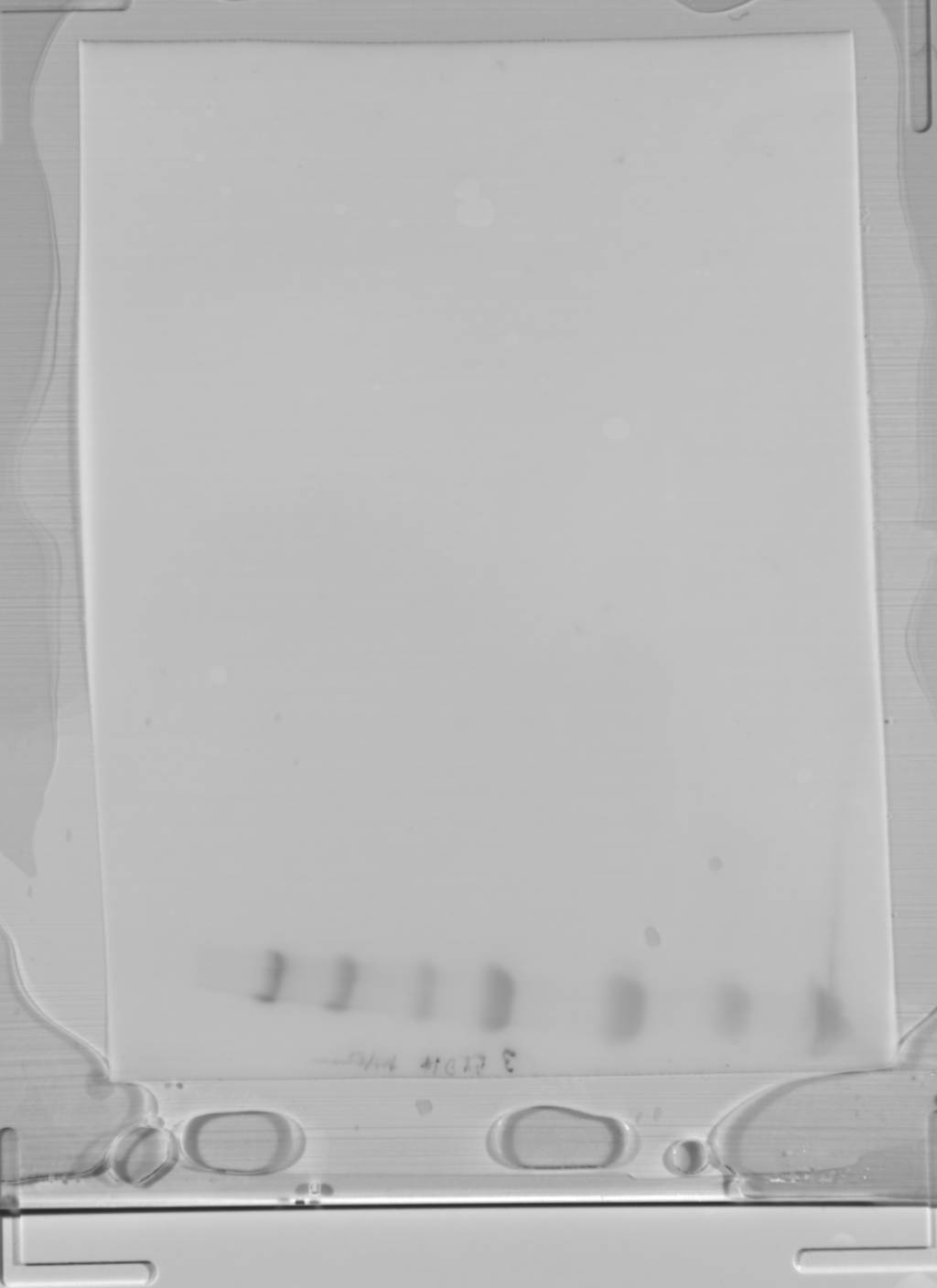

Supplement: Figure 1—source data 1. [file elife-80147-fig1-data1.zip › Figure 1- Source data 1/aPgk1_DMSO/teto 469 etoh 2018.09.01_20.22.58_Ch-Marker.tif]

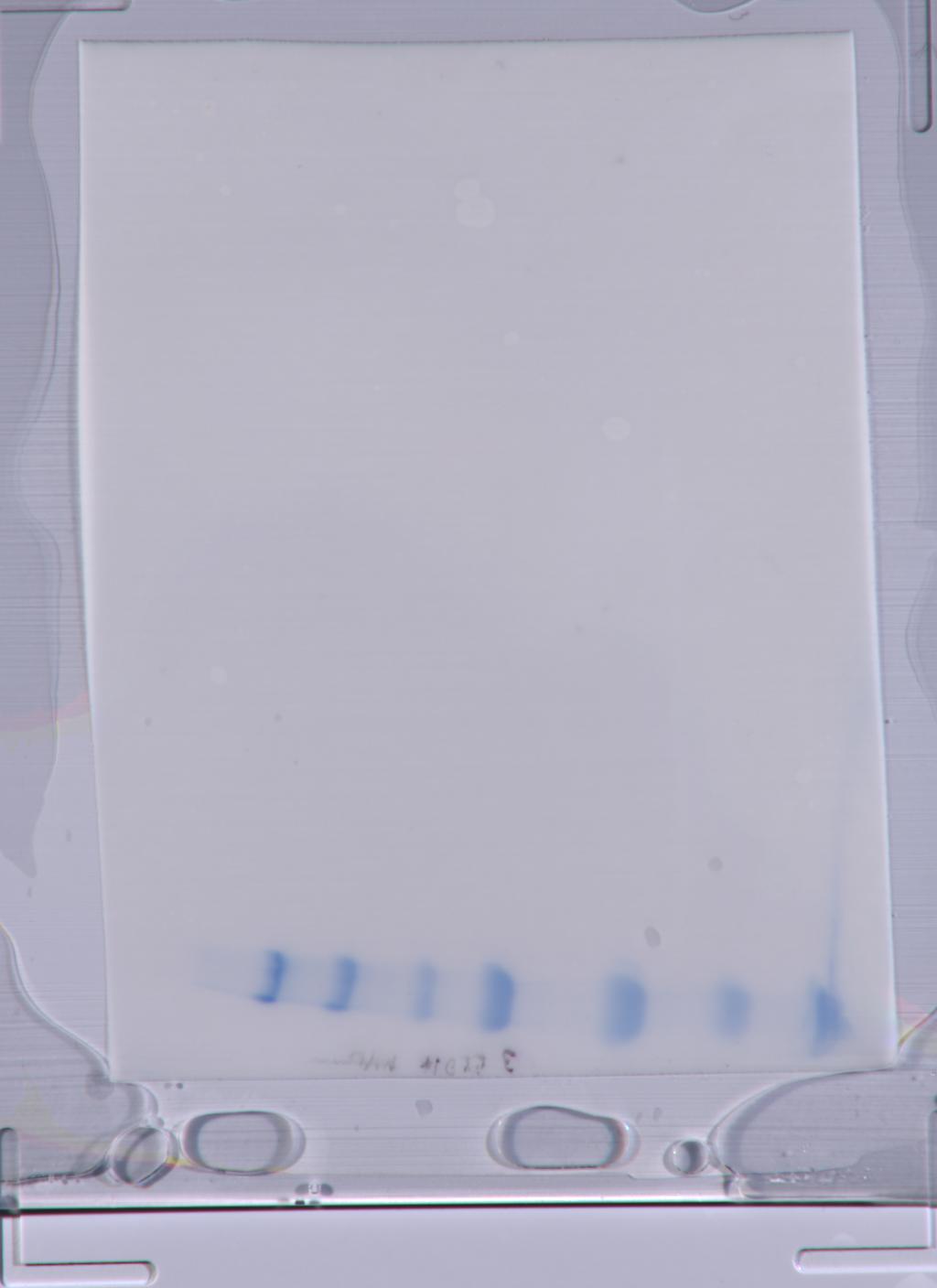

Supplement: Figure 1—source data 1. [file elife-80147-fig1-data1.zip › Figure 1- Source data 1/aPgk1_DMSO/teto 469 etoh 2018.09.01_20.22.58_Ch-Marker.jpg]

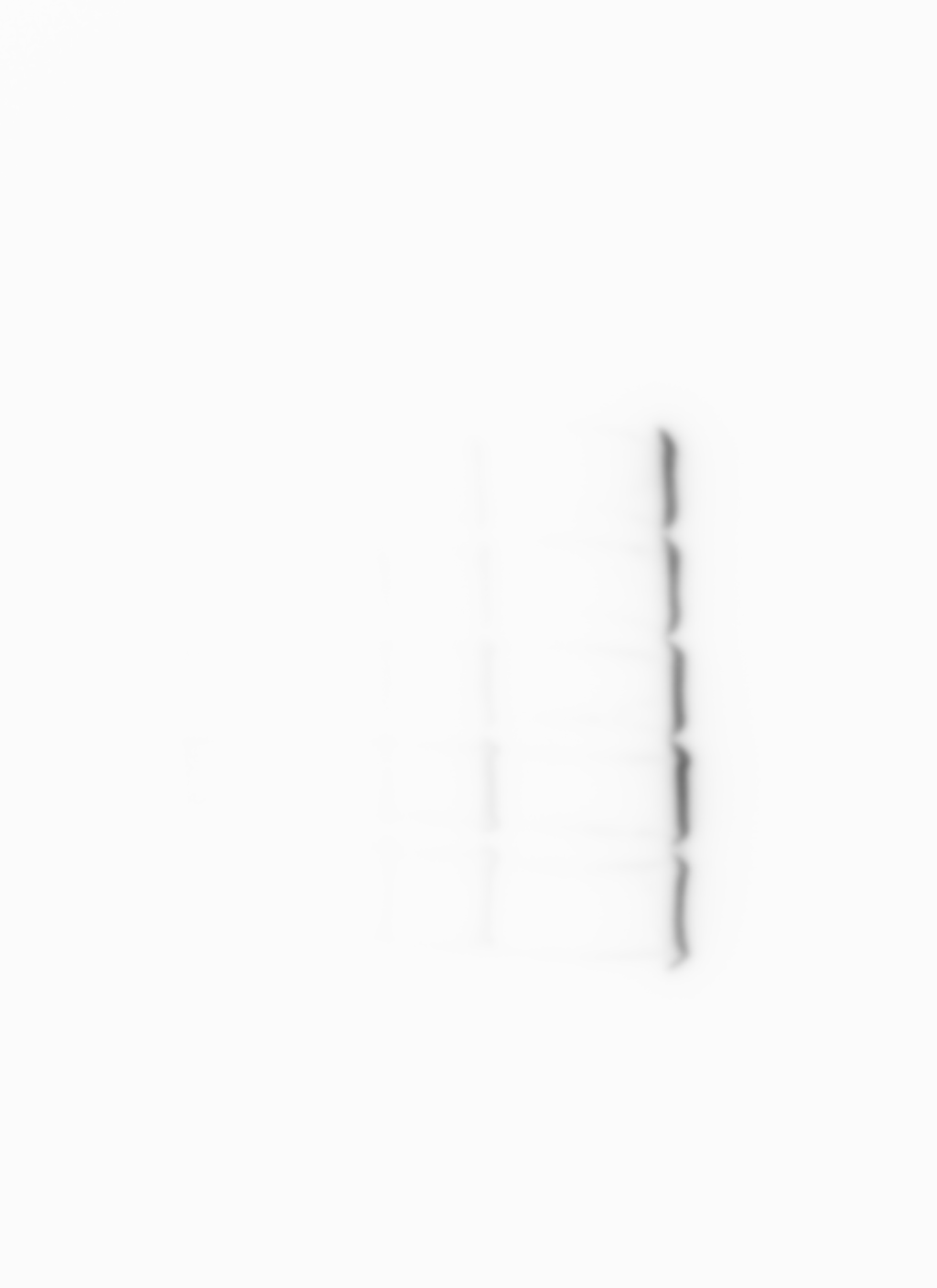

Supplement: Figure 1—source data 1. [file elife-80147-fig1-data1.zip › Figure 1- Source data 1/aPgk1_DMSO/teto 469 etoh 2018.09.01_20.22.58_Ch.tif]

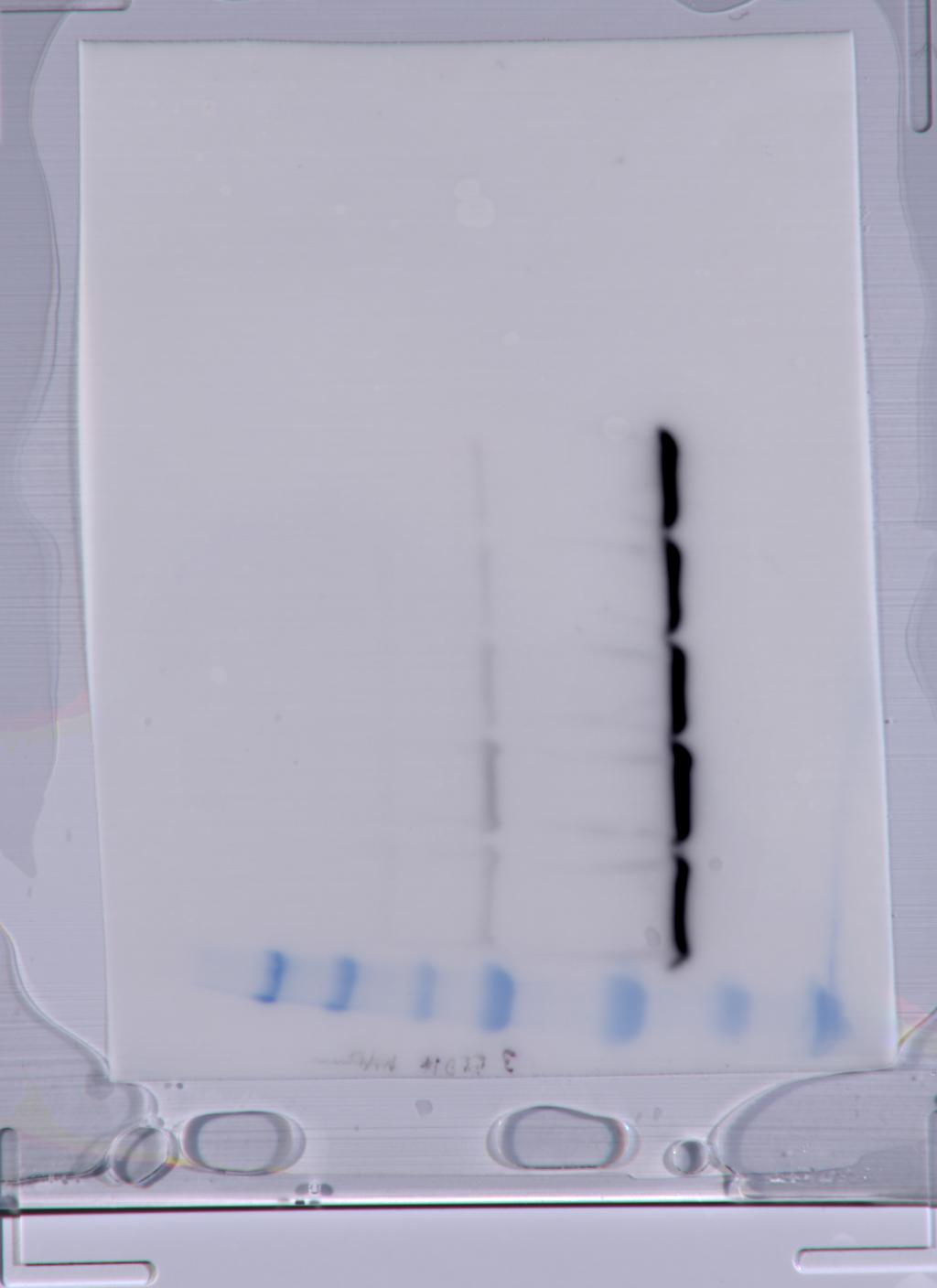

Supplement: Figure 1—source data 1. [file elife-80147-fig1-data1.zip › Figure 1- Source data 1/aPgk1_DMSO/teto 469 etoh 2018.09.01_20.22.58_Ch+Marker.jpg]

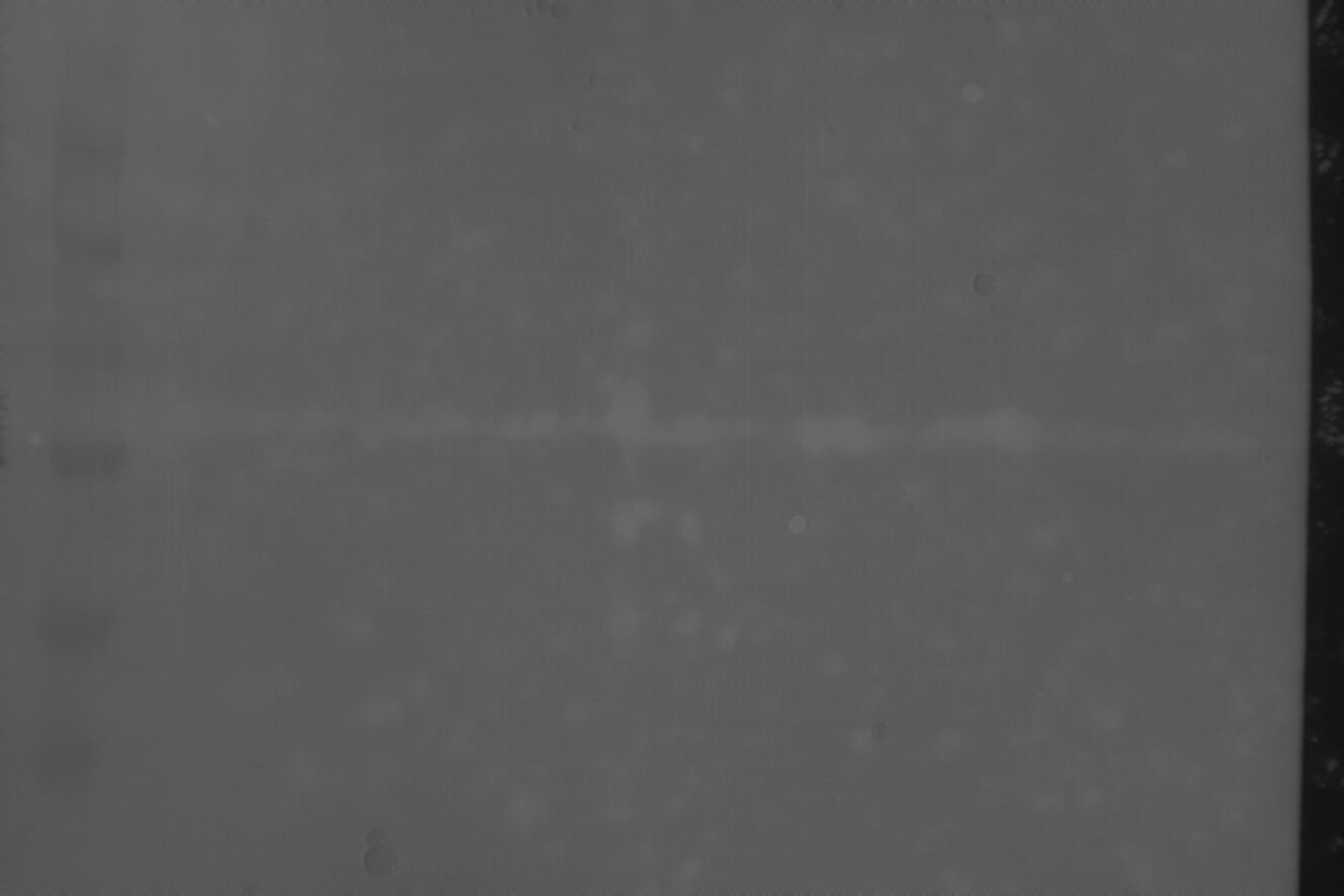

Supplement: Figure 1—figure supplement 1—source data 1. [file elife-80147-fig1-figsupp1-data1.zip › Figure 1- Figure Supplement 1- Source data 1/20170721_2312.gel]

$\alpha$ MYC

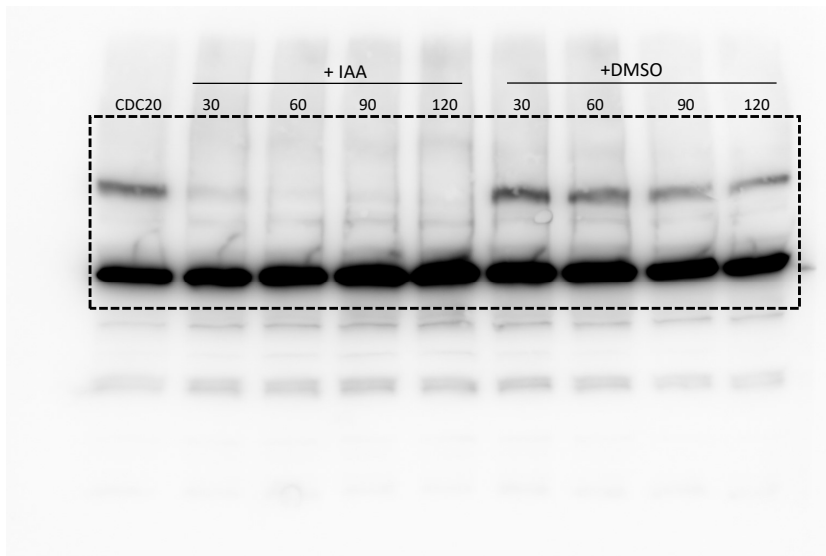

Supplement: Figure 1—figure supplement 1—source data 1. [file elife-80147-fig1-figsupp1-data1.zip › Figure 1- Figure Supplement 1- Source data 1/Figure 1_Figure_supplement_1-_source_data_1.pdf]

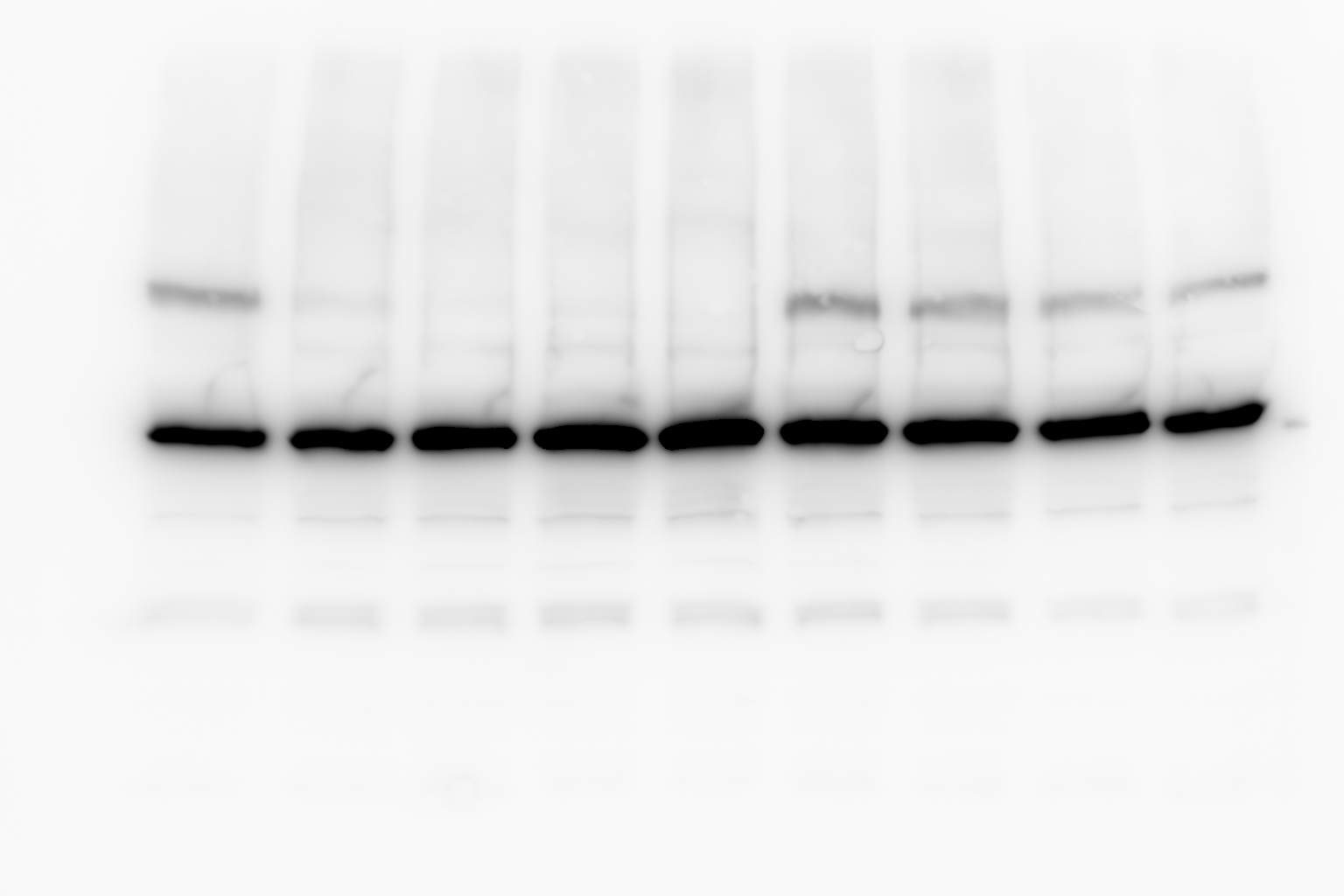

Supplement: Figure 1—figure supplement 1—source data 1. [file elife-80147-fig1-figsupp1-data1.zip › Figure 1- Figure Supplement 1- Source data 1/20170721_2311_15.gel]

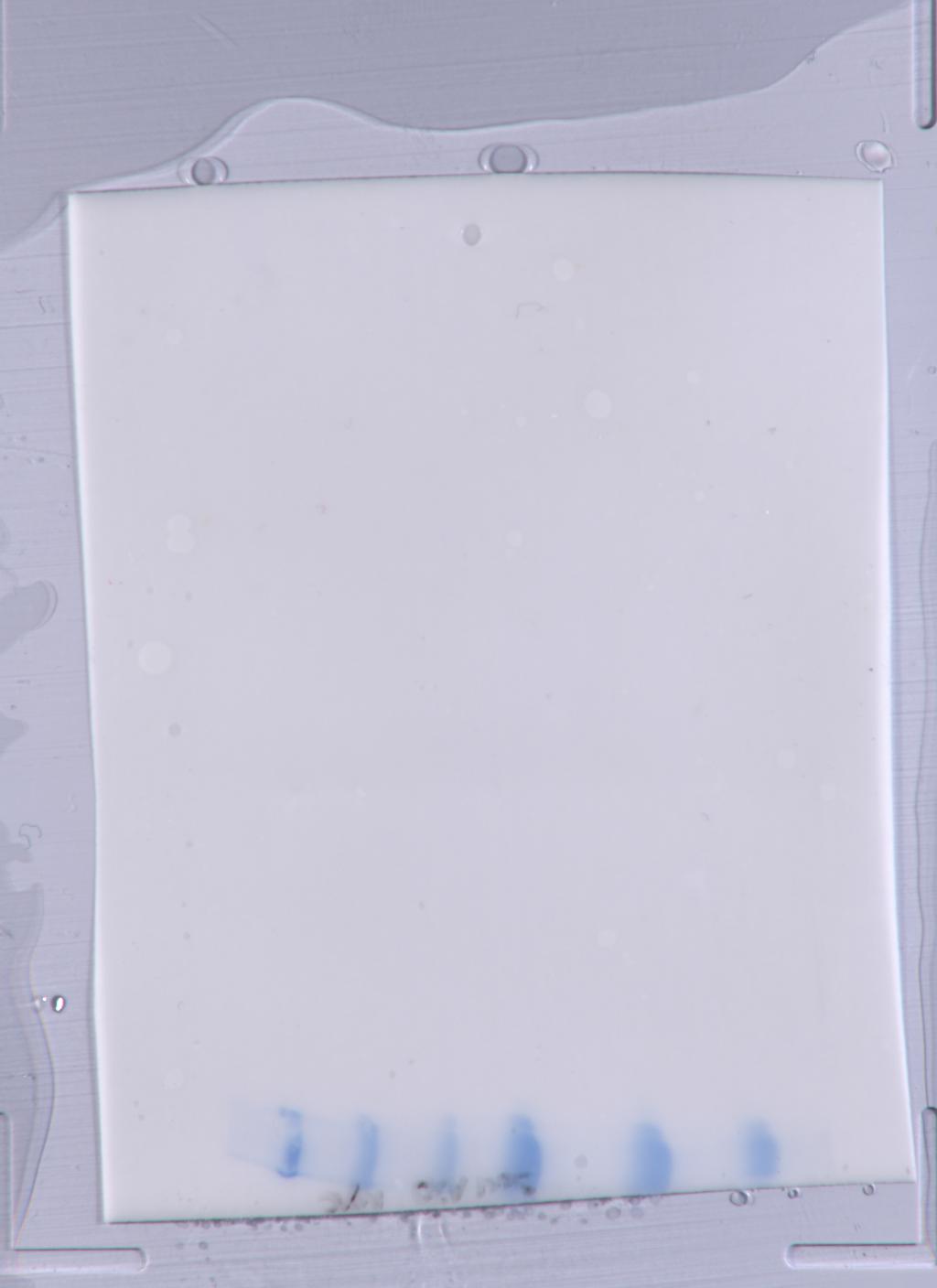

Supplement: Figure 1—figure supplement 1—source data 2. [file elife-80147-fig1-figsupp1-data2.zip › Figure 1- Figure Supplement 1- Source data 2/scc1aid 1513 2018.06.29_22.34.32-20_Ch-Marker.jpg]

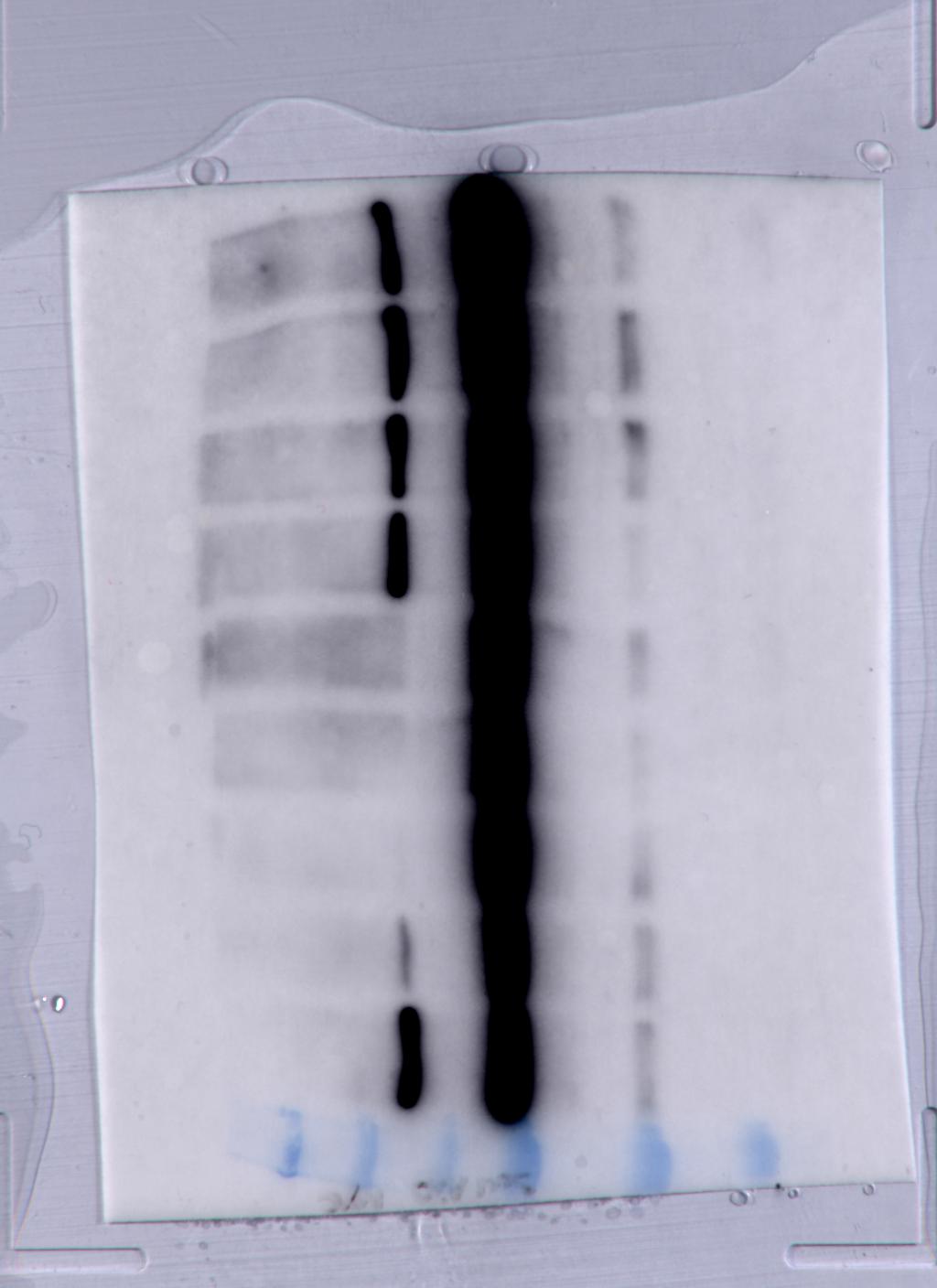

Supplement: Figure 1—figure supplement 1—source data 2. [file elife-80147-fig1-figsupp1-data2.zip › Figure 1- Figure Supplement 1- Source data 2/scc1aid 1513 2018.06.29_22.34.32-20_Ch+Marker.jpg]

$\alpha$ MYC

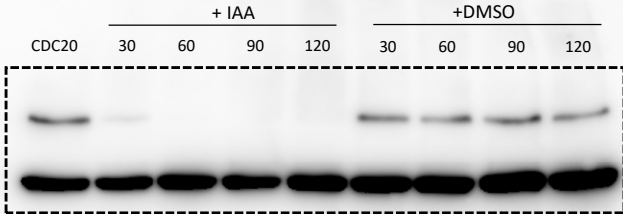

Supplement: Figure 1—figure supplement 1—source data 2. [file elife-80147-fig1-figsupp1-data2.zip › Figure 1- Figure Supplement 1- Source data 2/Figure 1_Figure_supplement_1-source_data_2.pdf]

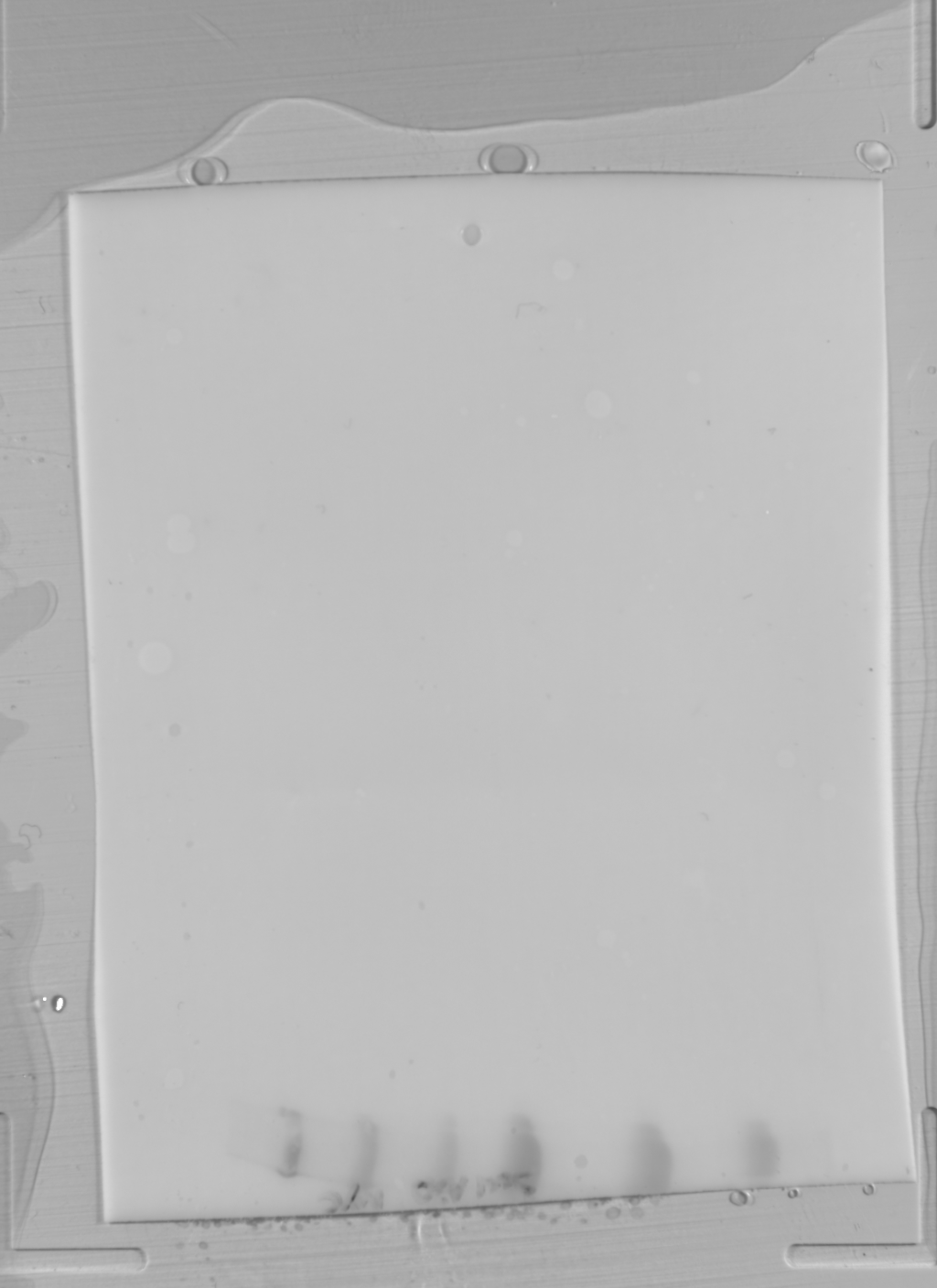

Supplement: Figure 1—figure supplement 1—source data 2. [file elife-80147-fig1-figsupp1-data2.zip › Figure 1- Figure Supplement 1- Source data 2/scc1aid 1513 2018.06.29_22.34.32-20_Ch-Marker.tif]

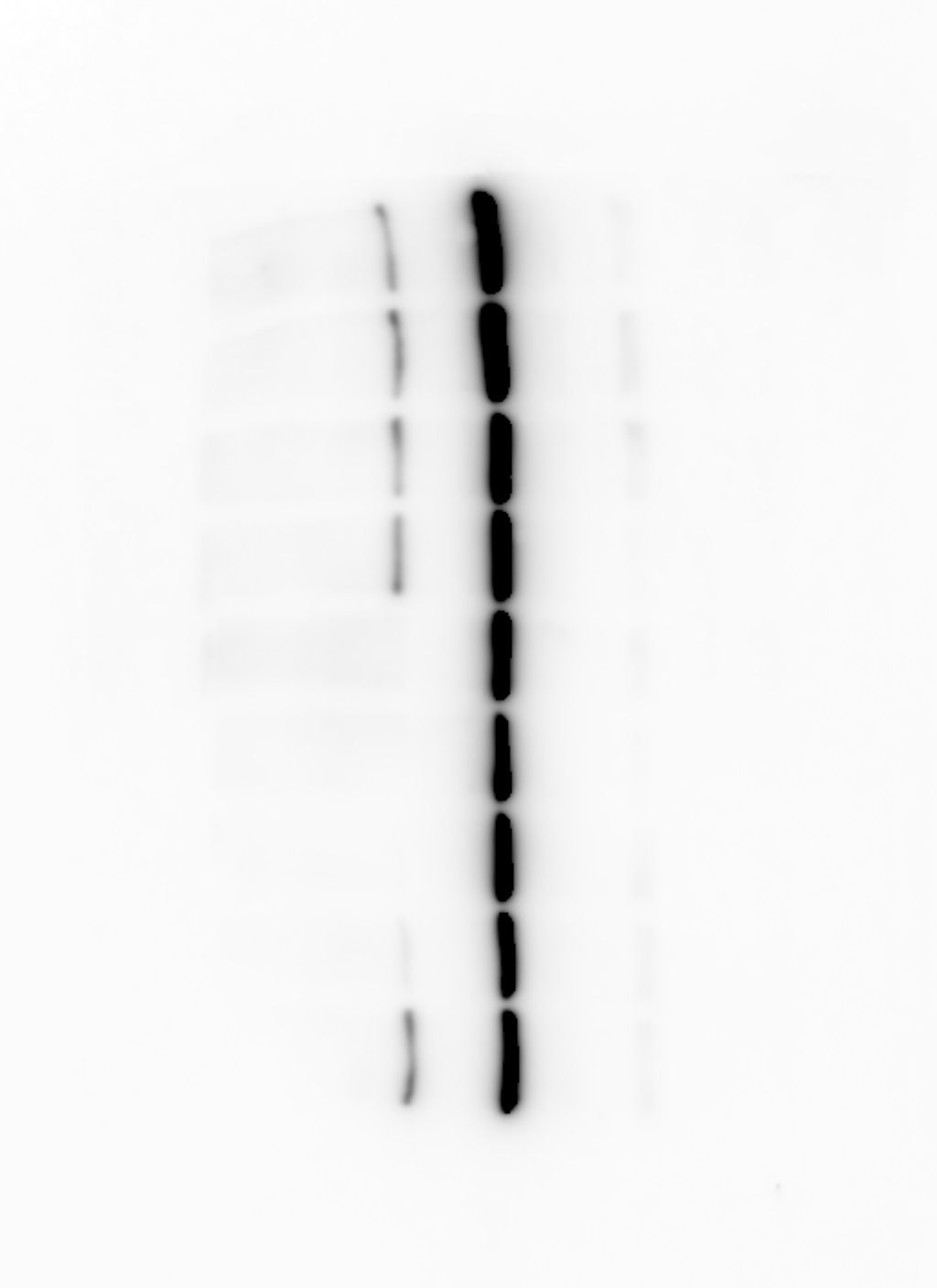

Supplement: Figure 1—figure supplement 1—source data 2. [file elife-80147-fig1-figsupp1-data2.zip › Figure 1- Figure Supplement 1- Source data 2/scc1aid 1513 2018.06.29_22.34.32-20_Ch.tif]

$\alpha$ HA

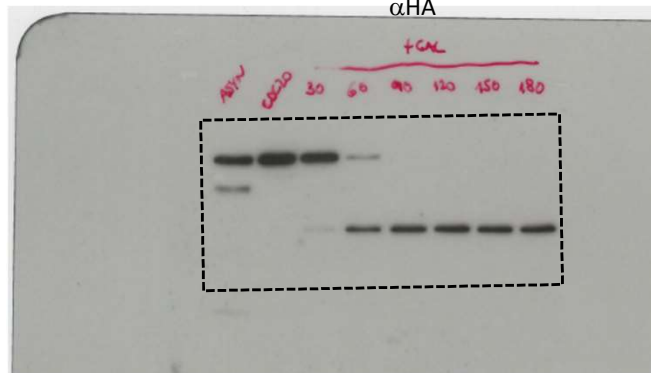

Supplement: Figure 1—figure supplement 2—source data 1. — 5 blots shown. [file elife-80147-fig1-figsupp2-data1.zip › Figure 1- Figure Supplement 2- Source data 1/Figure 1_Figure_supplement_2-_Source_data_1.pdf]

12/22/00  
30 60 90 120 150 180  
+GAL

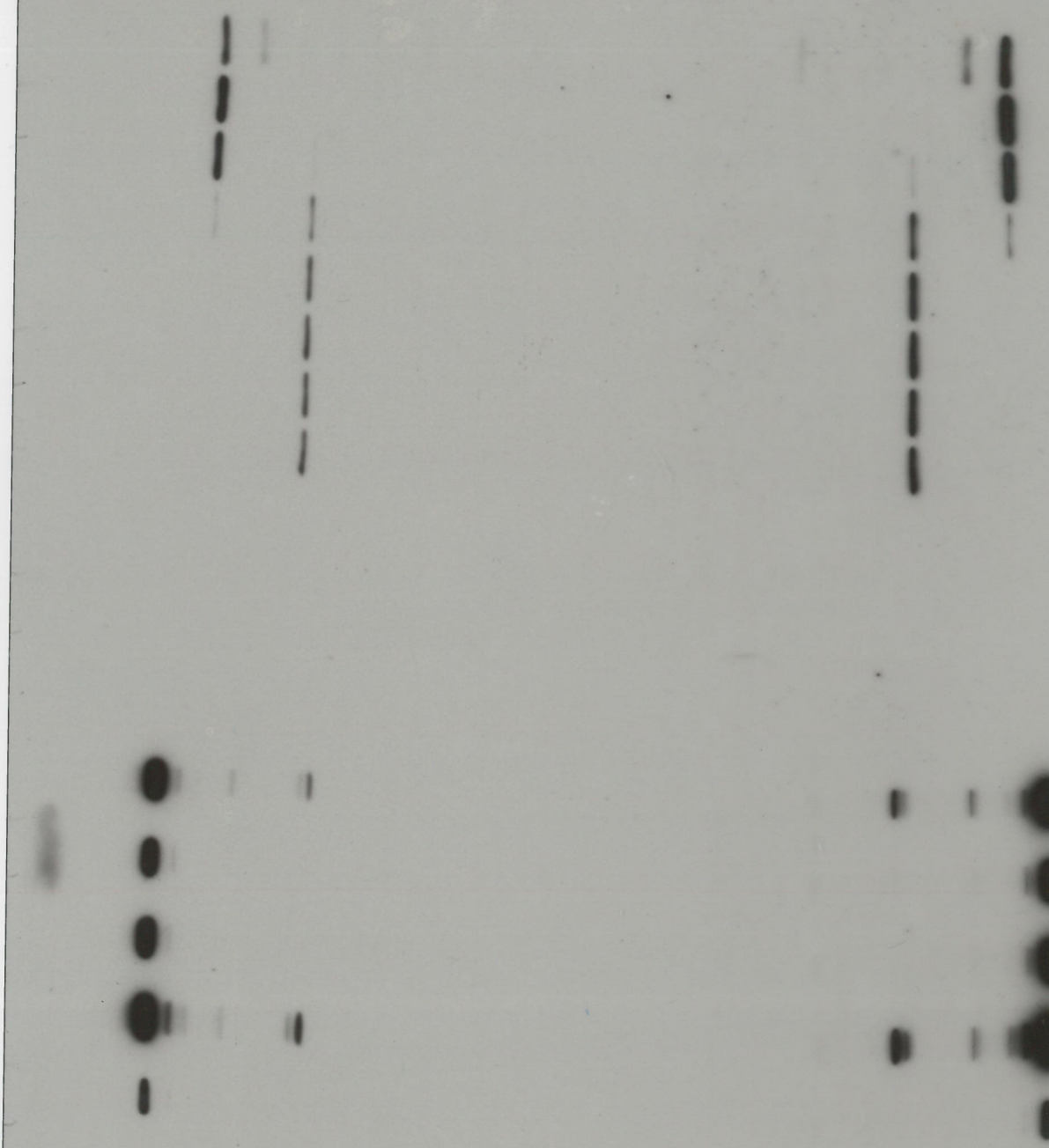

Supplement: Figure 1—figure supplement 2—source data 1. — 5 blots shown. [file elife-80147-fig1-figsupp2-data1.zip › Figure 1- Figure Supplement 2- Source data 1/2a WB TEVG nasmyth.pdf]

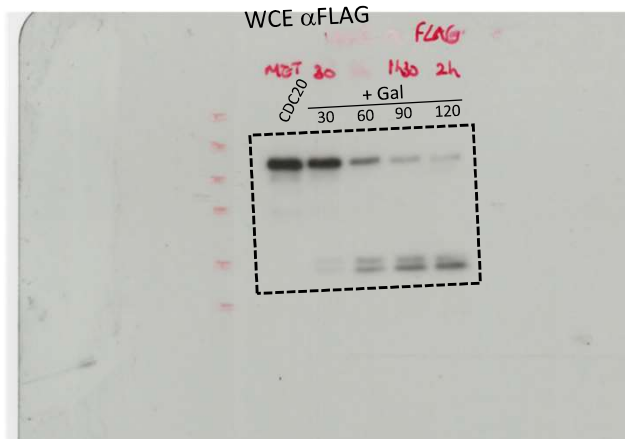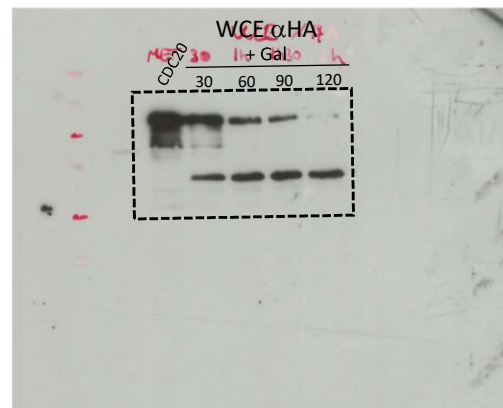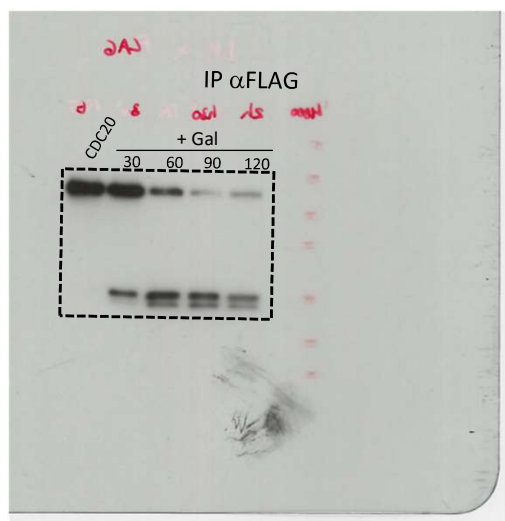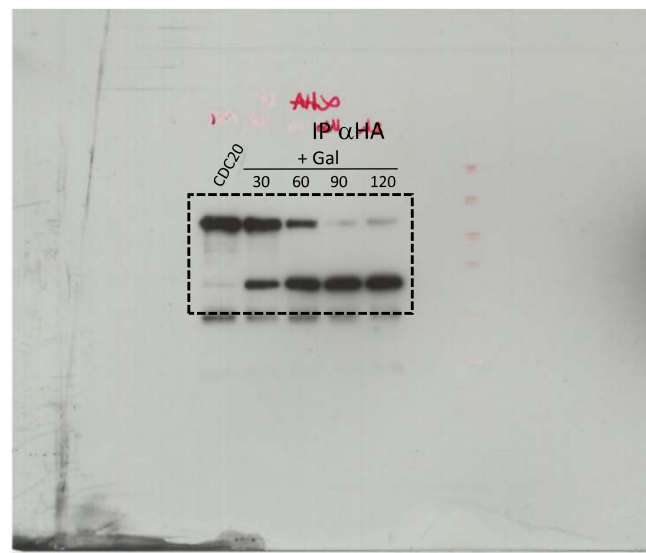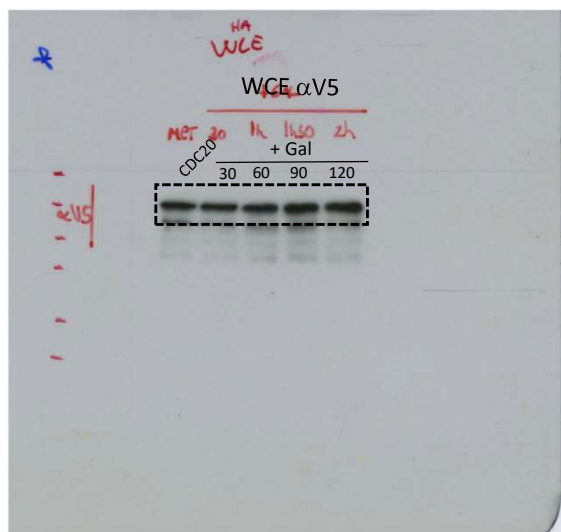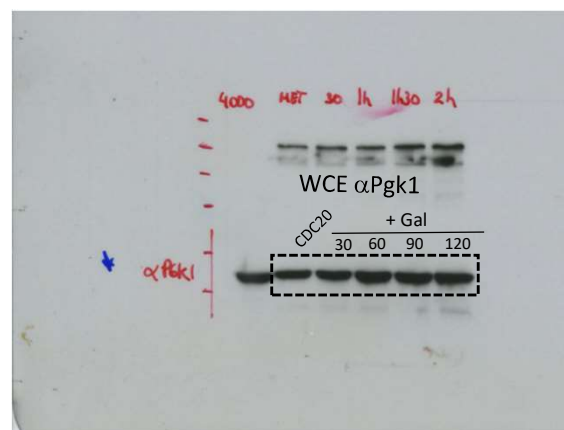

Supplement: Figure 2—source data 1. [file elife-80147-fig2-data1.zip › Figure 2- Source data 1/Figure_2_-Source_data_1.pdf]

IF  $\alpha$  V5

4000 2h 1h30 1h 30' NET

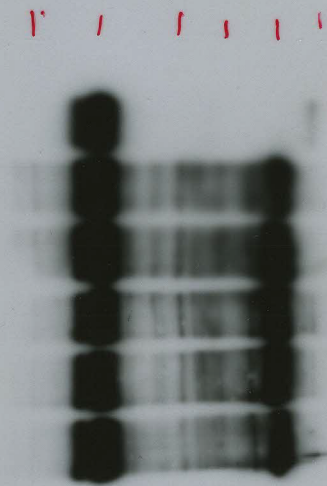

\*

<sup>H4</sup> VLE

+GAL

NET 30 1h 1h30 2h

$\alpha$  V5

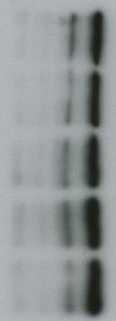

\*  $\alpha$  V5

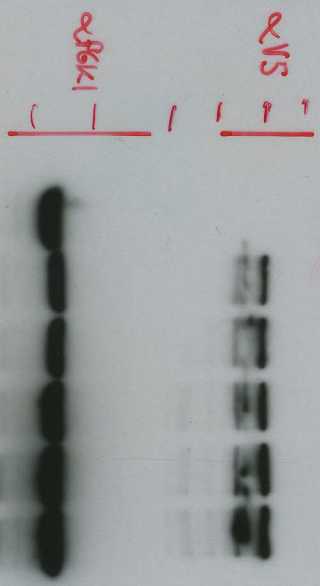

$\alpha$  V5

PLAS  
VLE

+GAL

NET 30 1h 1h30 2h

1 1 1 1 1 1

Supplement: Figure 2—source data 1. [file elife-80147-fig2-data1.zip › Figure 2- Source data 1/Fig 2_aV5.IP.pdf]

NCE  $\alpha$  FLAG  
MET 30 1h 1h30 2h

1 1 1 1 1

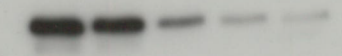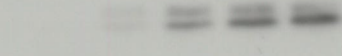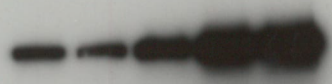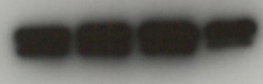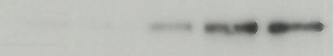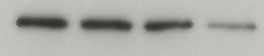

$\alpha$ HA IP  
2h 1h30 1h 30 MET

1 1 1 1 1

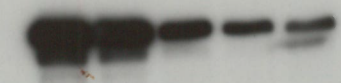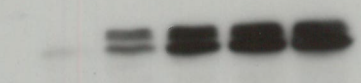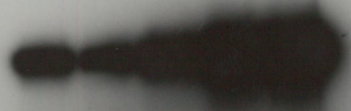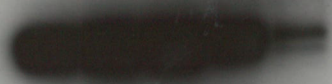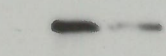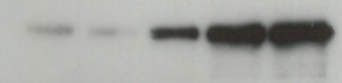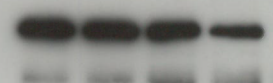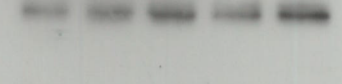

Supplement: Figure 2—source data 1. [file elife-80147-fig2-data1.zip › Figure 2- Source data 1/Fig 2_aFLAG.WCE_aHA.IP.pdf]

WCE K4A  
MET 30 1h 1h30 2h

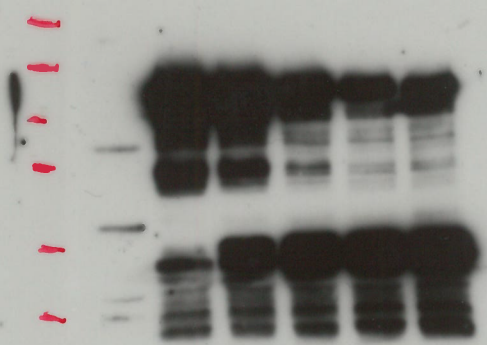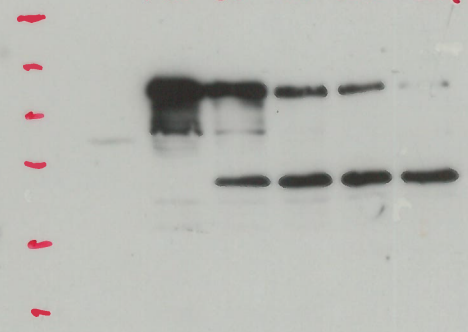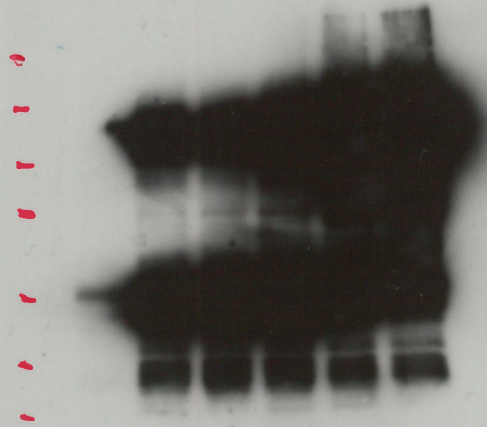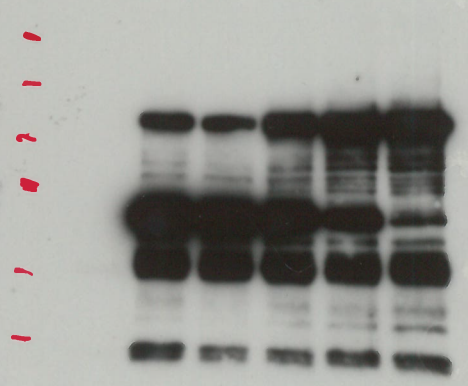

IP  $\alpha$  FLAG

4000 2h 1h30 1h 30' MET

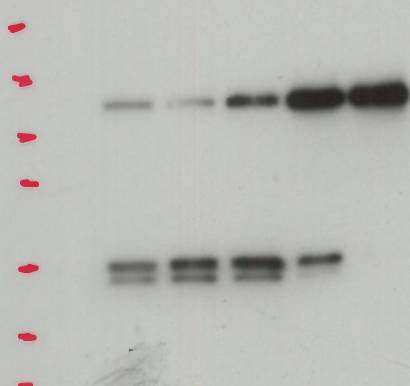

Supplement: Figure 2—source data 1. [file elife-80147-fig2-data1.zip › Figure 2- Source data 1/Fig 2_aFLAG.IP_aHA.WCE.pdf]

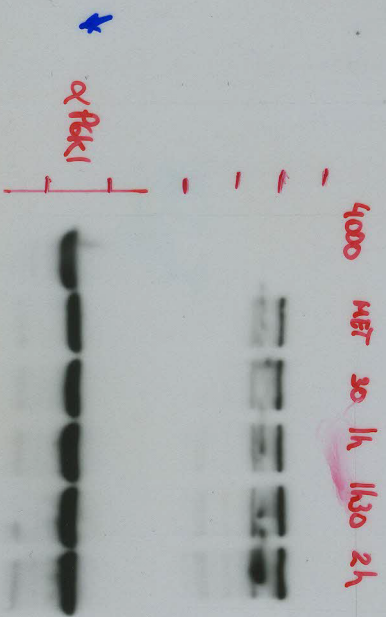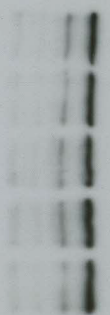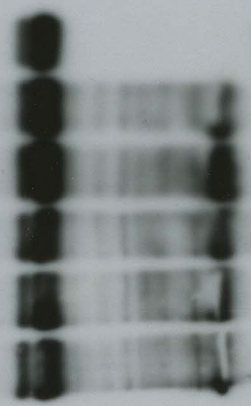

Supplement: Figure 2—source data 1. [file elife-80147-fig2-data1.zip › Figure 2- Source data 1/Fig 2_aPgk1.pdf]

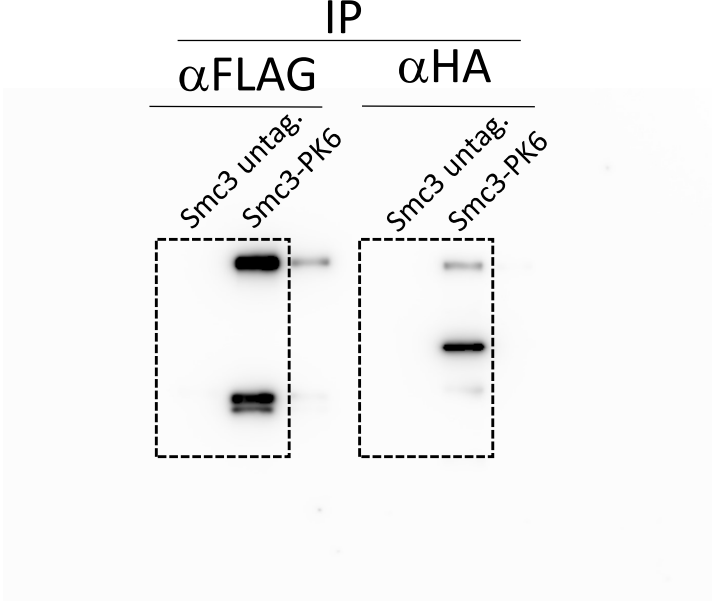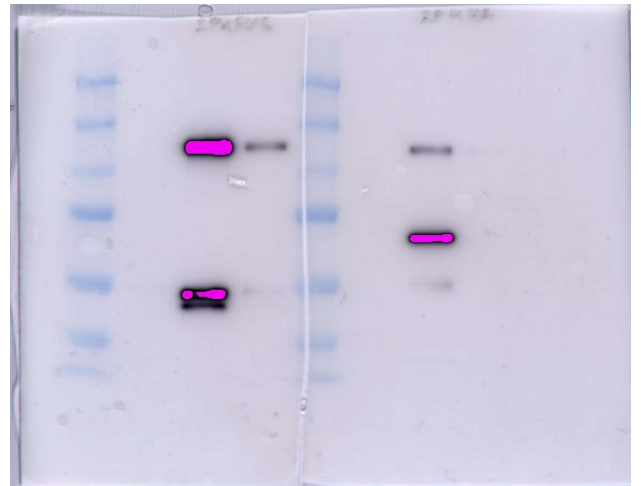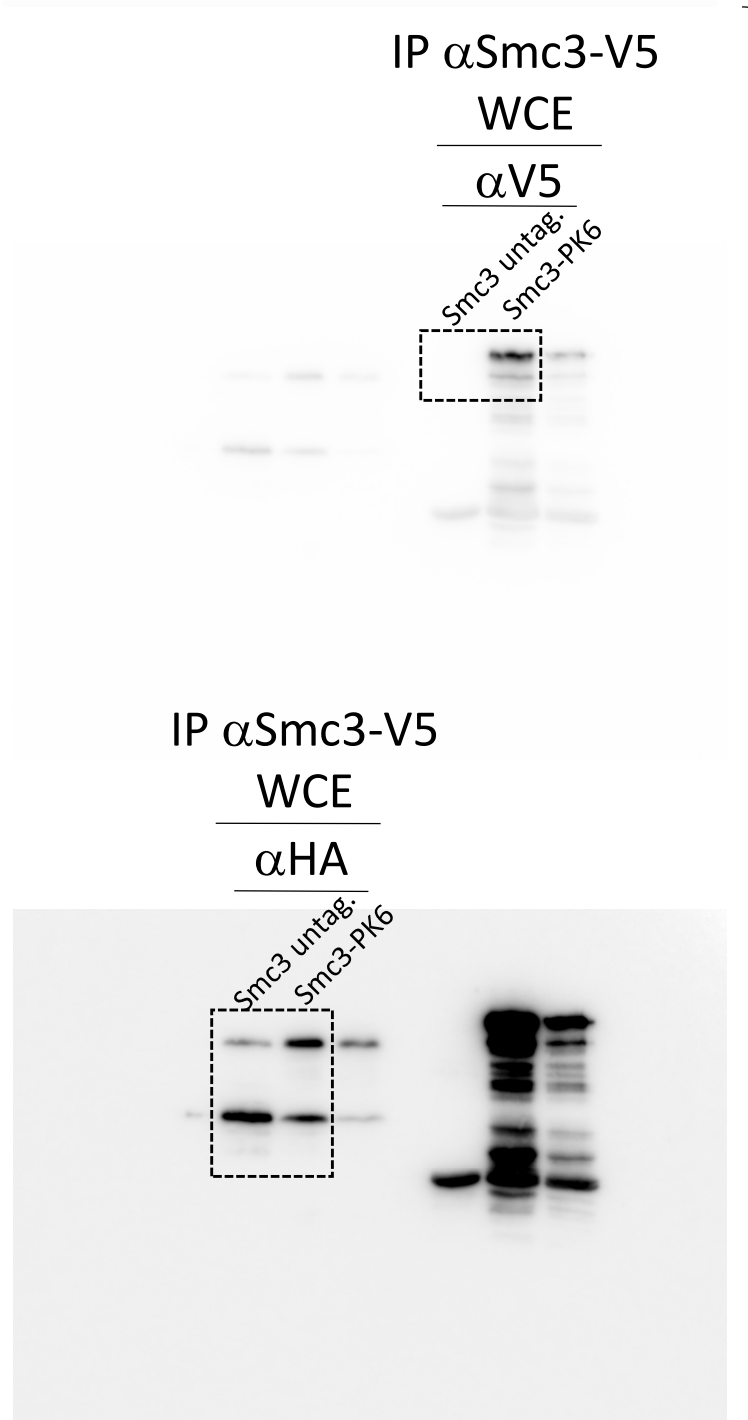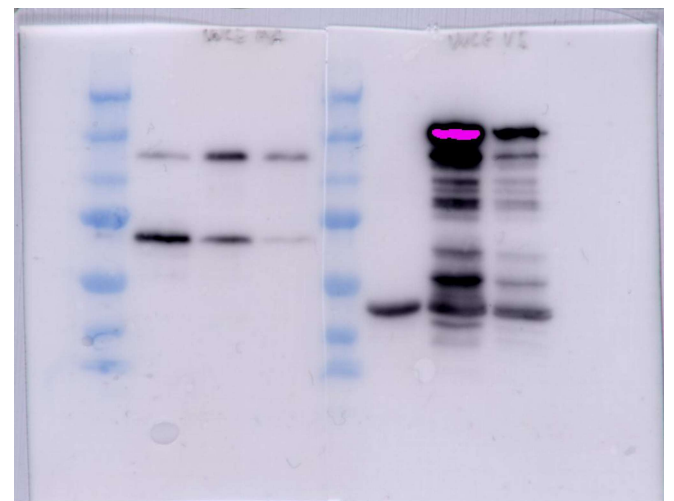

Supplement: Figure 2—figure supplement 1—source data 1. [file elife-80147-fig2-figsupp1-data1.zip › Figure 2- Figure Supplement 1- Source data 1/Figure 2_Figure_supplement_1-_source_data_1.pdf]

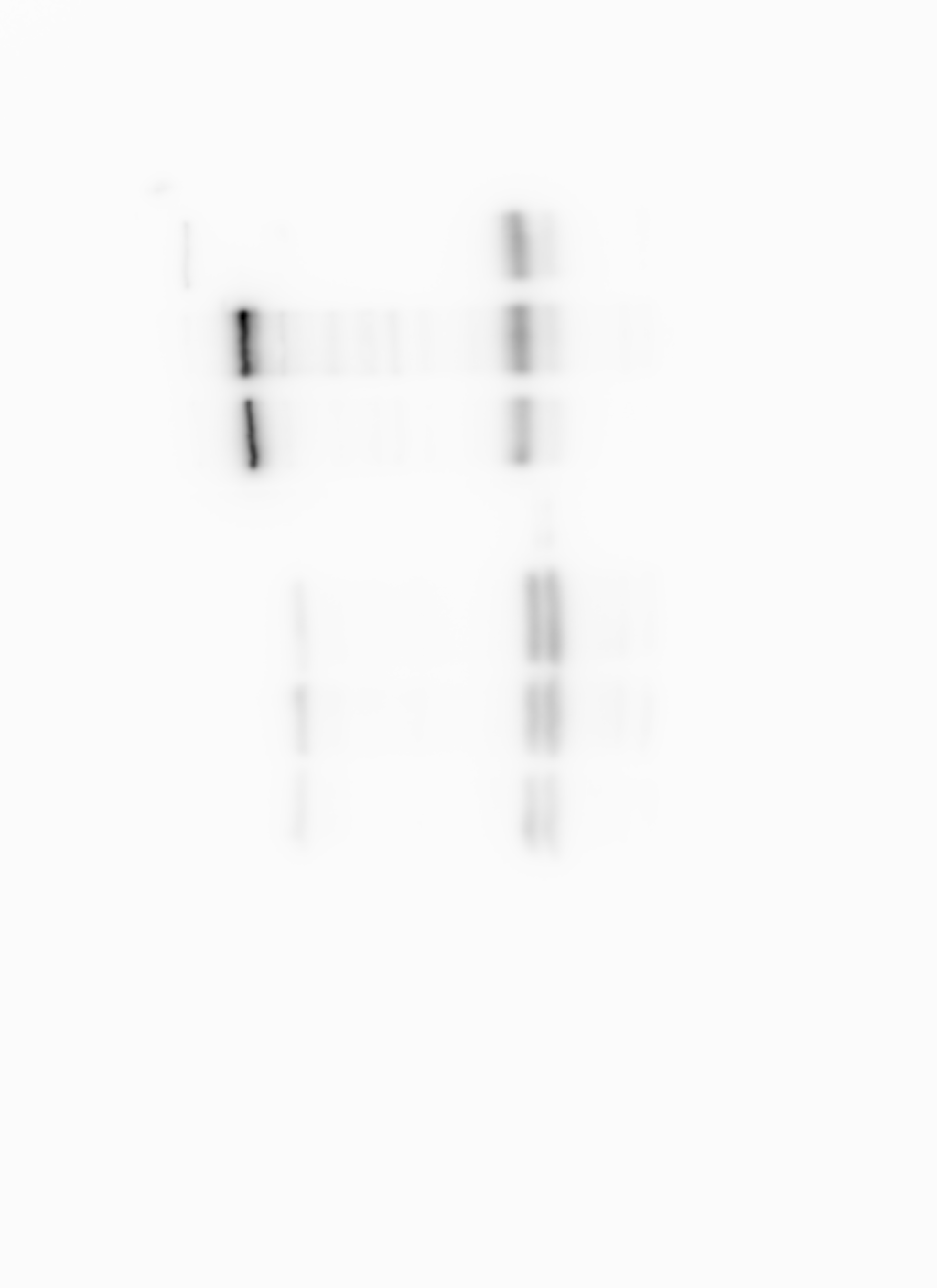

Supplement: Figure 2—figure supplement 1—source data 1. [file elife-80147-fig2-figsupp1-data1.zip › Figure 2- Figure Supplement 1- Source data 1/aV5.IP/IP V5 WCE Flag 2019.09.13_14.26.17-01_Ch.tif]

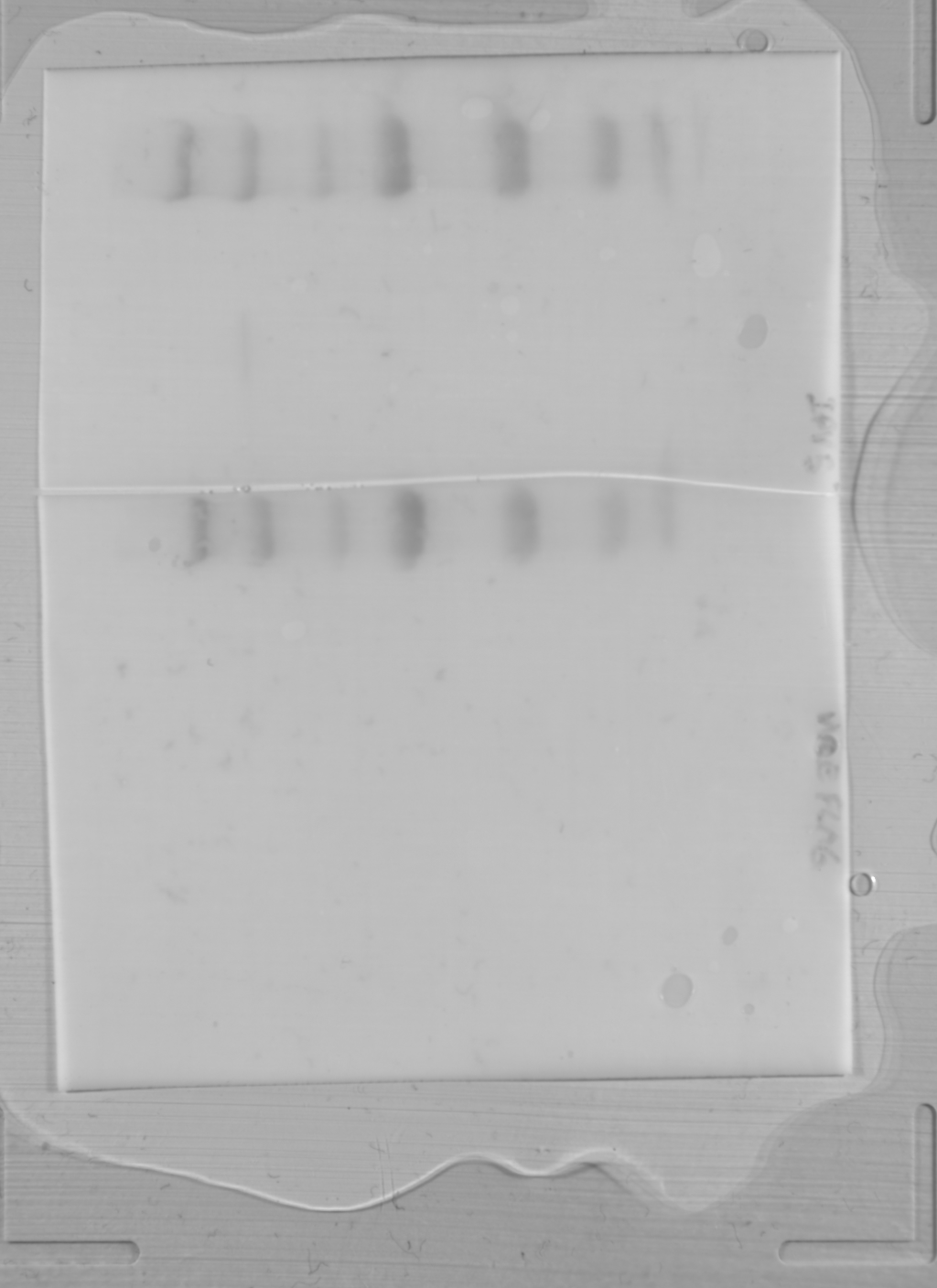

Supplement: Figure 2—figure supplement 1—source data 1. [file elife-80147-fig2-figsupp1-data1.zip › Figure 2- Figure Supplement 1- Source data 1/aV5.IP/IP V5 WCE Flag 2019.09.13_14.26.17-01_Ch-Marker.tif]

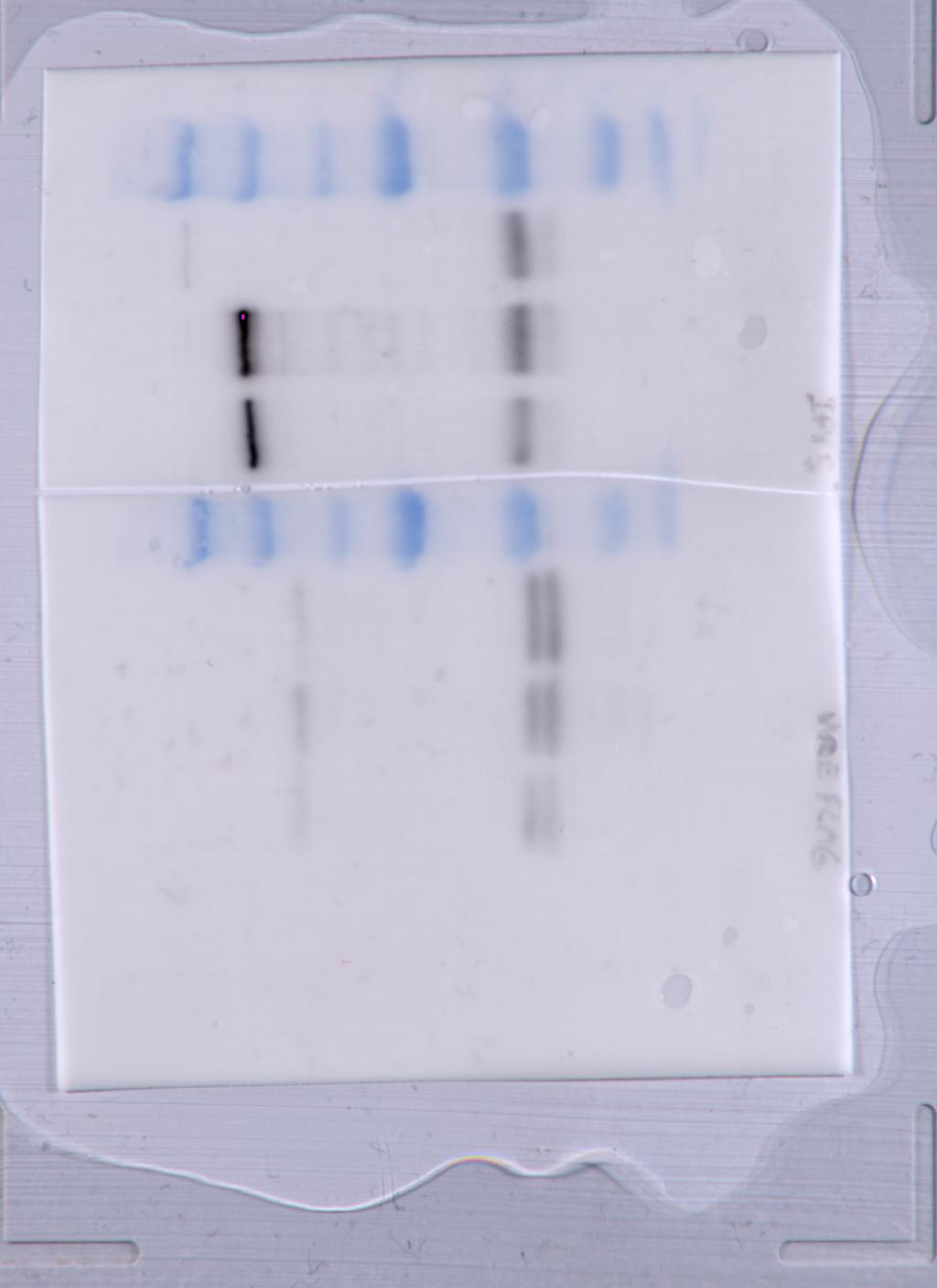

Supplement: Figure 2—figure supplement 1—source data 1. [file elife-80147-fig2-figsupp1-data1.zip › Figure 2- Figure Supplement 1- Source data 1/aV5.IP/IP V5 WCE Flag 2019.09.13_14.26.17-01_Ch+Marker.jpg]

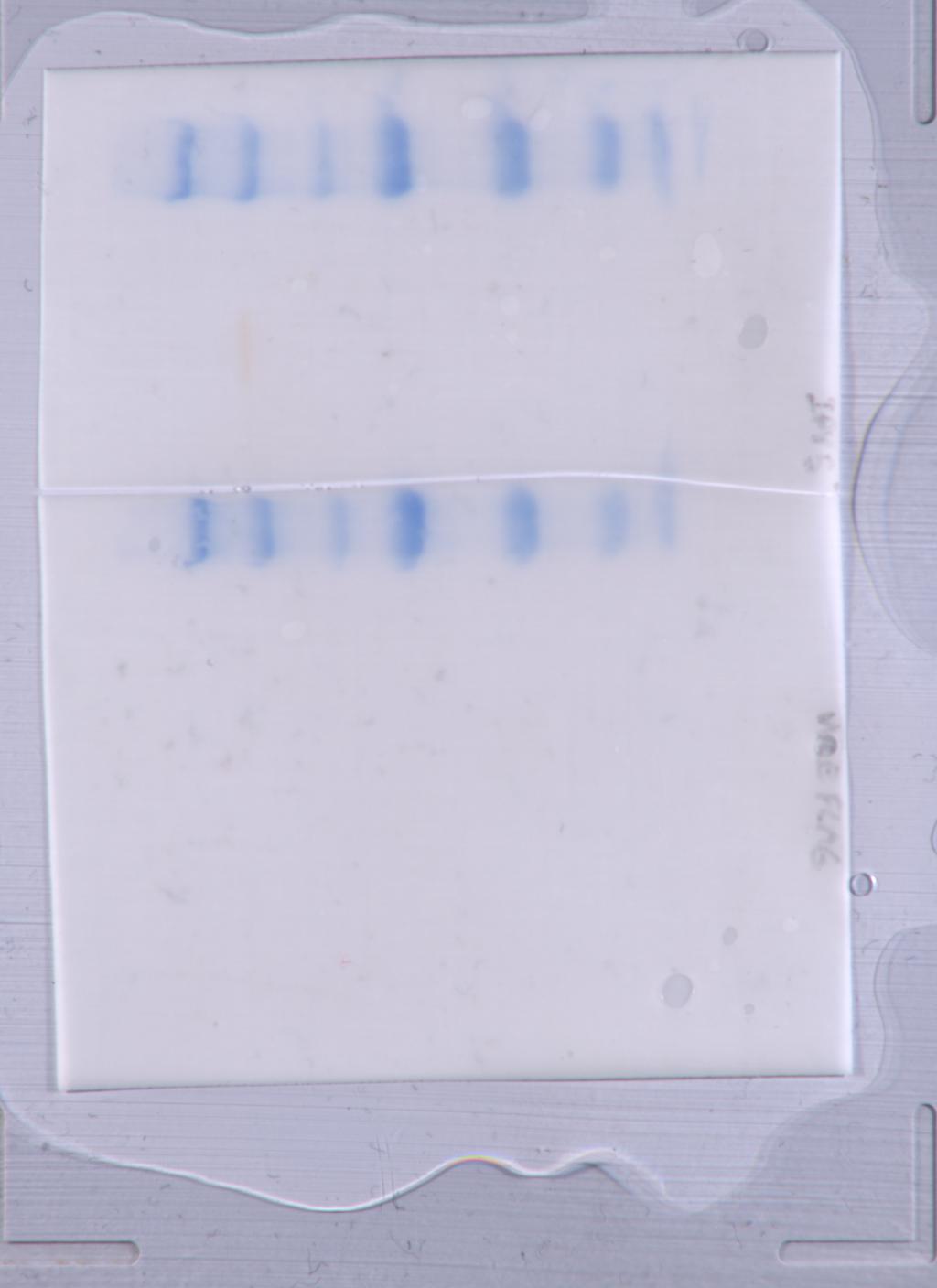

Supplement: Figure 2—figure supplement 1—source data 1. [file elife-80147-fig2-figsupp1-data1.zip › Figure 2- Figure Supplement 1- Source data 1/aV5.IP/IP V5 WCE Flag 2019.09.13_14.26.17-01_Ch-Marker.jpg]

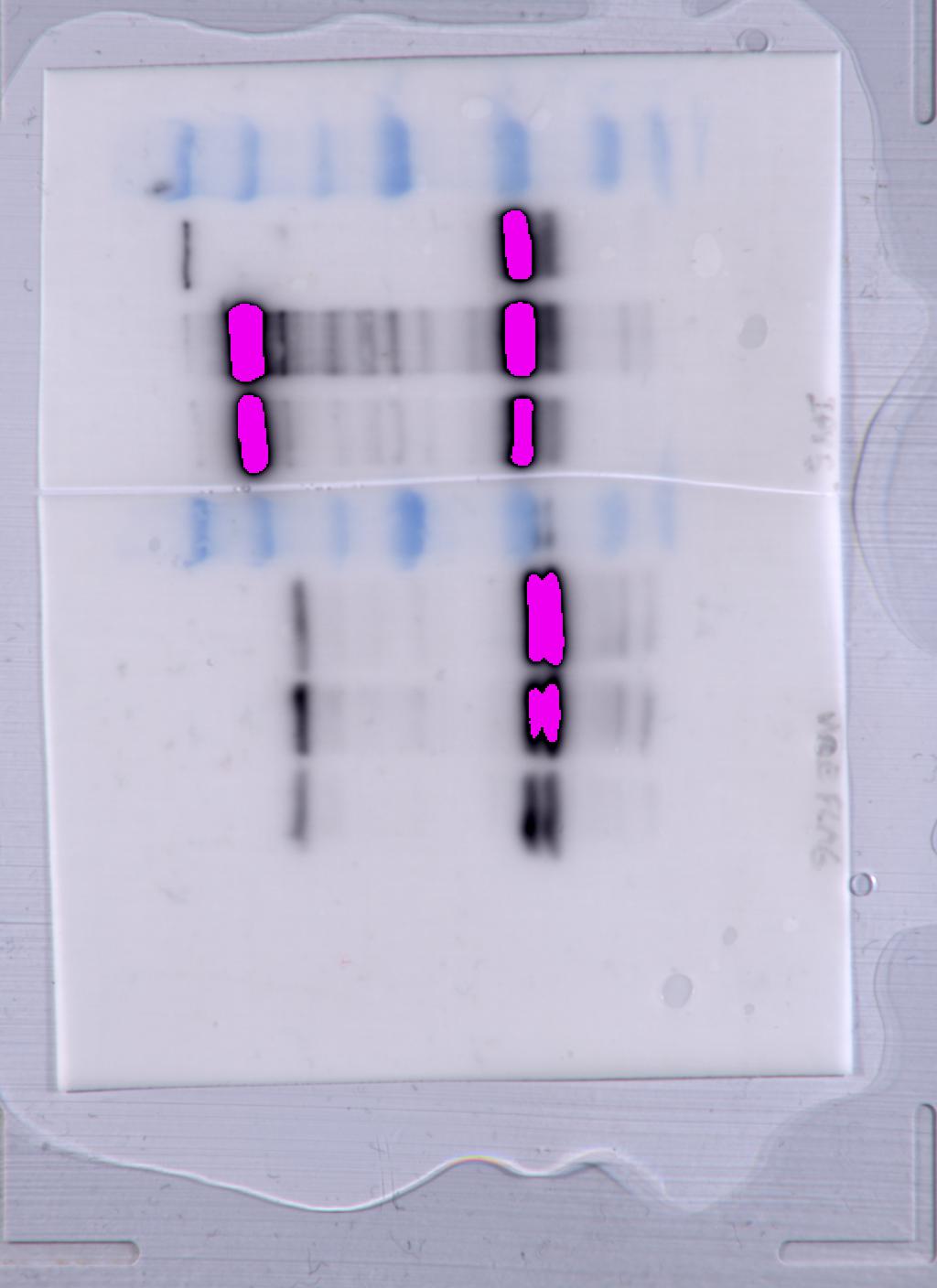

Supplement: Figure 2—figure supplement 1—source data 1. [file elife-80147-fig2-figsupp1-data1.zip › Figure 2- Figure Supplement 1- Source data 1/aFLAG.WCE/IP V5 WCE Flag 2019.09.13_14.26.17-07_Ch+Marker.jpg]

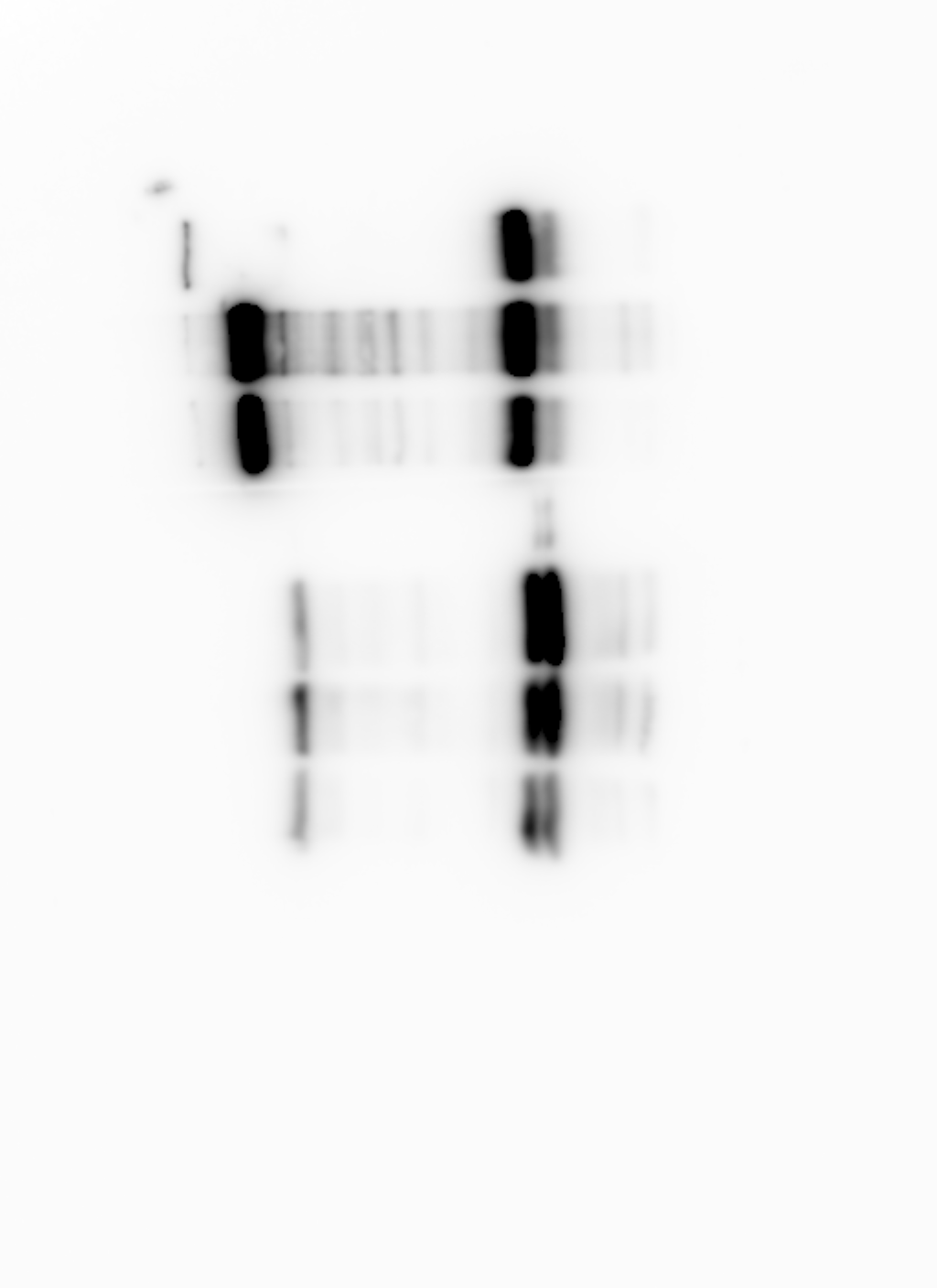

Supplement: Figure 2—figure supplement 1—source data 1. [file elife-80147-fig2-figsupp1-data1.zip › Figure 2- Figure Supplement 1- Source data 1/aFLAG.WCE/IP V5 WCE Flag 2019.09.13_14.26.17-07_Ch.tif]

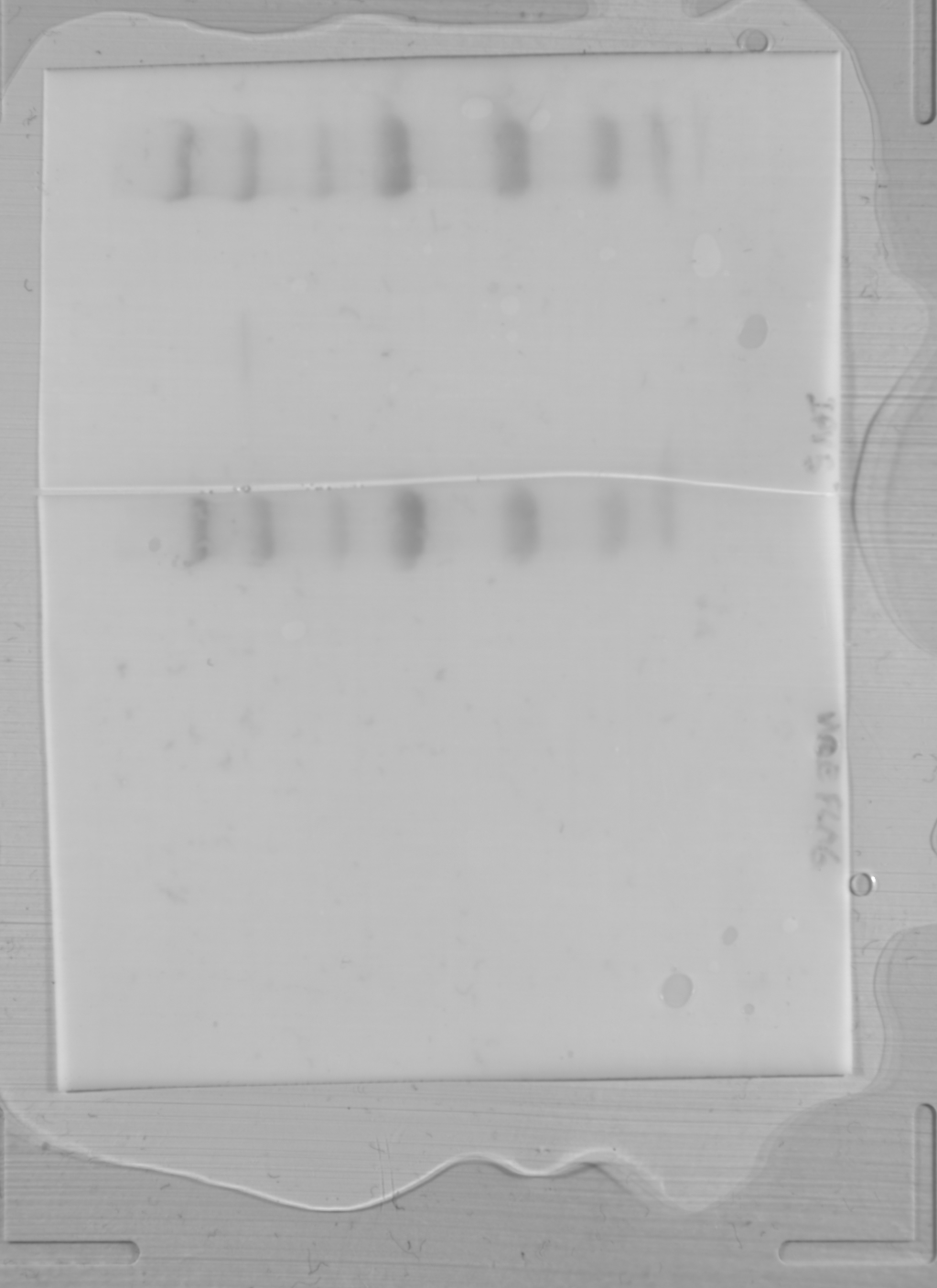

Supplement: Figure 2—figure supplement 1—source data 1. [file elife-80147-fig2-figsupp1-data1.zip › Figure 2- Figure Supplement 1- Source data 1/aFLAG.WCE/IP V5 WCE Flag 2019.09.13_14.26.17-07_Ch-Marker.tif]

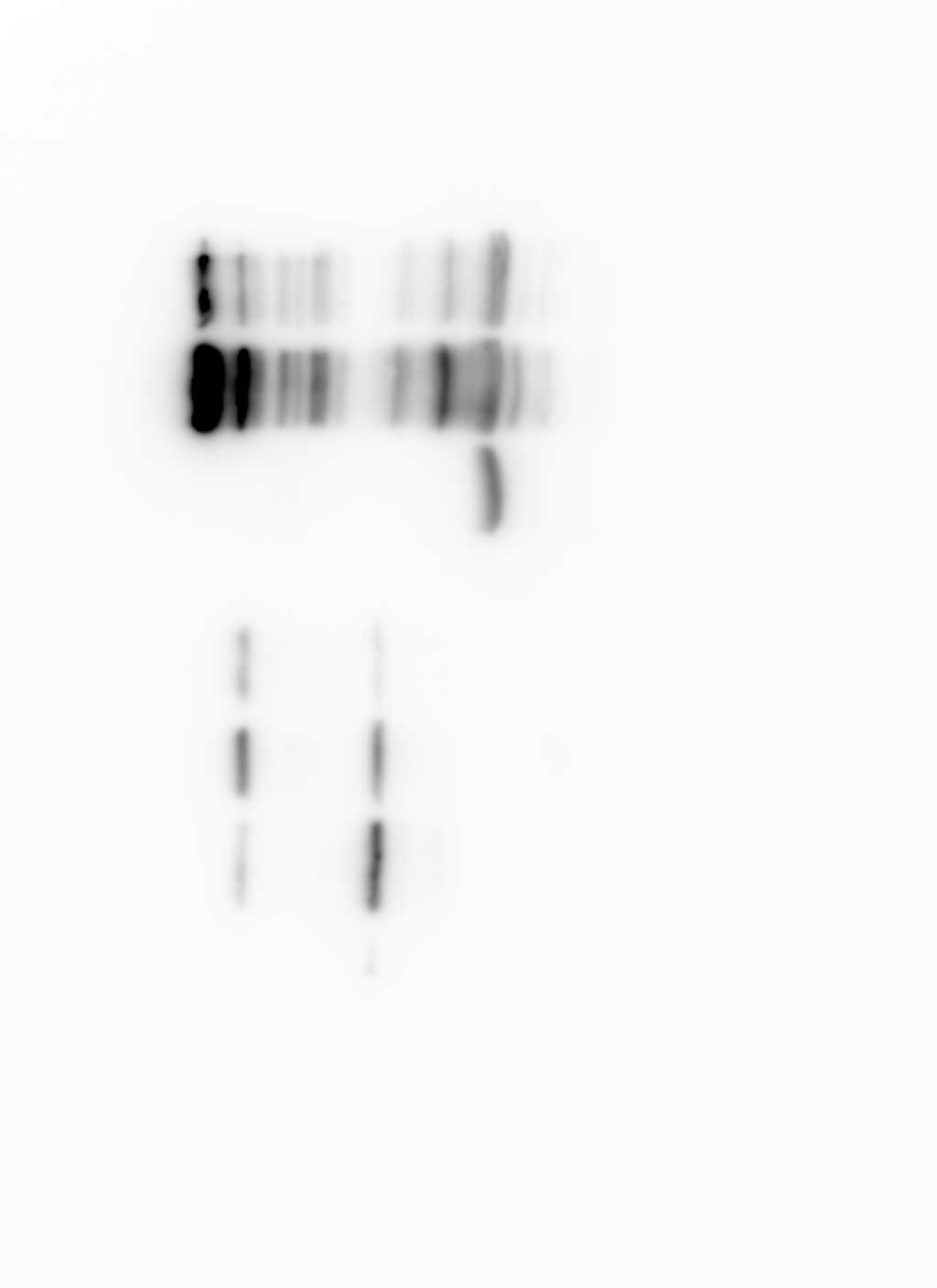

Supplement: Figure 2—figure supplement 1—source data 1. [file elife-80147-fig2-figsupp1-data1.zip › Figure 2- Figure Supplement 1- Source data 1/aV5.HA.WCE/WCE HA & V5 2019.09.13_14.22.01-10_Ch.tif]

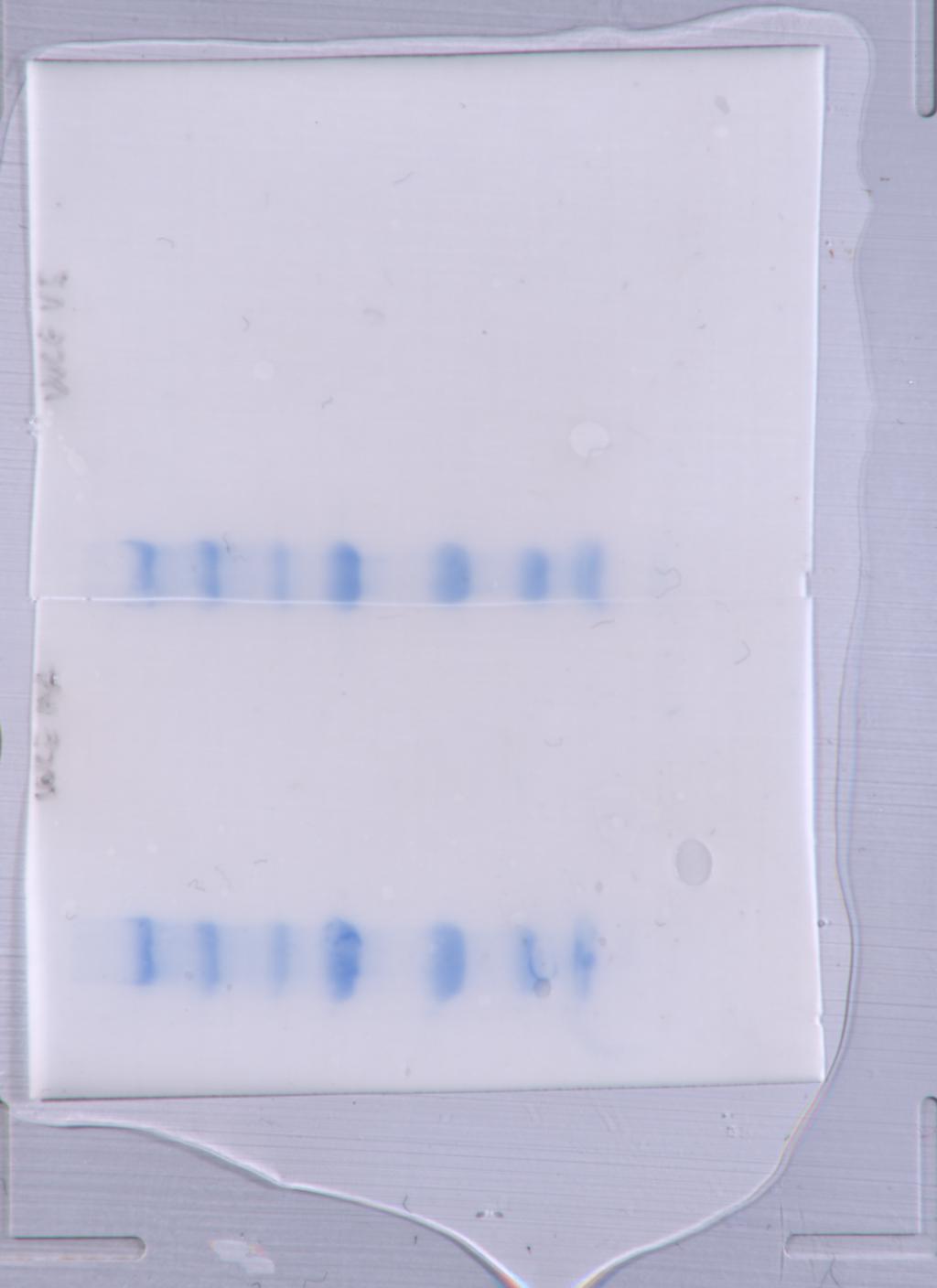

Supplement: Figure 2—figure supplement 1—source data 1. [file elife-80147-fig2-figsupp1-data1.zip › Figure 2- Figure Supplement 1- Source data 1/aV5.HA.WCE/WCE HA & V5 2019.09.13_14.22.01-10_Ch-Marker.jpg]

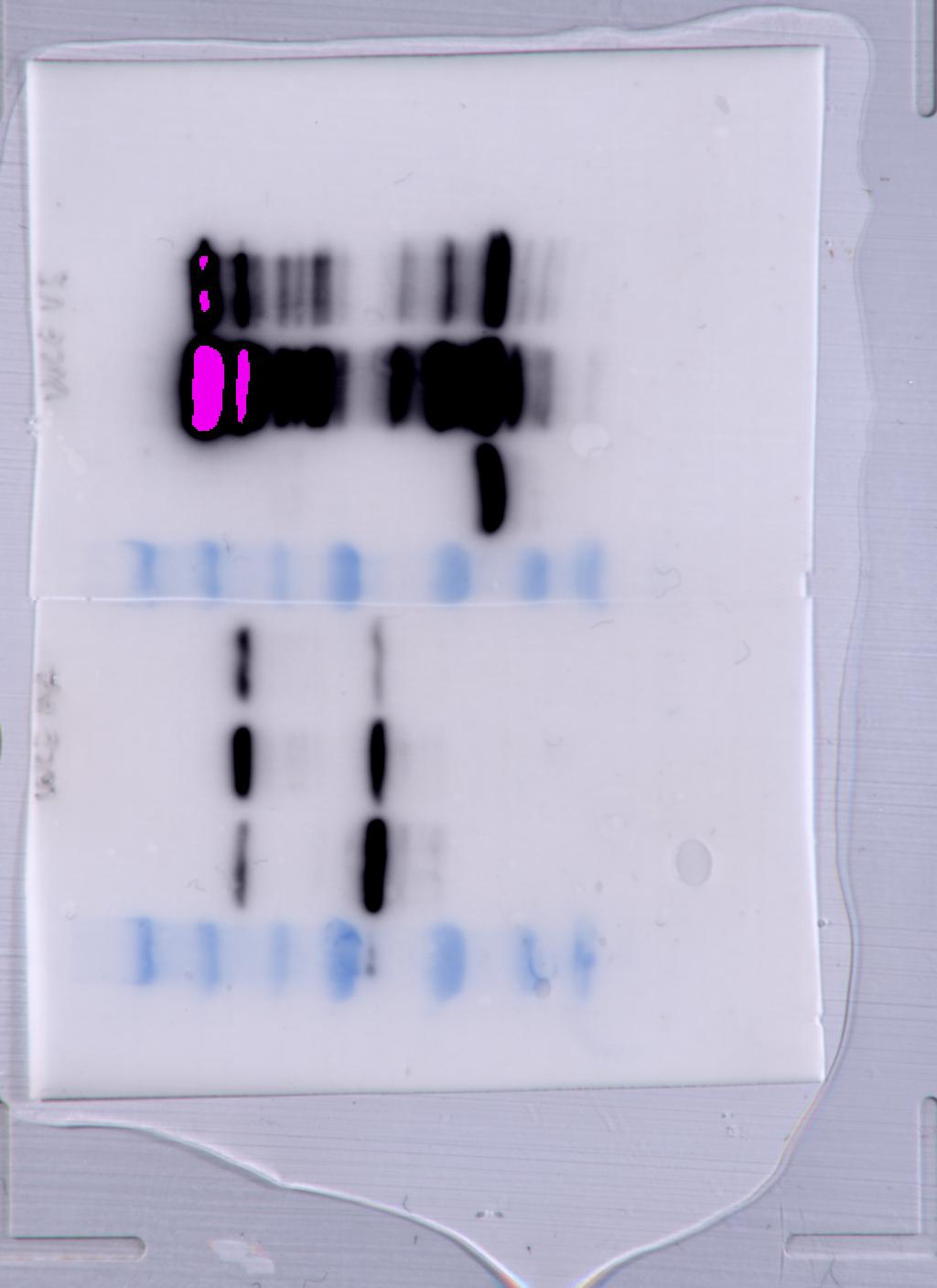

Supplement: Figure 2—figure supplement 1—source data 1. [file elife-80147-fig2-figsupp1-data1.zip › Figure 2- Figure Supplement 1- Source data 1/aV5.HA.WCE/WCE HA & V5 2019.09.13_14.22.01-10_Ch+Marker.jpg]

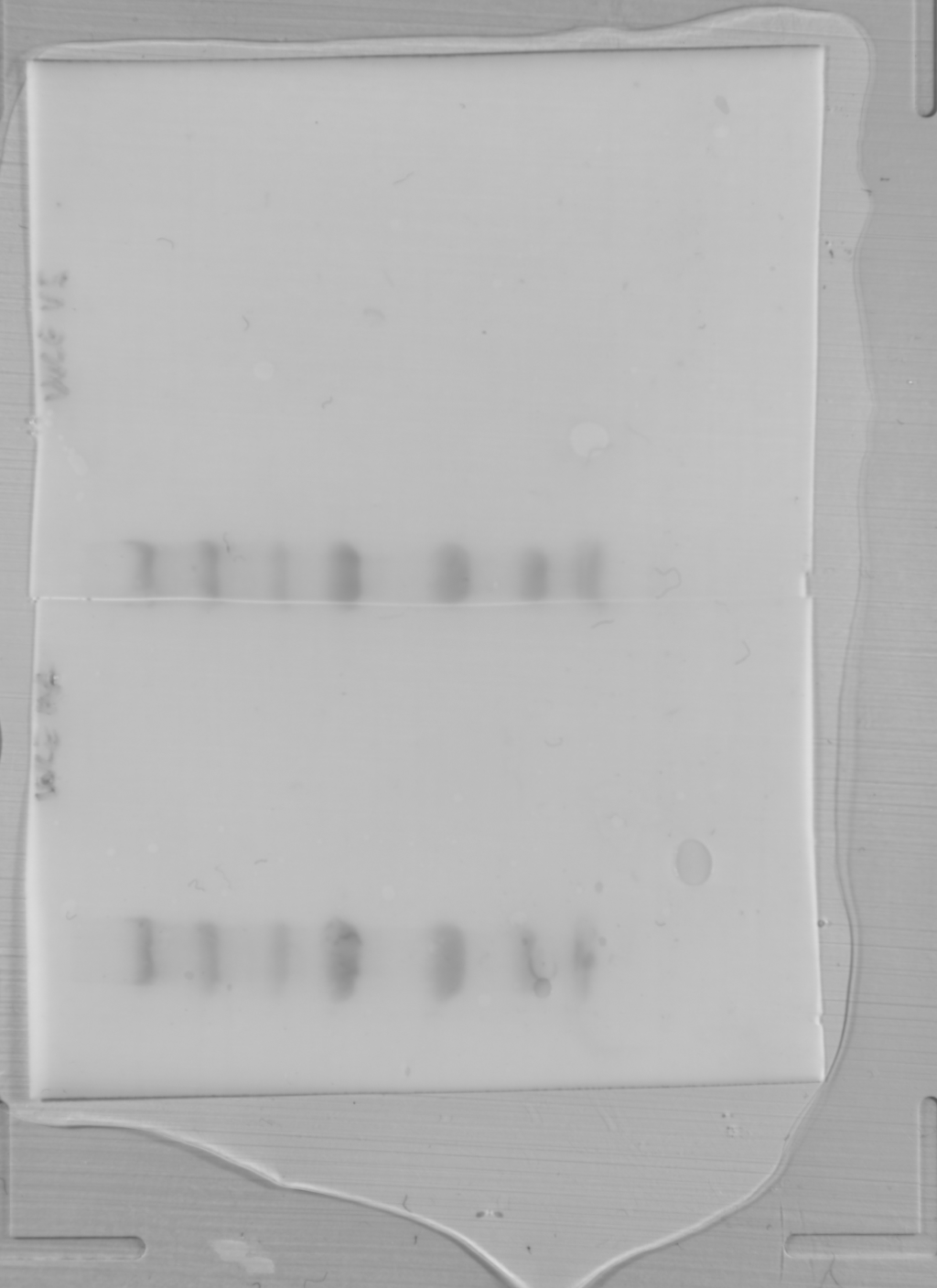

Supplement: Figure 2—figure supplement 1—source data 1. [file elife-80147-fig2-figsupp1-data1.zip › Figure 2- Figure Supplement 1- Source data 1/aV5.HA.WCE/WCE HA & V5 2019.09.13_14.22.01-10_Ch-Marker.tif]

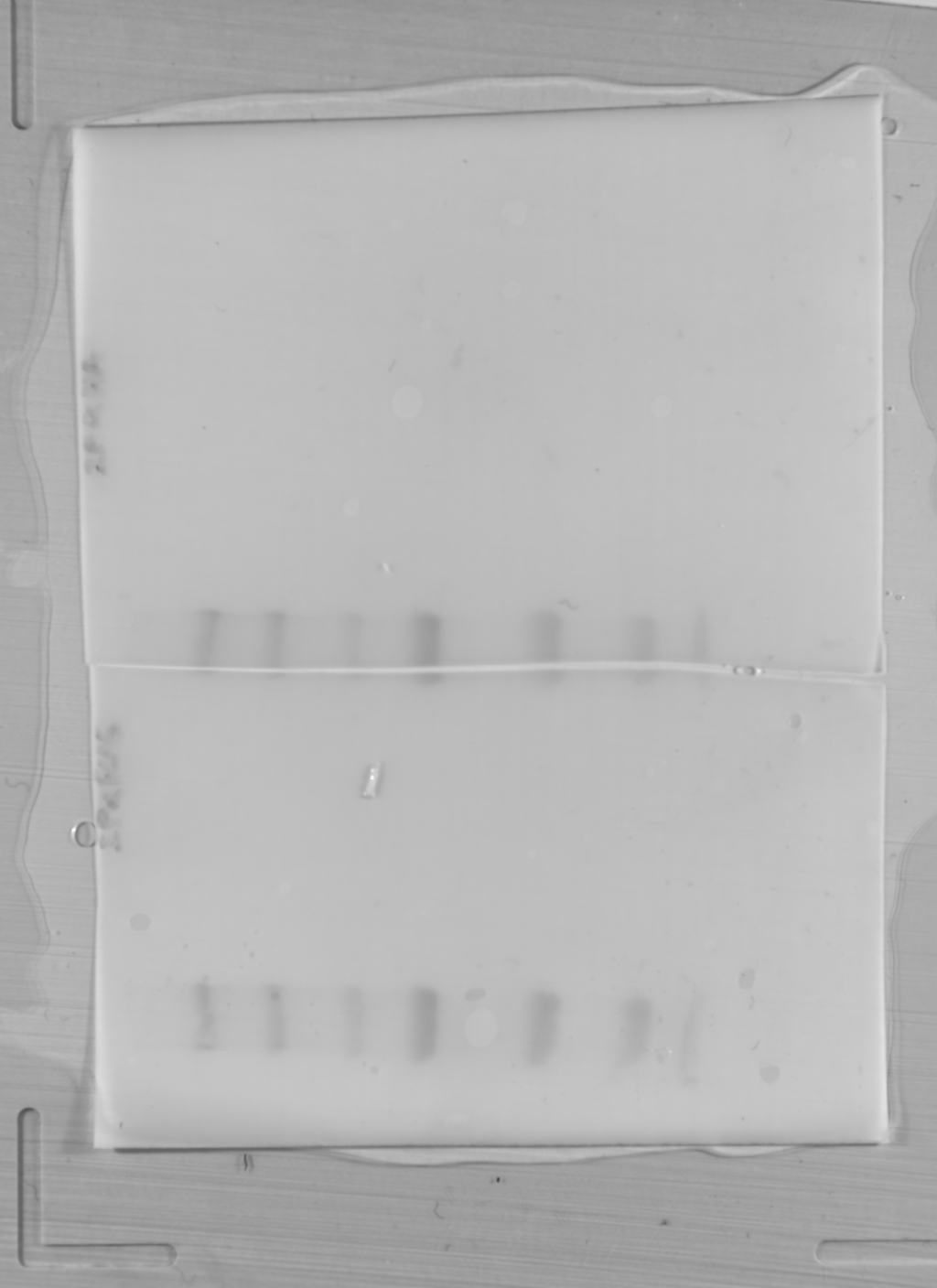

Supplement: Figure 2—figure supplement 1—source data 1. [file elife-80147-fig2-figsupp1-data1.zip › Figure 2- Figure Supplement 1- Source data 1/aFLAG.IP_aHA.IP/IPv5 aFLAG &HA 2019.09.13_14.18.25-03_Ch-Marker.tif]

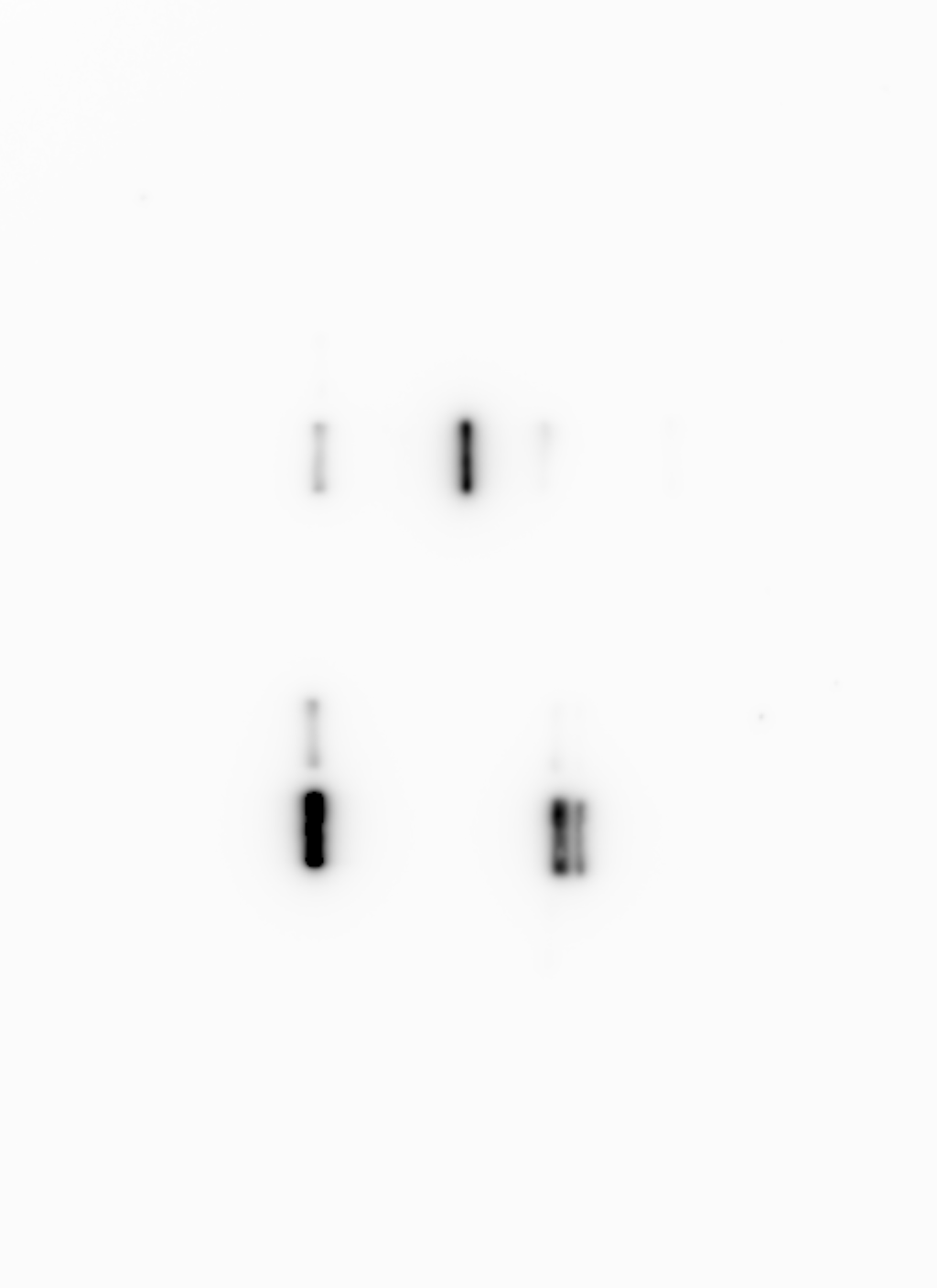

Supplement: Figure 2—figure supplement 1—source data 1. [file elife-80147-fig2-figsupp1-data1.zip › Figure 2- Figure Supplement 1- Source data 1/aFLAG.IP_aHA.IP/IPv5 aFLAG &HA 2019.09.13_14.18.25-03_Ch.tif]

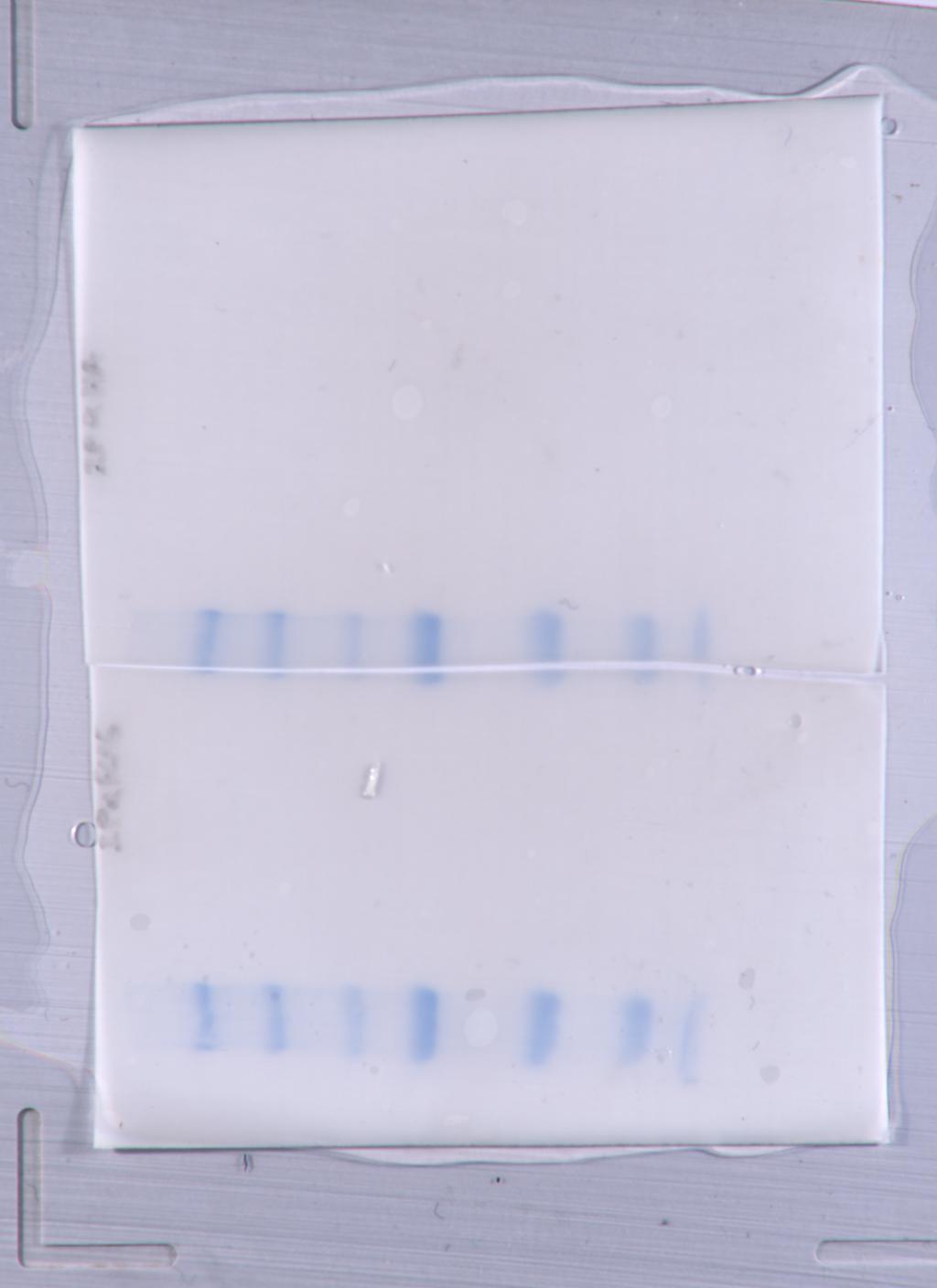

Supplement: Figure 2—figure supplement 1—source data 1. [file elife-80147-fig2-figsupp1-data1.zip › Figure 2- Figure Supplement 1- Source data 1/aFLAG.IP_aHA.IP/IPv5 aFLAG &HA 2019.09.13_14.18.25-03_Ch-Marker.jpg]

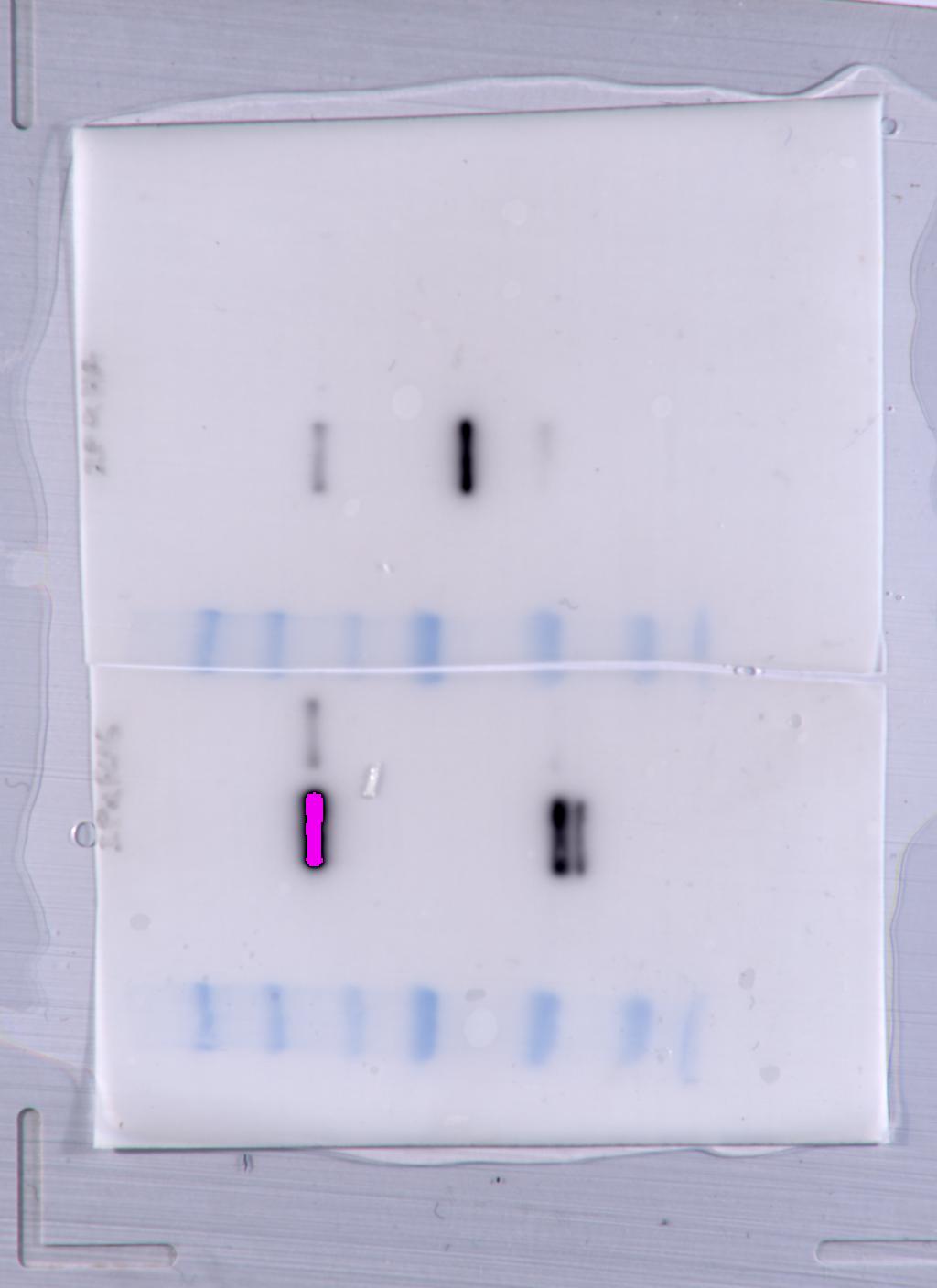

Supplement: Figure 2—figure supplement 1—source data 1. [file elife-80147-fig2-figsupp1-data1.zip › Figure 2- Figure Supplement 1- Source data 1/aFLAG.IP_aHA.IP/IPv5 aFLAG &HA 2019.09.13_14.18.25-03_Ch+Marker.jpg]

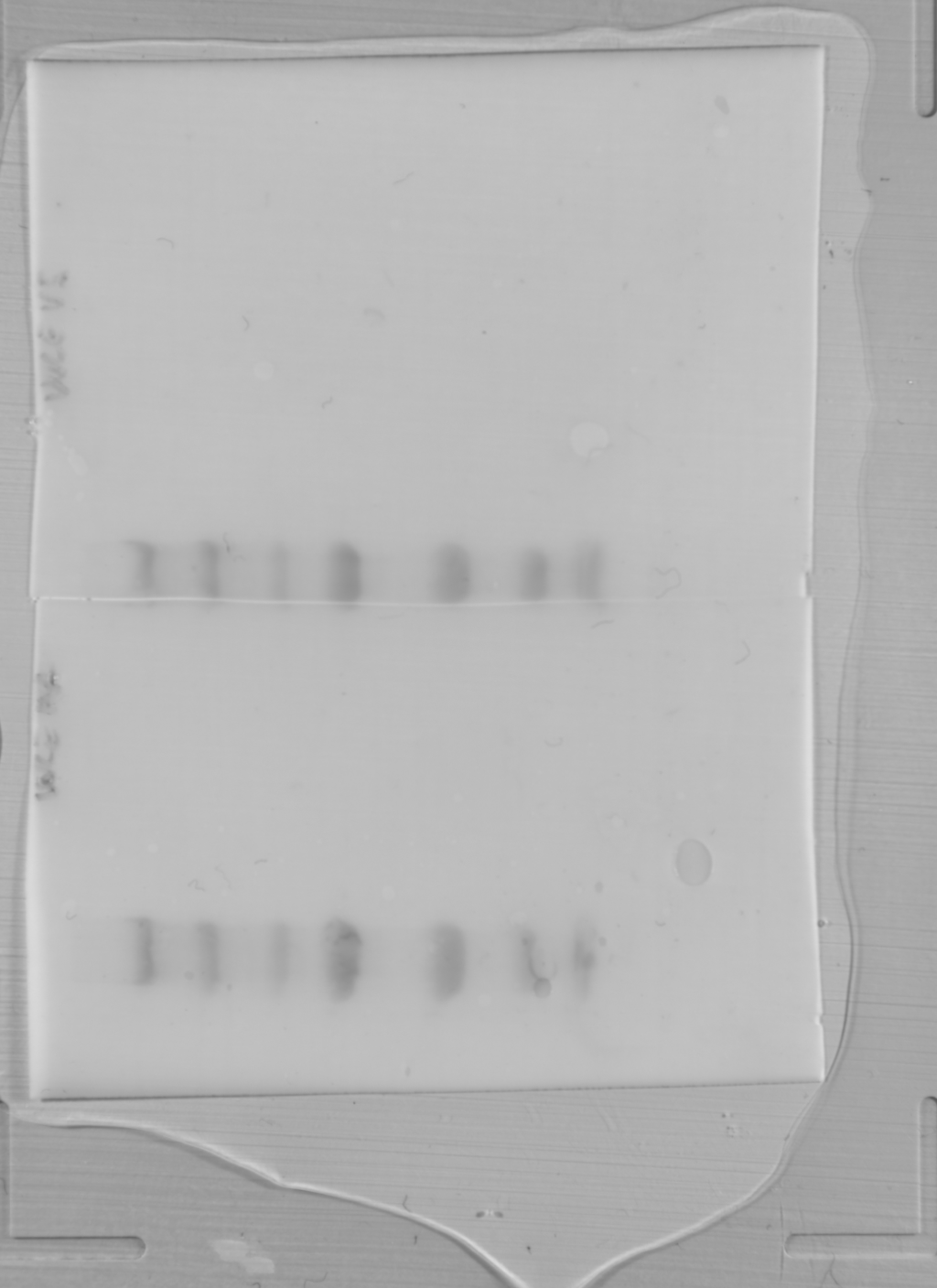

Supplement: Figure 2—figure supplement 1—source data 1. [file elife-80147-fig2-figsupp1-data1.zip › Figure 2- Figure Supplement 1- Source data 1/aV5.WCE/WCE HA & V5 2019.09.13_14.22.01-01_Ch-Marker.tif]

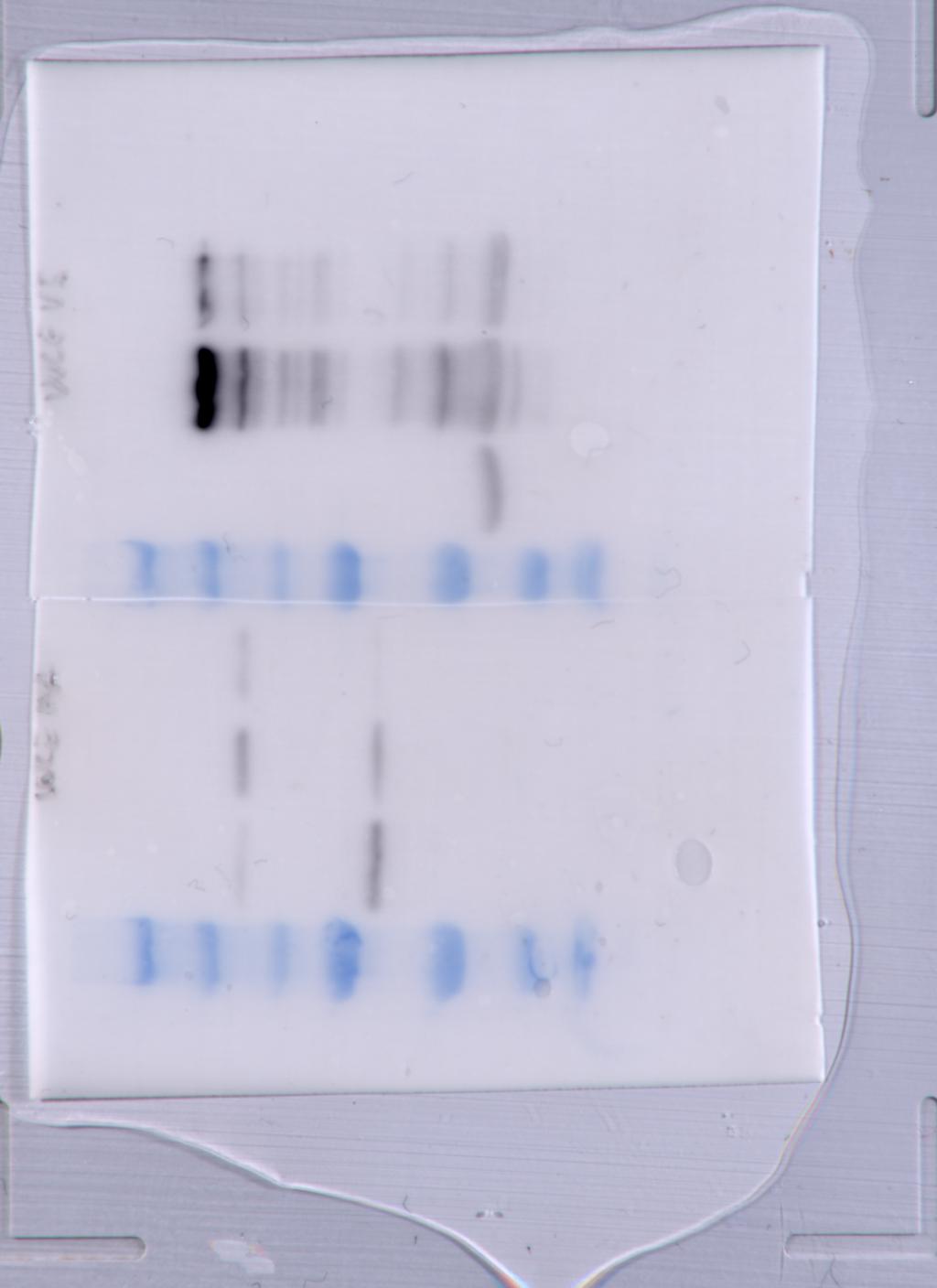

Supplement: Figure 2—figure supplement 1—source data 1. [file elife-80147-fig2-figsupp1-data1.zip › Figure 2- Figure Supplement 1- Source data 1/aV5.WCE/WCE HA & V5 2019.09.13_14.22.01-01_Ch+Marker.jpg]

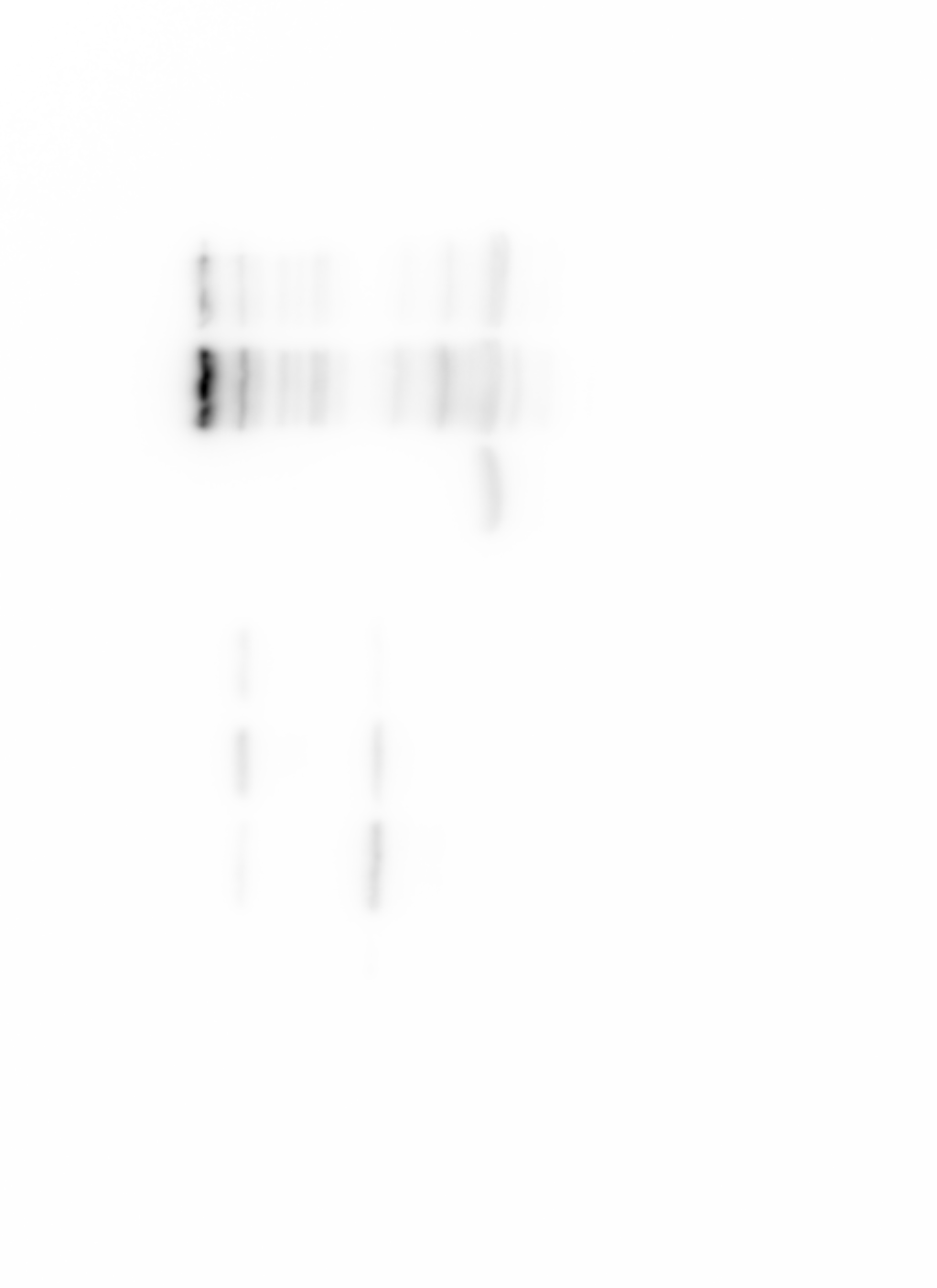

Supplement: Figure 2—figure supplement 1—source data 1. [file elife-80147-fig2-figsupp1-data1.zip › Figure 2- Figure Supplement 1- Source data 1/aV5.WCE/WCE HA & V5 2019.09.13_14.22.01-01_Ch.tif]

WCE  
αFLAG

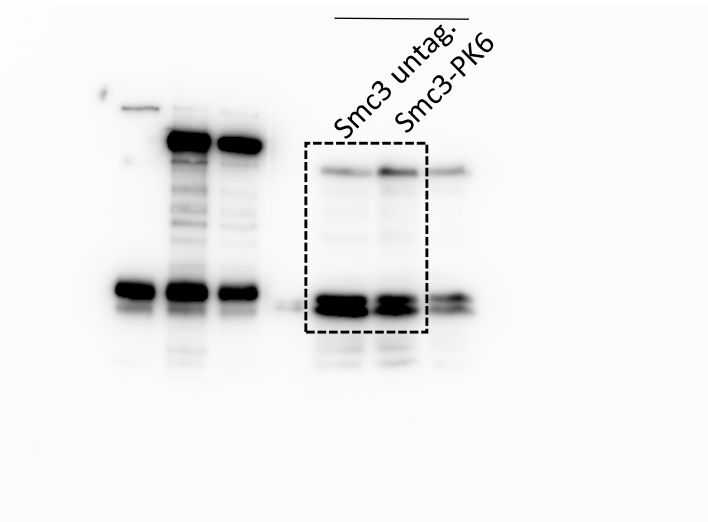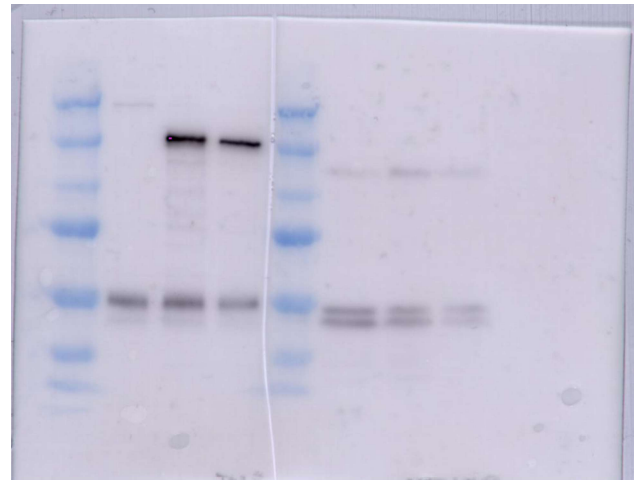

IP αSmc3-V5

IP

αV5

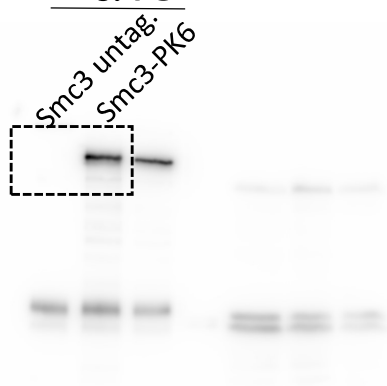

Supplement: Figure 2—figure supplement 1—source data 2. [file elife-80147-fig2-figsupp1-data2.pdf]

α PLAG

α-HA

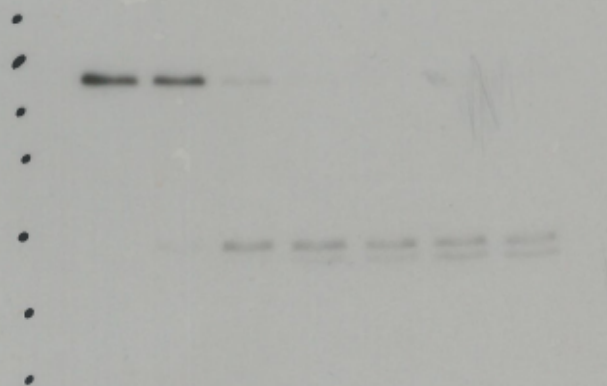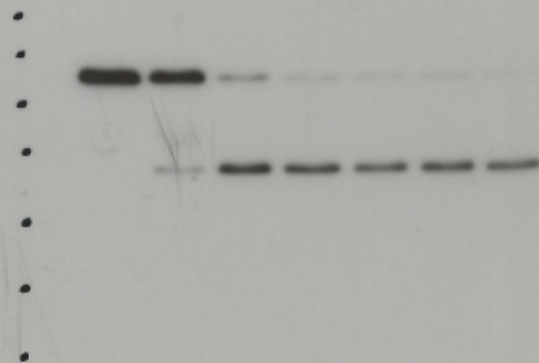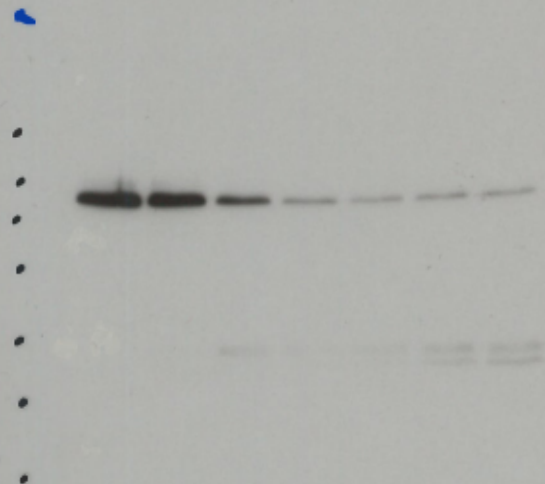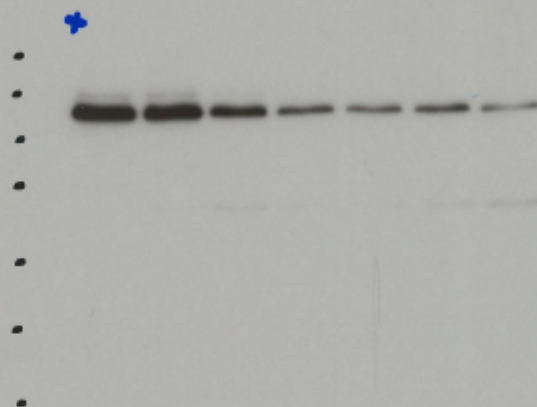

Supplement: Figure 3—source data 1. [file elife-80147-fig3-data1.zip › Figure 3- Source data 1/aFLAG.TEVF_aHA.TEVF_BottomRow.pdf]

$\alpha$ FLAG  
TEV-G

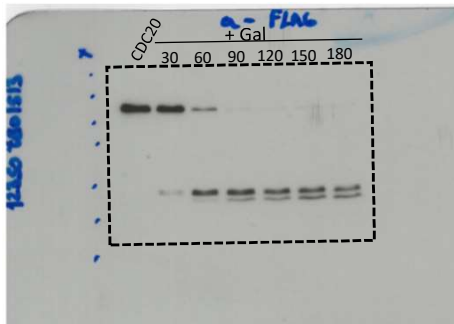

$\alpha$ HA  
TEV-G

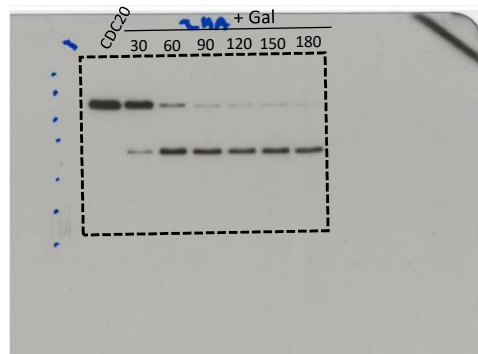

Supplement: Figure 3—source data 1. [file elife-80147-fig3-data1.zip › Figure 3- Source data 1/Figure_3-_source_data_1.pdf]

$\alpha$ FLAG  
TEV-F

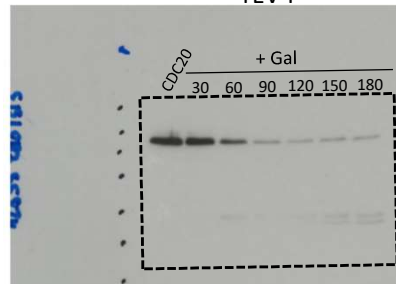

$\alpha$ HA  
TEV-F  
+ Gal

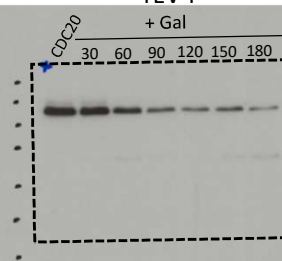

Supplement: Figure 3—source data 2. [file elife-80147-fig3-data2.zip › Figure 3- Source data 2/Figure_3-source_data_2.pdf]

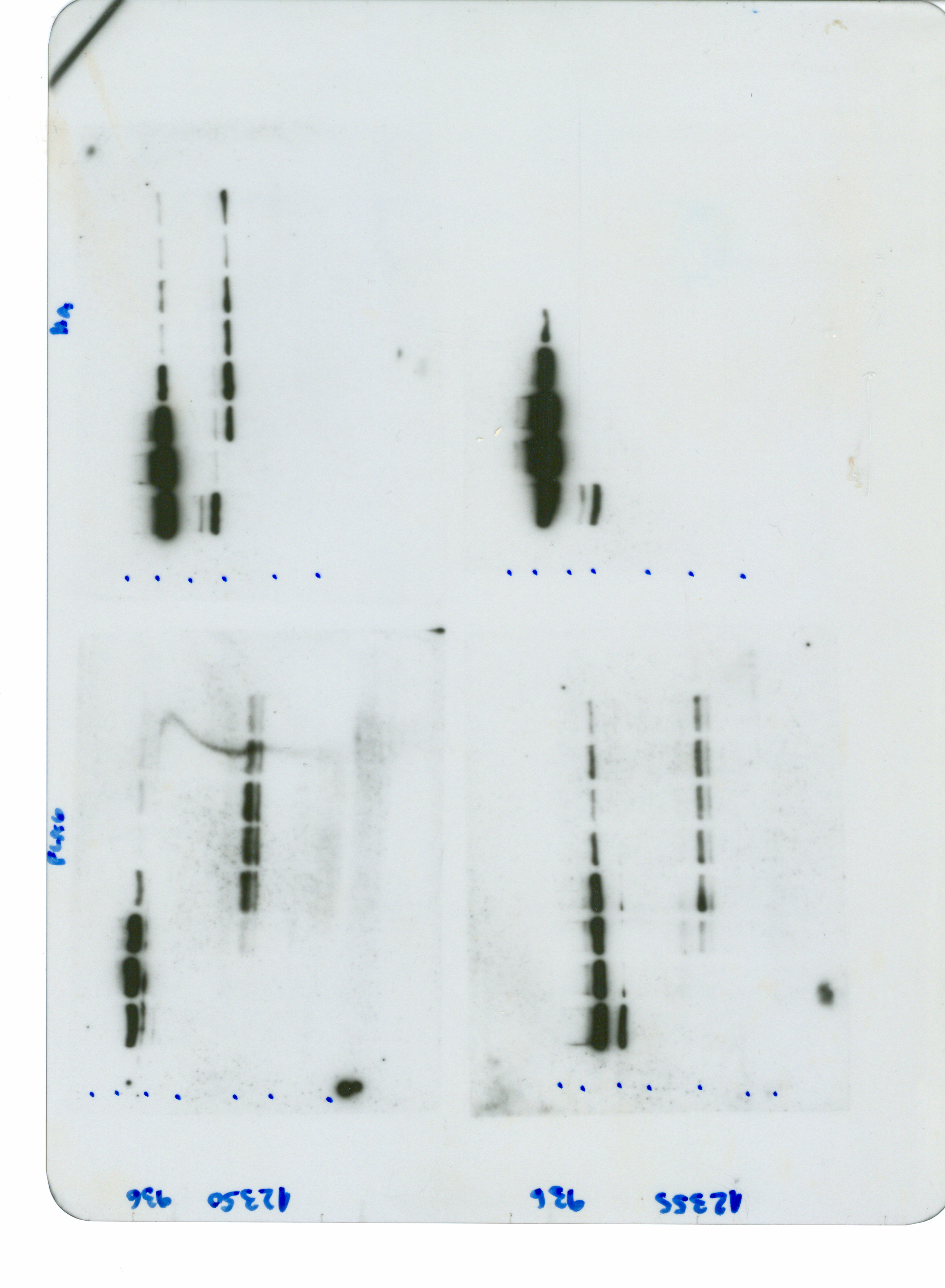

Supplement: Figure 4—figure supplement 1—source data 1. [file elife-80147-fig4-figsupp1-data1.zip › Figure 4- Figure Supplement 1- Source data 1/Fig S4_aFLAG_TopLeft.tif]

$\alpha$ FLAG

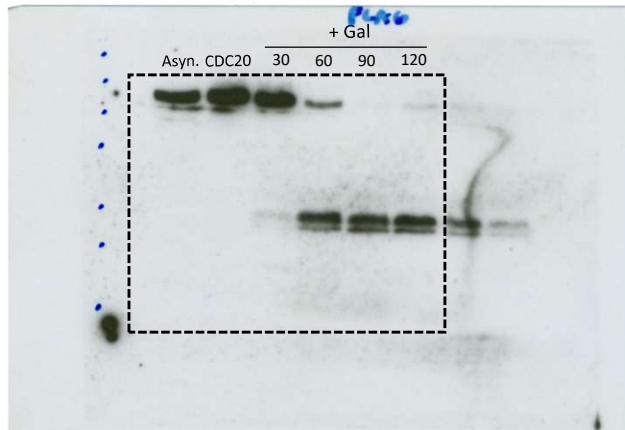

$\alpha$ HA

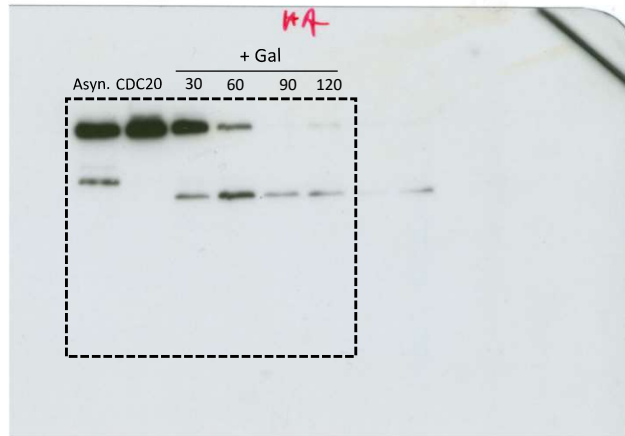

Supplement: Figure 4—figure supplement 1—source data 1. [file elife-80147-fig4-figsupp1-data1.zip › Figure 4- Figure Supplement 1- Source data 1/Figure 4_Figure_supplement_1-source_data_1.pdf]

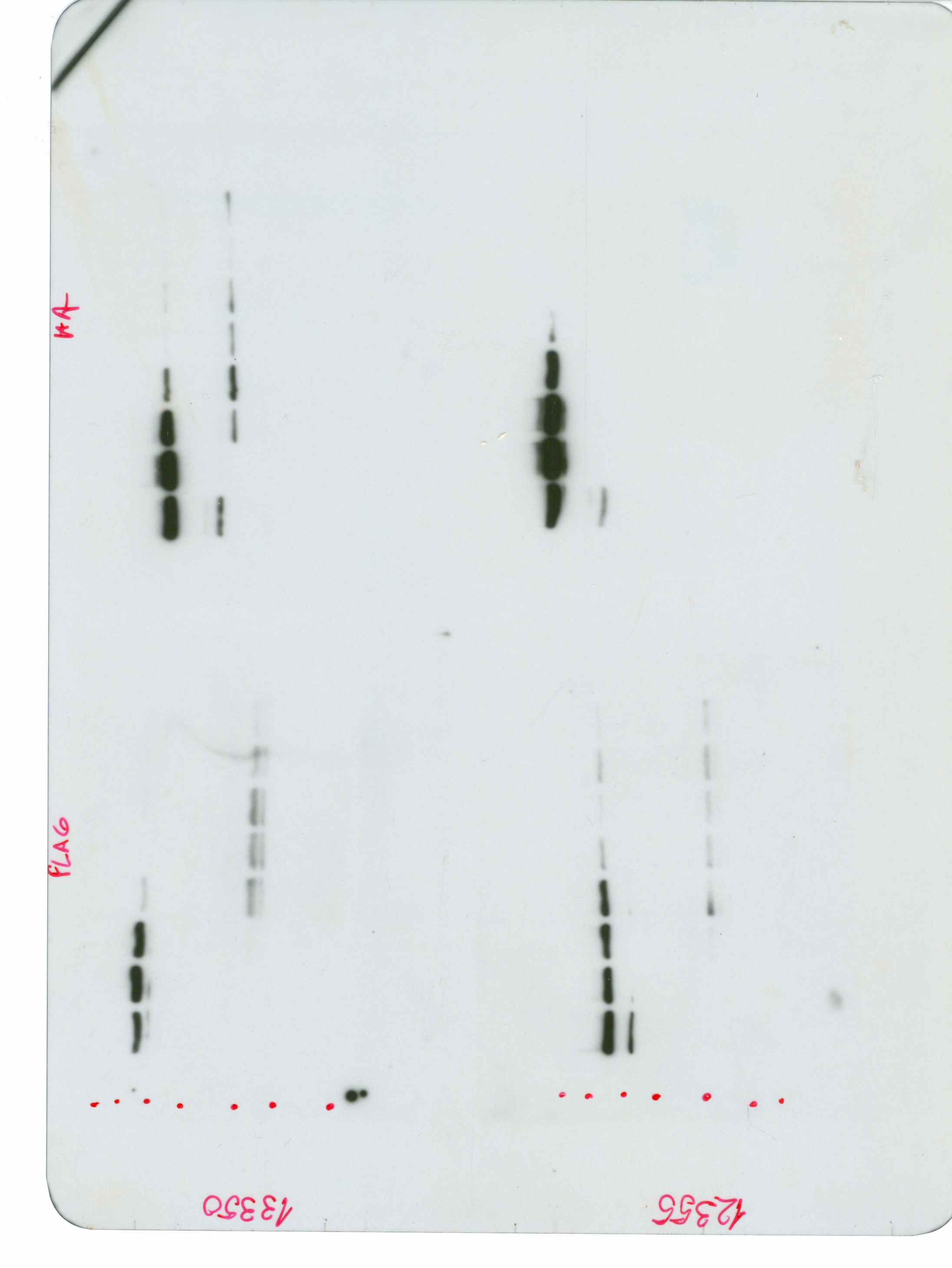

Supplement: Figure 4—figure supplement 1—source data 1. [file elife-80147-fig4-figsupp1-data1.zip › Figure 4- Figure Supplement 1- Source data 1/Fig S4_aHA_TopRigth.tif]

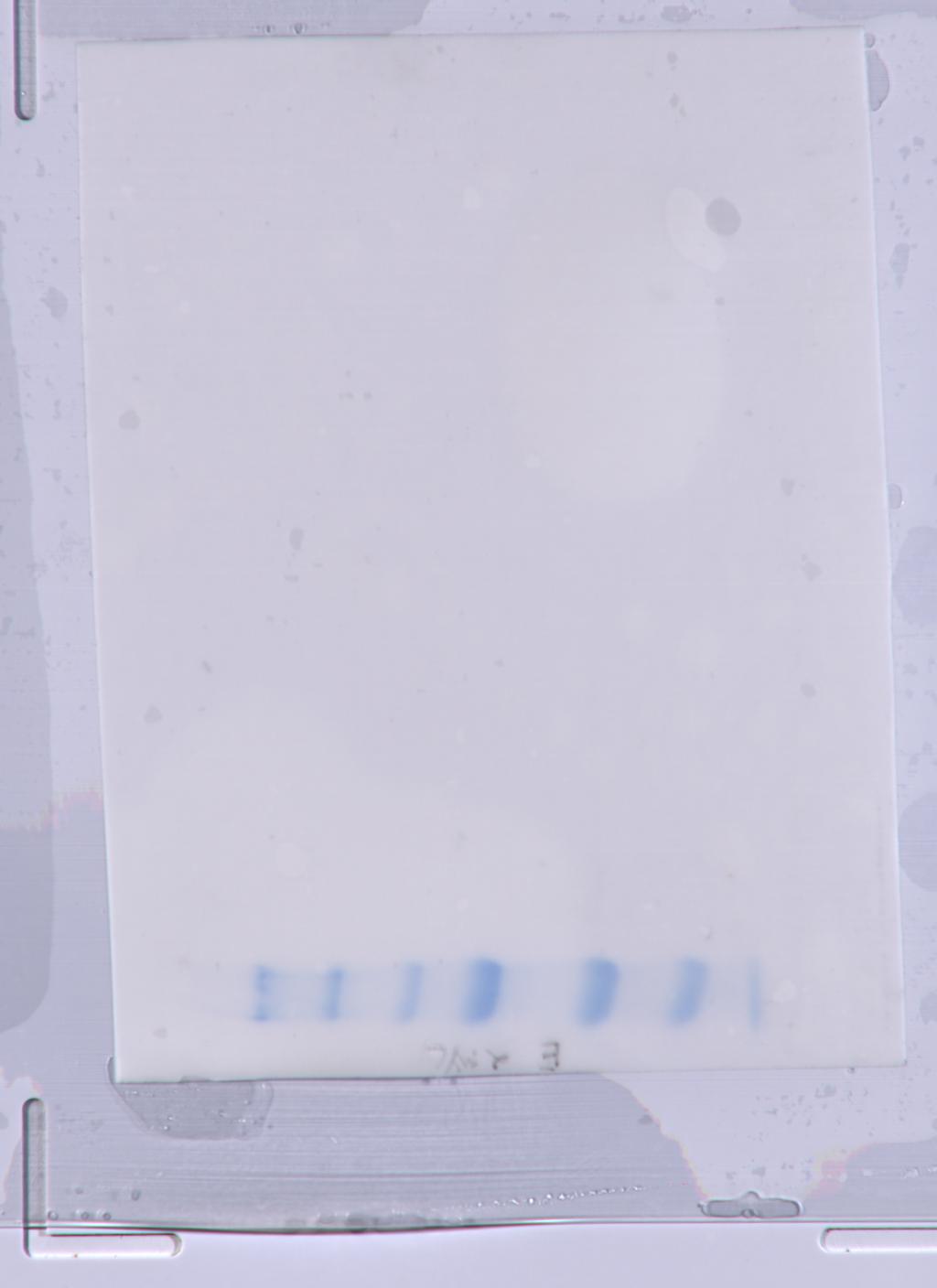

Supplement: Figure 6—source data 1. [file elife-80147-fig6-data1.zip › Figure 6- Source data 1/hic cdc15 r2 2019.02.23_14.17.06-10_Ch-Marker.jpg]

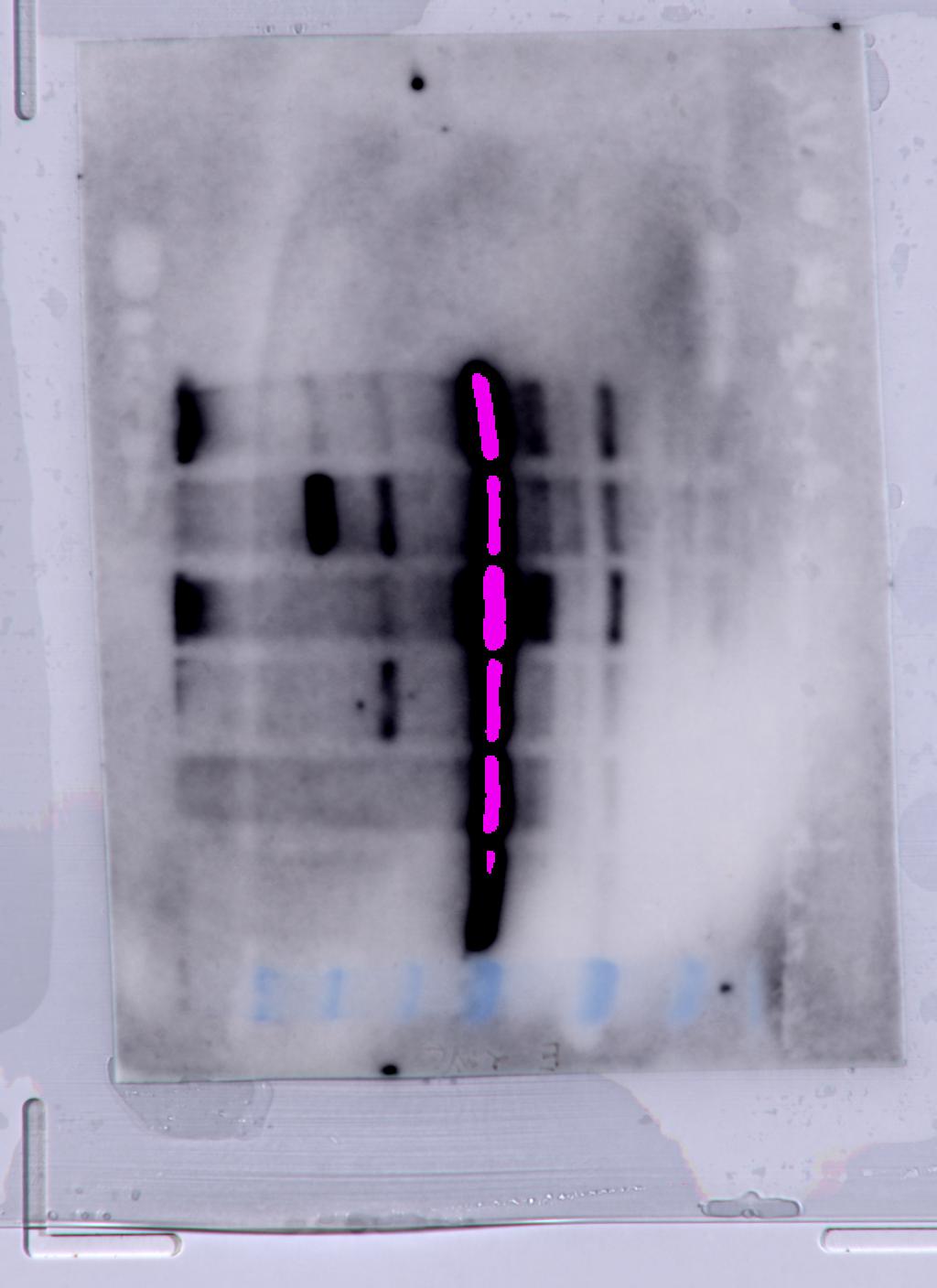

Supplement: Figure 6—source data 1. [file elife-80147-fig6-data1.zip › Figure 6- Source data 1/hic cdc15 r2 2019.02.23_14.17.06-10_Ch+Marker.jpg]

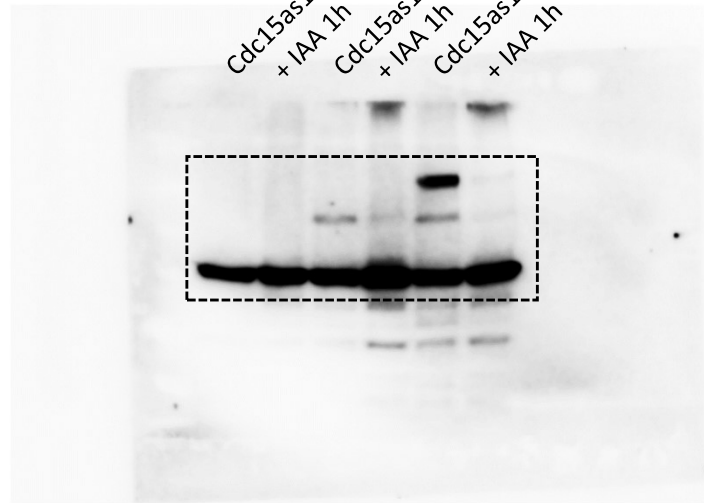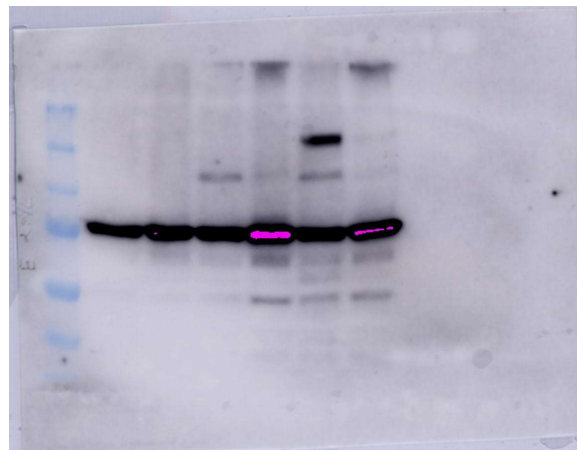

Supplement: Figure 6—source data 1. [file elife-80147-fig6-data1.zip › Figure 6- Source data 1/Figure_6-_source_data_1.pdf]

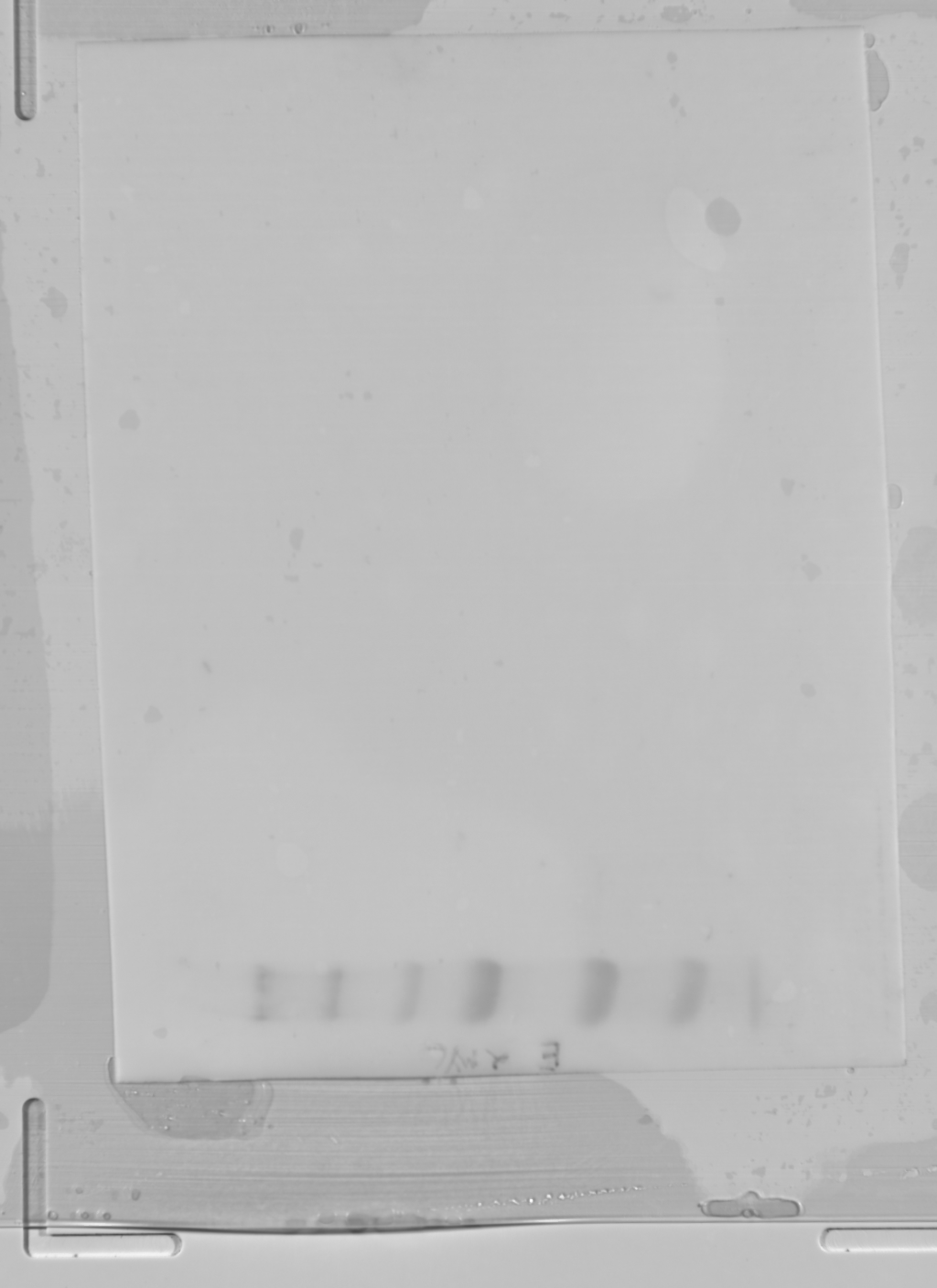

Supplement: Figure 6—source data 1. [file elife-80147-fig6-data1.zip › Figure 6- Source data 1/hic cdc15 r2 2019.02.23_14.17.06-10_Ch-Marker.tif]

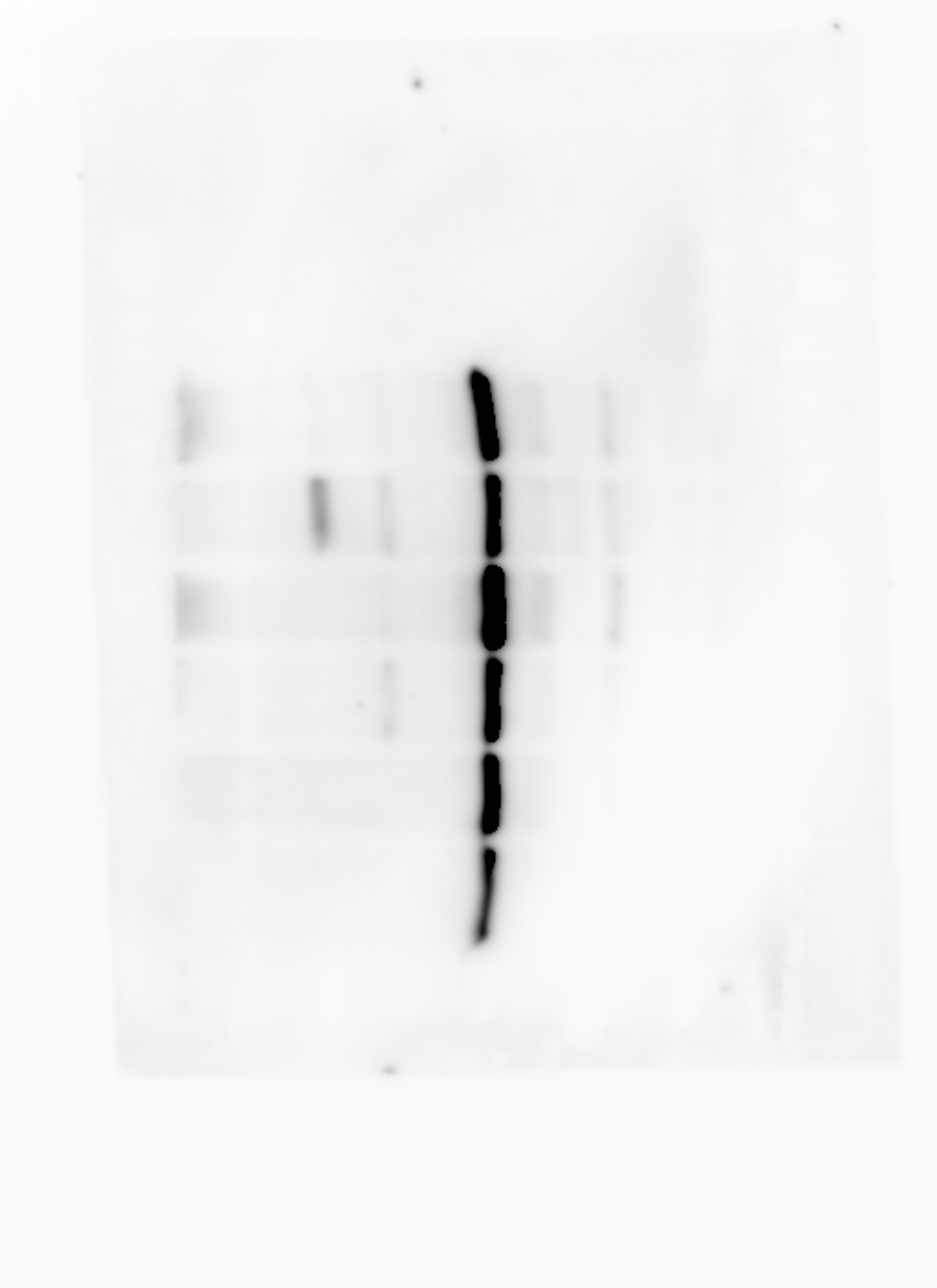

Supplement: Figure 6—source data 1. [file elife-80147-fig6-data1.zip › Figure 6- Source data 1/hic cdc15 r2 2019.02.23_14.17.06-10_Ch.tif]
