# Supplementary material for: Depletion or cleavage of cohesin during anaphase differentially affects chromatin structure and segregation
Source: eLife. 2022 Oct 5;11:e80147. doi: 10.7554/eLife.80147 (PMC9586560; doi:10.7554/eLife.80147)
Supplement: Figure 3—source data 2. [file elife-80147-fig3-data2.zip › Figure 3- Source data 2/aFLAG.TEVF_aHA.TEVG_UpperRow.pdf]

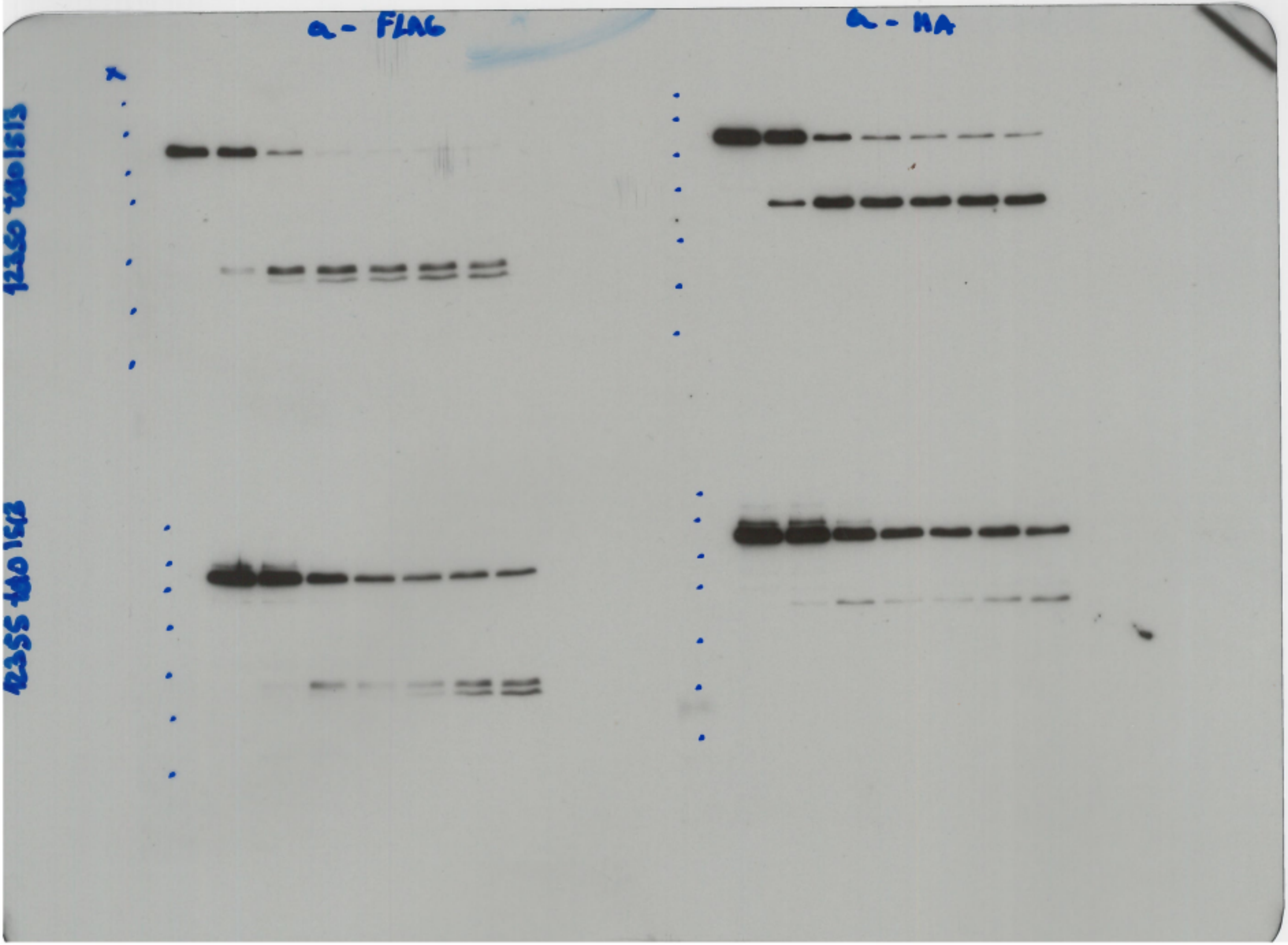
a - FLAG
a - HA
